# The Puzzle of Lost PAHs: A Step-by-Step Investigation of Pre-, During-, and Post-Solid-Phase Extraction from Water Samples

**DOI:** 10.3390/jox16060186

**Published:** 2026-09-27

**Authors:** Paula Paíga, Maria João Ramalhosa, Vanessa F. Fonseca, Bernardo Duarte, Cristina Delerue-Matos

**Affiliations:** 1 REQUIMTE/LAQV, Instituto Superior de Engenharia do Porto, Instituto Politécnico do Porto, Rua Dr. António Bernardino de Almeida, 431, 4249-015 Porto, Portugal; cmm@isep.ipp.pt; 2 MARE—Marine and Environmental Sciences Centre & ARNET—Aquatic Research Network Associated Laboratory, Faculdade de Ciências da Universidade de Lisboa, Campo Grande, 1749-016 Lisbon, Portugal; vffonseca@ciencias.ulisboa.pt (V.F.F.); baduarte@ciencias.ulisboa.pt (B.D.); 3 Departamento de Biologia, Faculdade de Ciências da Universidade de Lisboa, Campo Grande, 1749-016 Lisbon, Portugal

**Keywords:** evaporation, environmental applications, keeper solvents, method development, PAH losses, solid-phase extraction, liquid chromatography, water contamination

## Abstract

The development of an accurate method to extract polycyclic aromatic hydrocarbon (PAH) from water samples is crucial for assessing environmental contamination and protecting public health. Solid-phase extraction (SPE) remains the selected technique for extracting trace contaminants from water due to its high precision, selectivity, and reproducibility. However, extracting the 18 PAH compounds proved challenging, as not all target compounds were fully recovered. Various SPE sorbents and elution solvents were tested to minimize analyte losses, but no substantial improvements were initially observed. To address this issue, ‘keeper’ solvents were added after the elution step, resulting in moderate improvements in recovery; however, optimal results were not achieved. Further optimization efforts focused on the evaporation step, which was identified as a critical step due to the volatilization of low molecular weight PAHs during solvent drying. Refinements to this step showed improved analytical performance, ensuring the more complete and reliable recovery of all studied PAHs. The optimized method was successfully applied to diverse types of water samples, showing its applicability and effectiveness in detecting PAH contamination from multiple sources in aquatic environments. This methodology provides a valuable tool for environmental monitoring and reinforces the importance of controlling PAH pollution to protect ecosystems and human health.

## 1. Introduction

Over the years, the acceleration of industrialization and urbanization has intensified anthropogenic activities, leading to the release of various pollutants into the environment. Among these, polycyclic aromatic hydrocarbons (PAHs) are of particular concern [1]. Environmental contamination by PAHs is a major challenge in ecological and public health research [2]. These persistent organic pollutants in aquatic environments originate primarily from anthropogenic sources, including industrial processes, urban runoff, waste incineration, and the use of petroleum-based products [3,4,5,6]. While human activities are the dominant contributors, natural processes such as forest fires, volcanic eruptions, and the diagenesis of organic matter can also introduce PAHs into the environment. These compounds enter aquatic ecosystems through atmospheric deposition, direct discharge, and oil spills, leading to their accumulation in sediments and water bodies [7,8]. Among the earliest recognized and most widespread environmental carcinogens [9], PAHs are of major concern due to their toxic, mutagenic, and carcinogenic effects, which can damage DNA and increase the risk of cancer and other health problems in both aquatic organisms and humans [2]. To date, over 400 carcinogenic PAHs and their derivatives have been identified [10]. Even at low concentrations, chronic exposure to PAHs can have serious biological effects, including developmental and reproductive toxicity in marine organisms [11]. Accurate quantification of PAHs in water samples is essential to assess their environmental distribution, understand their ecological and health impacts, and develop effective control strategies [12,13]. Therefore, given their widespread presence and associated risks, it is important to conduct monitoring studies to evaluate their detection and levels in environmental water samples.

Recent advances, such as the use of novel sorbent materials, such as molecularly imprinted polymers and magnetic nanoparticles, have improved the selectivity and extraction efficiency of PAHs in aquatic matrices [14]. Additionally, automated systems, such as online solid-phase extraction (online SPE) configurations coupled directly with analytical instrumentation, have emerged to minimize sample handling and reduce analyte losses. Despite the development of alternative techniques, SPE remains a crucial method for extracting PAHs from water samples. It offers high selectivity, reproducibility, and the ability to concentrate analytes from large-volume samples [15]. SPE stands out as one of the most widely used methods for the pre-concentration and extraction of pollutants in water samples [12,16,17,18]. Consequently, the successful application of the SPE method requires careful optimization to ensure sensitivity, selectivity, and robustness across various water types [19].

The extraction of PAHs, particularly those with low molecular weight, presents considerable methodological challenges. These lighter compounds tend to be more soluble in water and volatile, making them more susceptible to loss during sample handling and pretreatment [20]. The primary complexity of PAH extraction lies in the diverse physicochemical properties of PAHs, where differences in volatility between compounds can lead to inconsistent recovery rates. Low molecular weight PAHs such as naphthalene (Naph) and acenaphthylene (Acen) can be lost during concentration steps due to their higher vapor pressure. Meanwhile, 2–3 ring PAHs show higher water solubility than their larger counterparts, which affects their retention behavior on SPE cartridges [21,22]. In addition, high-molecular-weight PAHs tend to adsorb onto glassware and sampling equipment, potentially reducing recovery rates [23,24].

Matrix effects (ME) from dissolved organic matter or suspended particles can interfere with recovery rates, affecting both the precision and accuracy of quantification [25]. These interferences often manifest as competition for binding sites on SPE sorbents, formation of PAH-organic matter complexes that alter extraction efficiency, clogging of SPE cartridges in turbid samples, and co-elution of matrix components during the elution step [16,26,27]. The choice of an adequate sorbent material is critical to the efficiency and selectivity of the extraction process, as emphasized by Maiga et al. [17] and Fontanals et al. [28]. While traditional octadecylsilane (C18) silica-based sorbents are commonly used, polymeric sorbents such as styrene-divinylbenzene copolymers have been shown to offer high recovery rates for both low and high molecular weight PAHs [17,28].

The present study included 16 priority PAHs identified by the United States Environmental Protection Agency (US EPA) [29], along with benzo[j]fluoranthene (B[j]Ft) and dibenzo[a,l]pyrene (DB[a,l]P). These 18 PAHs were selected due to their environmental relevance, toxicity, and frequent occurrence in water matrices, making them critical targets for aquatic monitoring and risk assessment [29,30]. Furthermore, the 16 PAHs designated as priority pollutants by the US EPA are classified based on their harmful effects on ecosystems and human health [29,30]. Additionally, 7 of these 16 PAHs are listed by the International Agency for Research on Cancer (IARC) as known or suspected carcinogens [31]. Specifically, benzo[a]pyrene (B[a]P) is in Group 1 (carcinogenic to humans); dibenz[a,h]anthracene (DB[a,h]A) is in Group 2A (probably carcinogenic); and benz[a]anthracene (B[a]A), benzo[b]fluoranthene (B[b]Ft), benzo[k]fluoranthene (B[k]Ft), chrysene (Chry), and indeno[1,2,3-cd]pyrene (InP) are in Group 2B (possibly carcinogenic). Among these, B[a]P poses the highest cancer risk [32]. For B[j]Ft and DB[a,l]P, the monographs published by the IARC [33] conclude that there is sufficient evidence of their carcinogenicity in experimental animals, leading to their classification as possibly carcinogenic to humans (Group 2B) for B[j]Ft [34] and probably carcinogenic to humans (Group 2A) for DB[a,l]P [35]. As mentioned in several studies [21,22,36], traditional SPE protocols often struggle to maintain consistent recovery rates for PAH compounds. This inconsistency creates a critical gap in environmental monitoring strategies. In response to this limitation, and to address the challenge of achieving good and consistent recovery rates for all target compounds, this study focuses on optimizing the SPE process for these 18 PAHs through a comprehensive evaluation of several critical parameters. A One-Factor-at-a-Time (OFAT) approach was employed to identify the critical parameters affecting PAH recoveries. The optimization strategy included several key aspects: (i) testing different SPE cartridges, (ii) adjusting the sample volume, (iii) adding an organic solvent to the sample prior to SPE, (iv) exploring different elution solvents and their mixtures, (v) using various keeper solvents after elution and before evaporation, (vi) comparing evaporation techniques—including nitrogen stream, rotary evaporation, and evaporation at room temperature (vii) evaluating the SPE washing step, (viii) testing the SPE drying step, (ix) varying pump pressure, and (x) adjusting the final evaporation volume. Furthermore, the method was validated through recovery studies, matrix-matched calibration to account for matrix effects, and a thorough evaluation of method sensitivity. The developed methodology not only improves analytical precision but also demonstrates remarkable adaptability to different water sample types. By establishing a robust protocol for PAH extraction, this study makes an important contribution to environmental monitoring efforts by offering a more reliable and versatile extraction procedure for evaluating contamination risks in aquatic ecosystems.

## 2. Bibliometric Mapping of PAH Research in Aquatic Environments Using VOSviewer

Scientists worldwide are increasingly focusing on detecting pollutants in diverse environments, and the growing volume and complexity of research data demand advanced analytical tools. In this context, bibliometric analysis has become essential for tracking research trends [37,38,39]. Due to their persistent hydrophobic nature, ubiquitous distribution, and severe carcinogenic risks, PAHs represent a class of widely studied organic pollutants of global concern [1,2,13]. As a powerful open-source tool for bibliometric mapping [38], VOSviewer supports a range of databases—including WoS, Scopus, Dimensions, and PubMed—and enables researchers to efficiently analyze and visualize scientific networks [38,39]. To explore the environmental impact of PAHs using VOSviewer software (version 1.6.21), a literature search was conducted in the ScienceDirect database in the last ten years using the search terms “PAHs” and “water environment”. The search results were screened for relevance to PAHs in aquatic environments, resulting in the selection of 460 relevant studies. The keywords occurring most frequently in the selected studies were identified, and the 14 most frequently occurring keywords were selected for the VOSviewer analysis: environmental pollution, PAHs, pollution, surface water, toxicity, water quality, distribution, fish, source, water, ecological risk assessment, source identification, risk assessment, and water pollution. The compiled database was then analyzed, and a network diagram was generated to visualize the research landscape (Figure 1). The 14 most frequently occurring keywords from the selected studies were highlighted. Keywords are designed as nodes; their size reflects the occurrence frequency, and the links indicate their interconnections. Different colors represent distinct research clusters.

Based on the cluster analysis of the VOSviewer network diagram in Figure 1, the research landscape related to PAHs in the environment is organized into four distinct clusters, each representing a specific research focus. Cluster 1, in red (PAH Pollution & Impact), includes the keywords environmental pollution, PAHs, pollution, surface water, toxicity, and water quality. This cluster focuses on the direct presence and consequences of PAH contamination in aquatic environments, emphasizing the role of PAHs as pollutants in surface waters and underscoring their associated toxicity and adverse effects on water quality. Cluster 2, in green (PAH Fate & Source in Water), includes the keywords distribution, fish, source, and water. This group analyses the behavior and origin of PAHs in aquatic environments, investigating their distribution across water bodies, their potential impact on aquatic organisms, and the identification of sources contributing to water contamination. Cluster 3, in blue (Ecological Risk & Source Identification), includes the keywords ecological risk assessment and source identification. It specifically addresses the evaluation of risks posed by PAH contamination to ecosystems, along with the methods used to trace the origins of these pollutants. Finally, Cluster 4, in yellow (General Risk in Water Pollution), includes the keywords risk assessment and water pollution. This cluster provides a broader perspective on the overall risks associated with PAHs in the context of general water pollution, highlighting the need to assess the potential hazards that PAHs pose to water resources. The VOSviewer network reveals the multifaceted nature of PAH research in aquatic environments, ranging from a fundamental understanding of pollution and its impacts to investigating the fate, sources, and risks posed to ecosystems and water quality. Moreover, current research on improving PAH extraction methods directly contributes to the collection of more accurate data in these key areas.

## 3. Materials and Methods

### 3.1. Sampling Campaign

Prior to sample collection, high-density polyethylene bottles were rinsed first with ultra-pure water and then with the respective sample. Drinking water, used for method validation, was purchased from a local market. Surface water and wastewater samples were used for both method validation and for analysis of real samples. The environmental water samples were collected in northern and central Portugal during spring 2026. A total of 25 environmental water samples were collected, comprising ocean water samples (*n* = 3), river water samples (*n* = 11), WWTP effluent samples (*n* = 6), and WWTP influent samples (*n* = 5).

Surface water samples were collected as grab samples (1 L), with each sample consisting of a single aliquot collected at the respective sampling site. Wastewater samples were collected as 24 h composite samples (1 L), consisting of multiple aliquots collected over time to represent the average characteristics of the wastewater. Wastewater samples were collected by the personnel responsible for the operation in each WWTP. The studied WWTPs employ preliminary treatment followed by primary sedimentation and secondary treatment based on an activated sludge process with extended aeration.

Immediately after collection, the surface water and wastewater samples were placed in an ice-cooled container and transported to the laboratory. Upon arrival at the laboratory, the samples were vacuum-filtered through a 0.45 µm nylon membrane with a diameter of 47 mm (FilterBio, Nantong City, People’s Republic of China) before SPE and subsequent chromatographic analysis.

### 3.2. Eluents, Solvents, Materials, and Reagents

For the conditioning and equilibration of the SPE cartridges, ultra-pure water (resistivity: 18.2 MΩ·cm) was obtained from a Milli-Q water purification system (Millipore, Molsheim, France) and methanol CHROMASOLV™, gradient grade for high-performance liquid chromatography (HPLC) with an assay (GC) of ≥99.9% and dichloromethane puriss, ACS reagent, Reag. ISO (assay ≥ 99.9%) were purchased from Honeywell (Seelze, Germany).

The eluents used in the HPLC system were ultra-pure water and acetonitrile of chromatographic grade, Hipersolv CHROMANORM, Supergradient, Reg. Ph.EUR. (assay GC > 99.99%), which were obtained from VWR Chemicals (Rosny-sous-Bois, Cedex, France). All chromatographic solvents were filtered through a 0.22 μm nylon membrane filter (Fioroni Filters, Ingré, France) using a vacuum pump KNF LABOPORT Mini Diaphragm Vacuum Pump N 816.3 with a fine adjustment head and vacuum gauge (Freiburg, Germany). The solvents were degassed for 15 min in an ultrasonic bath (Sonorex Digital 10P, Bandelin DK 255P, Berlin, Germany).

In the SPE methodology, several solvents were tested to ensure the complete recovery of the target PAHs. The elution solvents used included dichloromethane (Puriss, ACS reagent, Reag. ISO, assay ≥ 99.9%), isopropanol (Chromasolv™ LC-MS, assay ≥ 99.9%), methanol (LC-MS Chromasolv™, assay ≥ 99.9%), acetonitrile (Chromasolv™ gradient for HPLC, assay ≥ 99.9%), and acetone (Envisolv^®^, assay GC ≥ 99.8%), all purchased from Honeywell Riedel-de Haën™ (Seelze, Germany), *n*-hexane (for analysis, assay GC ≥ 99.9%), tetrahydrofuran (ACS, Reag. Ph. Eur., Reag. USP, stabilized with BHT, assay ≥ 99.9%), and ethyl acetate (ACS, Reag. Ph. Eur., Reag. USP, assay ≥ 99.9%) were obtained from Carlo Erba (Val de Reuil, Cedex, France), while cyclohexane (Lichrosolv^®^, assay ≥ 99.9%) was acquired from Merck (Darmstadt, Germany), and pentane (reagent grade, assay ≥ 98%) was obtained from Sigma-Aldrich (Steinheim, Germany). As keeper solvents, methanol (LC-MS Chromasolv™, assay ≥ 99.9%) and isopropanol (Chromasolv™ LC-MS, assay ≥ 99.9%) were purchased from Honeywell Riedel-de Haën™ (Seelze, Germany), isooctane (ACS-for analysis, assay GPC ≥ 99.5%) was obtained from Carlo Erba (Val de Reuil, Cedex, France), and *N,N*-dimethylformamide (ACS reagent, assay ≥ 99.8%), hexanol (reagent grade, assay ≥ 98%), toluene (Puriss p.a., ACS reagent, Reag. ISO, Reag. Ph. Eur., assay ≥ 99.7%), and octanol (ReagentPlus^®^, assay ≥ 99%) were acquired from Sigma-Aldrich (Darmstadt, Germany).

Strata-PAHs (1.5 g, 6 mL) and Strata-X (200 mg, 3 mL) SPE cartridges, used as sorbents for the extraction of PAHs from water samples, were obtained from Phenomenex Inc. (Torrance, California, USA). A Macherey-Nagel™ CHROMABOND™ Complete Vacuum Manifold with 12 positions (Düren, Germany) was used to perform the multiple extractions.

Before chromatographic analysis, the sample extracts and standards were filtered through 0.22 μm PTFE syringe filters (Specanalitica, Carcavelos, Portugal).

### 3.3. Polycyclic Aromatic Hydrocarbons

Certified EPA 610 mixture standard (Naph, Acen, acenaphthene (Ace), fluorene (Flu), phenanthrene (Phe), anthracene (Ant), fluoranthene (Fln), pyrene (Pyr), B[a]A, Chry, B[b]Ft, B[k]Ft, B[a]P, DB[ah]A, benzo[ghi]perylene (B[ghi]P), and InP and B[j]Ft, DB[a,l]P) were acquired from Supelco (Bellefonte, PA, USA). Intermediate solutions were prepared in acetonitrile by diluting the PAH stock standard solutions. Working standard solutions were then prepared by further dilution of the intermediate solutions. All solutions were stored at –20 °C until use.

### 3.4. Chromatographic Analysis

The chromatographic analysis of PAHs was conducted according to the method described by Ramalhosa et al. [4] using a Shimadzu LC system (Shimadzu Corporation, Kyoto, Japan), with a degasser (DGU-20A5R), a binary delivery pump (LC-20AD), an autosampler (SIL-20AHT), a system controller (CBM-20A), and a photodiode array (PAD, SPD-M20A) and a fluorescence (FLD, RF-10AXL) detectors connected in series. Chromatographic data were acquired using LabSolutions software, version 5.82 (Shimadzu Corporation, Kyoto, Japan). Separation and detection of target PAHs were performed using a C18 reverse-phase column (CC150/4 Nucleosil 100-5 C18 PAH; 5 μm particle size, 150 × 4.0 mm) acquired from Macherey-Nagel (Düren, Germany). Ultra-pure water (eluent A) and acetonitrile (eluent B) were used as the mobile phase with the following optimized gradient program: 50% eluent B for 5 min, a linear increase to 100% over 15 min, maintenance at 100% for 16 min, and a return to 50% B within 2 min. The chromatographic run was performed over 45 min at a constant flow rate of 0.8 mL/min [4]. The column oven temperature was maintained at 25 °C, and the injection volume was 20 μL. The excitation/emission wavelength pairs used for FLD detection were as follows: the FLD was initially set to 260/315 nm for the detection of Naph, Ace, and Flu. At 15.90 min, the excitation/emission wavelengths were changed to 260/366 nm for Phe. At 17.40 min, the wavelengths were changed to 260/430 nm for the detection of Ant, Fln, Pyr, B[a]A, Chry, B[b]Ft, B[j]Ft, B[k]Ft, B[a]P, DB[ah]A, B[ghi]P, and DB[a,l]P. At 30.00 min, the wavelengths were changed to 290/505 nm for the detection of InP. During the equilibration period, at 38.00 min, the FLD was returned to 260/315 nm. Due to its weak fluorescence response, PAH Acen was detected using a PAD detector at 229 nm.

### 3.5. Analytical Method Validation

Analytical method validation is essential for ensuring the accuracy, reliability, and reproducibility of the results [40]. Accordingly, linearity, limits of detection (LOD), limits of quantification (LOQ), method detection limits (MDLs), method quantification limits (MQLs), intra- and inter-day precision, recovery, and ME were evaluated.

## 4. Results and Discussion

### 4.1. Chromatographic Separation of the Target PAHs

The chromatographic separation of the selected PAHs was performed as described in Section 3.4. The effectiveness of the applied method is demonstrated in Figure 2, which presents the elution profile of a standard mixture containing all 18 target analytes. The black trace in the chromatogram represents the compounds detected by the FLD and illustrates the separation achieved for 17 PAHs. The blue trace simultaneously highlights the signal corresponding to Acen, as detected by the PAD detector.

The analyzed standard had the following concentrations: Naph (99.9 µg/L), Acen (210 µg/L), Ace (99.9 µg/L), Flu (19.7 µg/L), Phe (10.0 µg/L), Ant (10.0 µg/L), Fln (19.7 µg/L), Pyr (9.70 µg/L), B[a]A (9.8 µg/L), Chry (9.8 µg/L), B[b]Ft + B[j]Ft (40 µg/L), B[k]Ft (9.90 µg/L), B[a]P (10.0 µg/L), DB[a,l]P (20.0 µg/L), DB[ah]A (19.9 µg/L), B[ghi]P (19.8 µg/L), and InP (10.0 µg/L)). The corresponding retention times were: Naph (10.366 min), Acen (12.362 min), Ace (14.971 min), Flu (15.510 min), Phe (16.762 min), Ant (18.063 min), Fln (19.259 min), Pyr (20.123 min), B[a]A (22.903 min), Chry (23.581 min), B[b]Ft + B[j]Ft (25.489 min), B[k]Ft (26.545 min), B[a]P (27.523 min), DB[a,l]P (28.288 min), DB[ah]A (29.227 min), B[ghi]P (30.375 min), and InP (31.629 min).

The detection and separation of all target PAHs in the chromatogram (Figure 2) highlight the effectiveness of the method and confirm its suitability for the accurate analysis of environmental samples.

### 4.2. Preliminary Studies: SPE Cartridges and Sample Volume

To systematically address the extraction of the 18 target PAHs from water, a step-by-step OFAT approach was employed to investigate the parameters affecting analyte recovery. To guide this optimization process, the target benchmark acceptance criteria were defined as a recovery > 70% and an RSD < 15%.

Both SPE cartridges and sample volumes were evaluated in preliminary studies due to their critical roles in the retention and recovery of target analytes. The choice of SPE cartridge is crucial because the sorbent chemistry determines the selectivity, retention efficiency, and recovery of PAHs from water samples. Moreover, since environmental pollutants typically occur at trace levels in aquatic environments, adequate preconcentration is essential to ensure reliable detection. Therefore, the sample volume must be appropriate to ensure effective analyte recovery without exceeding the sorbent’s capacity, as this could lead to breakthrough and analyte loss. Consequently, two types of SPE cartridges (Strata-PAH and Strata-X) and two sample volumes (100 and 250 mL) were tested.

#### 4.2.1. SPE Cartridges

For the study of the influence of SPE sorbent, two cartridges were compared. The Strata-PAH cartridge is a polymeric sorbent that has been specially modified to enable the selective recovery of the 16 PAHs listed by the US EPA from environmental samples [41], in accordance with the EPA Method 550.1 [42]. As highlighted in the Phenomenex application note, a key advantage of this method is its ability to simultaneously remove humic acids from the extract, thereby improving PAH recovery [41]. In contrast, the reversed-phase polymeric sorbent, Strata-X, is characterized by its strong retention of neutral, acidic, and basic compounds [43]. Consequently, the different sorbent masses (1.5 g for Strata-PAH versus 200 mg for Strata-X) were not a target variable of this study. Instead, the focus was to evaluate and compare the overall extraction performance of these two commercially available cartridge configurations under their respective established application protocols.

Tests were performed to compare the performance of the two SPE cartridges. Test 1 followed the procedure described in Phenomenex Application Note TN-0042 [41] using Strata-PAH, while Test 2 followed the procedure in Phenomenex Application Note CN-021 [43] using Strata-X. The basis for Test 3 was the established broad applicability of Strata-X [44,45,46,47,48,49], coupled with the authors’ prior research using Strata-X to extract pharmaceuticals [45,46,50,51,52,53] and pesticides [54] from water samples from different environments.

Table S1 (Supplementary Materials) provides details of the conditions used for each test, outlining the parameters for conditioning, sample volume, washing, drying, elution, and evaporation. These tests were performed using 100 mL of drinking water (DW) and 100 mL of river water (RW) samples, and the recovery percentages for all the studied PAHs (*n* = 1) are shown in Figure 3. The results obtained are discussed in the following paragraphs.

As shown in Figure 3, the recovery profiles of the PAH compounds varied between tests. Test 1 yielded the highest recoveries, while Test 3 achieved the lowest recoveries. Among the compounds evaluated, Fln and Pyr demonstrated the highest recoveries in Test 1, with values of approximately 80% in DW and 90% in RW. In Test 2, the recoveries for both Fln and Pyr were approximately 60% in DW and approximately 70% in RW. The PAHs B(a)A and Chry showed recoveries of approximately 70% in Test 1 and 60% in Test 2 in DW, and approximately 60% in Tests 1 and 2 in RW. In the case of Phe and Ant, these PAHs demonstrated recoveries generally below 40% in Tests 1 and 2, while the high molecular weight PAHs had recoveries below 60% in Test 1 and below 50% in Test 2.

It is important to highlight that, with the exception of Fln and Pyr, most PAHs showed slightly lower recoveries in RW samples compared to DW samples. This is likely due to the matrix complexity, with higher levels of interfering substances present in RW compared to the DW matrix. Test 3 showed the lowest extraction efficiency among all tests. In RW samples, seven compounds were not retained (Ace, B[*a*]A, Chry, DB[*a,l*]P, DB[*ah*]A, B[*ghi*]P, and InP), and for the remaining PAHs, the maximum recovery obtained was 32.0% for Phe. In DW samples, thirteen compounds were not retained (Naph, Flu, Ant, B[*a*]A, Chry, B[*b*]Ft + B[*j*]Ft, B[*k*]Ft, B[*a*]P, DB[*a,l*]P, DB[*ah*]A, B[*ghi*]P, and InP), and the remaining PAHs showed lower recoveries than in RW samples.

To evaluate the statistical significance of the extraction performance across the preliminary procedures, one-way Analysis of Variance (ANOVA) followed by Tukey’s Honestly Significant Difference (HSD) post hoc test (α = 0.05) was conducted. The analysis confirmed statistically significant differences among the three extraction tests in both drinking water (F = 22.85, *p* < 0.001) and river water (F = 14.20, *p* < 0.001) matrices. Post hoc comparisons demonstrated that Test 3 (Strata-X with methanol elution) yielded significantly lower overall recoveries than both Tests 1 and 2 (*p* < 0.001), confirming that pure methanol lacks adequate elution strength for hydrophobic PAHs. Furthermore, Test 1 (Strata-PAH with dichloromethane elution) generally achieved significantly higher recoveries (*p* < 0.05) than Test 2 (Strata-X with dichloromethane elution) for mid-to-high molecular weight PAHs, with peak recoveries observed for Fln and Pyr in drinking water (~80% in Test 1 vs. ~60% in Test 2) and river water (~90% in Test 1 vs. ~73% in Test 2). Conversely, low molecular weight PAHs (Naph, Acen, Ace, and Flu) exhibited consistently low recoveries (<15%) across all three preliminary tests without statistically significant differences (*p* > 0.05), reinforcing that sorbent selection alone does not overcome volatilization losses during concentration.

Although the same SPE cartridge (Strata X) was employed in Tests 2 and 3, the elution solvent differed between the two methods (Table S1, Supplementary Material). Dichloromethane was used in Test 2, whereas methanol was used in Test 3. The lower PAH recoveries observed in Test 3 suggest that methanol does not provide sufficient elution strength for the target compounds. To assess whether adding a stronger solvent improves recovery, an additional experiment (Test 4) was conducted using a methanol–dichloromethane mixture (1:1, *v*/*v*). Recoveries obtained using Strata-X cartridges under the three elution conditions, 100% dichloromethane (Test 2), 100% methanol (Test 3), and methanol–dichloromethane (1:1, *v*/*v*) (Test 4), were compared. The results are presented in Figure S1 (Supplementary Material).

As expected, an increase in recovery was observed with the addition of dichloromethane to methanol in the elution step (Test 4 vs. Test 3), and this improvement was consistent in both the DW and RW matrices. Maximum recovery was obtained for Pyr (34.2%) using DW, and 33.5% (Phe), 31.3% (Fln), and 36.8% (Pyr) in RW samples. Using Strata-X cartridges, higher recoveries were found using only dichloromethane in the elution step (Test 2). The recovery trend using Strata-X followed the order: Test 2 > Test 4 > Test 3. These results indicate that methanol alone does not provide sufficient elution strength to effectively break the interactions between PAHs and the Strata X sorbent, resulting in lower recoveries. Thus, the extraction procedure used in Test 3, originally developed for pharmaceuticals [45,46,50,51,52,53] and pesticides [54] is not the best option for the extraction of PAHs from water samples.

#### 4.2.2. Sample Volume

Sample volume is a critical variable in SPE, not only because it influences the recovery efficiency, but also due to its effects on: (i) concentration factor, (ii) matrix interferences, and (iii) extraction time. Evaluating these factors is essential for optimizing method performance and ensuring its reliability for routine environmental monitoring.

Owing to the very low PAH recoveries observed in Test 3 (Figure 3) for all studied PAHs when DW samples were used, the evaluation of sample volume was conducted only using RW to ensure more robust results under the tested conditions. Thus, to assess the sorbent capacity, Tests 1, 2, and 3 (Table S1, Supplementary Materials) were performed using sample volumes of 100 mL and 250 mL with RW samples. The recovery percentages (*n* = 1) are presented in the bar graph shown in Figure S2 (Supplementary Materials).

Consistent with the findings presented in the previous subsection, the highest recoveries were obtained in Test 1, followed by Test 2, while Test 3 yielded the lowest recoveries. The PAHs Naph, Acen, Ace, and Flu consistently showed recoveries below 15% across all tests. Comparable results were obtained using sample volumes of 100 and 250 mL.

### 4.3. Pre- and Post-Solid-Phase Extraction

The compounds Naph, Acen, Ace, and Flu consistently showed very low recoveries (<15%) under different experimental conditions in the tested matrices, highlighting the difficulty of extracting more volatile PAHs. These results also suggest that the method is not yet fully optimized and does not clearly indicate the step at which losses occur. This has important implications for environmental monitoring, as poor extraction may lead to underestimation of PAH concentrations and inaccurate risk assessment.

According to Kumar et al. (2022) [55], analyte losses can occur at any stage of sample preparation, pre-extraction, during extraction, post-extraction, or due to ME, highlighting the importance of identifying the step at which the compounds are lost. A schematic flowchart of the extraction procedure, from sample preparation to chromatographic analysis, is presented in Figure S3 (Supplementary Materials). A step-by-step evaluation of the process was conducted following the OFAT approach, using the previously established target acceptance criteria (recovery > 70% and RSD < 15%). The “during extraction” step was assessed in preliminary studies presented in Section 4.2 (Section 4.2.1—SPE cartridge and Section 4.2.2—Sample volume), while the pre- and post-extraction steps were evaluated in the following subsections. Test 1 using the Strata-PAH cartridge, which showed the highest recovery efficiencies for most PAHs, was selected for further investigation.

#### 4.3.1. Sample Pre-Treatment Prior to SPE

If the samples are not properly prepared, the evaluated SPE procedure may not ensure complete retention of analytes, highlighting the critical importance of the pre-treatment. Owing to the low solubility of PAHs in water, particularly for higher-molecular-weight compounds, Fu and Zou (Agilent Technologies, Inc.) [56] recommend the addition of an organic solvent to the water samples, using 20% methanol to improve solubility and extraction efficiency. Similarly, Application Note TN-0042 [41] from Phenomenex uses 25% acetonitrile in water samples prior to loading them onto the Strata-PAH cartridge to enhance the retention and recovery of PAHs during SPE. In the present study, the procedure described in Application Note TN-0042 [41] was followed, and 25% acetonitrile was added to both DW and RW samples prior to extraction. A 100 mL sample (75 mL water sample and 25 mL acetonitrile) was used and the procedure of Test 1 (*n* = 1) was followed (Table S1, Supplementary Materials). The obtained results are shown in Figure 4.

The addition of 25% of acetonitrile to the water samples showed varying effects across the target compounds depending on their molecular weight. For the low molecular weight PAHs, recovery remained consistently low (<10%) in both DW and RW samples, regardless of the presence of the organic solvent. For Phe and Ant, recoveries remained largely unchanged in DW, with only a slight improvement observed in RW upon solvent addition, maintaining moderate recovery values of approximately 40% in both matrices. Similarly, for Fln and Pyr, a slight increase of 12% in DW was observed when acetonitrile was added, whereas their recoveries in RW remained unchanged, ranging overall from 79.2% to 95.4%. In contrast, the addition of organic solvent to the water samples improved high molecular weight PAH recoveries in both types of water samples. In DW, recoveries increased by 34.1–47.8%, rising from an initial range of 49.6–73.2% to 94.7–109%. Similarly, in RW, recoveries increased by 36.6–56.0%, rising from an initial range of 32.8–60.6% to 88.8–104% for PAHs 9 to 18 (B[a]A to InP). These results are consistent with those reported by Fu and Zou [56], who found that adding 20% organic solvent to the tested water sample increased the recovery of high molecular weight PAHs by 35–49%.

#### 4.3.2. Post-Extraction

The post-extraction step includes evaporation, redissolution of the residue obtained after evaporation, and filtration of the extract using a syringe filter (Figure S3, Supplementary Materials) prior to chromatographic analysis. Since filtration and solvent selection are more straightforward to optimize, the study was conducted in reverse order: filtration of the extract, selection of the redissolution solvent, and finally the study in the evaporation step.

##### *Filtration of the Extract* 

Kulkarni et al. [57] emphasized the critical importance of filtering both samples and standards before chromatographic analysis to prevent column blockage and increased backpressure, thus ensuring consistent instrument performance. To evaluate the effect of syringe filtration on analyte recovery, a standard solution containing 18 PAHs was prepared (individual concentrations are listed in Section 4.1). An aliquot (500 µL) of the prepared standard solution was transferred directly into a vial, while another 500 µL was passed through a PTFE syringe filter before being placed into a second vial. Both the unfiltered and filtered standard solutions were injected in triplicate and analyzed under identical chromatographic conditions. Chromatographic analyses (Figure S4, Supplementary Materials) showed similar responses between the unfiltered and filtered standards. These results suggest that the filtration step does not contribute to analyte retention. Therefore, this step can be safely incorporated into the sample preparation workflow to protect analytical instrumentation without compromising data integrity.

##### *Redissolving Solvent* 

The selection of the redissolution solvent was based on our previous work [24], in which acetonitrile, ultra-pure water, and acetonitrile–water mixtures (75:25, 50:50, and 25:75, *v*/*v*) were evaluated. The results obtained in the present study (single replicate) were consistent with those previously reported, confirming acetonitrile as the most suitable solvent for redissolving the extracted PAHs, particularly the high molecular weight compounds. However, the losses of lighter PAHs observed across all tested solvents indicate that these losses occurred prior to the redissolution step, suggesting that the solvent choice was not responsible for their low recoveries.

##### *Evaporation Step* 

The results from the complete SPE procedure (pre-treatment of the sample, SPE followed by elution and subsequent evaporation) presented in Section 4.2 showed losses of the lighter PAHs. However, it was not clear whether these losses were caused by incomplete elution with dichloromethane or by volatilization during the evaporation step. To determine if volatilization was responsible for the losses, the next study focused exclusively on the evaporation step. If the problem was not located in the evaporation step, then several solvents should be tested in the elution step.

A series of tests was conducted according to the workflow shown in Figure 5. As the study of this subsection focused directly on the evaporation step, the complete SPE procedure was not performed. Instead, a standard solution was prepared and used to evaluate the workflow. In line with the principles of Green Analytical Chemistry, this approach allowed the effects of evaporation to be isolated from the rest of the SPE procedure, while reducing the consumption of cartridges, solvents, and other materials. Moreover, a single replicate was used for each tested condition during this preliminary investigation. This approach also helped minimize solvent waste, material consumption, and analysis time. Once the step responsible for the observed losses was successfully identified and resolved, the complete procedure should be repeated using replicates to ensure statistical reliability and assess the reproducibility of the method.

Particular attention was given to four factors suspected of influencing analyte recovery: (i) the evaporation behavior of potential elution solvents (Figure 5, Procedures A and B), (ii) the evaporation process itself (Figure 5, Procedure C), (iii) the addition of a ‘keeper’ to various elution solvents prior to evaporation to minimize volatile analyte losses (Figure 5, Procedure D), and (iv) the evaporated volume (Figure 5, Procedures E and F).

Procedures A through F were tested sequentially. The process began with Procedure A, and at the end of the tested procedure, if analyte loss was observed, the next procedure (Procedure B) was evaluated. This step-by-step approach was continued until no loss of the most volatile PAHs was detected. Standard PAH solutions were prepared in each of the tested solvents according to the workflow shown in Figure 5.

The resulting peak areas from the tested procedures were compared with those of a reference standard prepared in acetonitrile. After identifying and addressing the critical factors responsible for analyte losses, the complete SPE procedure followed by HPLC analysis was performed to evaluate method recovery and confirm its suitability for PAH determination in water samples.

The tested conditions and obtained results are presented and discussed in the following subsections.

**Evaporation step: Elution Solvent(s)**

To isolate the evaporation step, a standard PAH solution was prepared in dichloromethane and underwent the workflow identified as Procedure A (Figure 5). The PAH recoveries obtained following Procedure A were compared with the results obtained using the complete SPE workflow (Figure 3 and Figure 4 and Figure S2). If analyte losses are observed during Procedure A, it can be attributed to the evaporation step. Conversely, if no losses are detected during Procedure A, it indicates that the losses occur prior to evaporation, most likely because the elution solvent fails to completely elute the target PAHs from the SPE cartridge.

Using Procedure A (Figure 5), considerable losses were recorded for the lightest PAH compounds, specifically 97.5% for Naph, 73.4% for Acen, 77.8% for Ace, and 49.1% for Flu. In the case of Phe and Ant, losses of 12.9% and 23.2% were observed. In contrast, from PAH7 to PAH18, only minor losses were observed, ranging from 0.701% for DB(a,l)P to 7.09% for Fln, respectively. A clear trend was observed: increasing the molecular weight of the target analytes improved retention. The consistency between these results and those obtained using the full procedure (Section 4.2) indicates that the loss of lighter PAHs occurs primarily during the evaporation step rather than during elution. These results are closely linked to the physicochemical properties of both the studied PAHs and the solvent dichloromethane. Based on the physicochemical properties summarized in Table S2 (Supplementary Materials), dichloromethane efficiently elutes PAHs owing to its favorable log Kow. However, its low boiling point (40 °C) and high vapor pressure (435 mm Hg) promote rapid solvent evaporation. Regarding the analytes, the lighter PAHs have higher vapor pressures than their heavier counterparts, making them more susceptible to volatilization. Due to the high volatility of both the solvent and lighter analytes, the rapid evaporation of dichloromethane promotes the co-evaporation of low molecular weight PAHs, which lack the stability to remain in the liquid phase.

The observed losses are consistent with the existing literature regarding the evaporation step. Osik et al. [57] demonstrated that solvent evaporation can result in the loss of volatile compounds with high vapor pressures. Similarly, Jakobsen et al. [58] observed that during pre-concentration with dichloromethane, volatile and semi-volatile compounds tend to co-evaporate with the solvent. In line with these findings, Fu and Zou [56] reported lower recoveries of lighter PAHs when the SPE eluate was concentrated under a flow of nitrogen.

Since procedure A confirmed considerable evaporative losses of lighter PAHs, procedure B was carried out following the workflow in Figure 5. This procedure evaluated whether alternative solvents with lower vapor pressures than dichloromethane could minimize such losses. The use of less volatile solvents was expected to improve analyte retention in the liquid phase, thereby reducing co-evaporation during the concentration step. Thus, the selected solvents included: *n*-hexane (non-polar); ethyl acetate and tetrahydrofuran (moderately polar); and methanol, isopropanol, and acetonitrile (polar). Additionally, the following mixtures were also tested: dichloromethane–*n*–hexane (1:1, *v*/*v*), tetrahydrofuran–isopropanol (1:1, *v*/*v*), and methanol–ethyl acetate (1:1, *v*/*v*). Detailed physicochemical properties of all solvents are provided in Table S2 (Supplementary Materials). The PAHs standard was prepared in each of the tested solvents or mixtures and procedure B was performed. The solvents demonstrating minimal PAH loss will be selected to evaluate the complete SPE procedure and assess their elution efficiencies.

Figure 6 shows the effect of each tested solvent on PAH recovery during evaporation, using the solvent dichloromethane (gray bars; solvent used in procedure A) as a reference point to evaluate the alternative elution solvents and mixtures (colored circles).

Across all tested solvents, percentage losses were highest for low molecular weight PAHs and decreased with increasing molecular size.

Naph was the most affected analyte, with losses ranging from 93.9% in tetrahydrofuran to 100% in isopropanol and methanol. This near-total loss is consistent with Naph vapor pressure (8.5 × 10^−2^ mm Hg), the highest among the studied PAHs. Similarly, other low molecular weight compounds, including Acen, Ace, and Flu, had substantial losses, generally exceeding 50% across most solvents. Notable exceptions occurred with tetrahydrofuran and the tetrahydrofuran–isopropanol (1:1, *v*/*v*) mixture, where losses were lower, ranging from 37.5% (Flu) to 61.9% (Ace). Medium-molecular-weight PAHs (4 rings), such as Phe, Ant, Fln, and Pyr, showed intermediate losses ranging from 5.21% to 55.2%. In contrast, high molecular weight PAHs (5–6 rings) showed minimal to negligible losses.

The assumption that dichloromethane would yield the highest losses for the studied PAHs owing to its high vapor pressure was not supported by the results. In fact, dichloromethane had equal or lower losses compared to most of the tested solvents. The only exceptions were observed for Acen, Ace, and Flu, for which tetrahydrofuran and the mixture tetrahydrofuran–isopropanol showed improved recovery. Compared to dichloromethane, losses for these analytes were reduced by 27.2%, 27.7%, and 11.6%, respectively, when using tetrahydrofuran, and by 14.2%, 16.0%, and 9.0% when using the mixture tetrahydrofuran–isopropanol. Furthermore, when comparing tetrahydrofuran (which yielded the lowest PAH loss) with other alternative solvents, the differences for Acen, Ace, and Flu were even more pronounced, with losses approximately 20% to nearly 50% lower than those observed for the remaining tested solvents. The highest differences were observed, with losses nearly 50% lower (tetrahydrofuran vs. *n*-hexane) for Acen, nearly 40% lower (tetrahydrofuran vs. ethyl acetate or isopropanol) for Ace, and nearly 50% lower (tetrahydrofuran vs. ethyl acetate) for Flu.

To investigate the causes of these trends, the physicochemical properties of both the analytes and solvents were analyzed. Figure S5 (Supplementary Materials) provides an overview of four key physicochemical properties, where the *x*-axis represents log K_ow_, the *y*-axis represents molecular weight, bubble size indicates vapor pressure (larger diameters corresponding to higher vapor pressure), and the color scale represents boiling point, as indicated in the legend of each plot for the PAH compounds (Figure S5A, Supplementary Materials) and for the solvents (Figure S5B, Supplementary Materials). In Figure S5A (Supplementary Materials), the large spheres representing low molecular weight PAHs (such as Naph, Acen, Ace, and Flu) highlight their high vapor pressure and natural susceptibility to co-evaporation. Correspondingly, Figure S5B (Supplementary Materials) illustrates the higher vapor pressure of dichloromethane relative to the alternative solvents tested.

The solvents, organized by descending order of vapor pressure, followed the sequence: dichloromethane > *n*-hexane ≈ tetrahydrofuran > methanol > ethyl acetate > acetonitrile > isopropanol (Table S2, Supplementary Materials). This hierarchy was inversely reflected in the time required for evaporation to dryness, ranging from the shortest (dichloromethane) to the longest (isopropanol). Interestingly, dichloromethane has a higher vapor pressure than the other evaluated solvents. However, lower overall PAH losses were observed for most analytes when dichloromethane was used. In contrast, tetrahydrofuran and the tetrahydrofuran–isopropanol mixture demonstrated a specific protective effect for Ace, Acen, and Flu. The initial hypothesis, that less volatile solvents would minimize PAH loss, was not supported by the data. Instead, when using less volatile solvents, the analytes remained under evaporation conditions for an extended duration, which increased cumulative volatilization losses over time (Figure 6 and Figure S5 (Supplementary Materials)).

Since none of the solvents tested in procedures A and B prevented losses for the lighter PAHs, additional studies are required.

**Evaporation step: Solvent evaporation process**

To evaluate whether alternative concentration methods could reduce PAH losses relative to the nitrogen stream evaporation (Procedure A), Procedure C (Figure 5) was performed. This approach compared three evaporation methods: evaporation under a nitrogen stream (Procedure A, Figure 5), rotary evaporation (Procedure C1, Figure 5), and evaporation at room temperature (Procedure C2, Figure 5). The resulting extracts were analyzed alongside a reference standard solution, and the obtained data are presented in Figure 7.

The results presented in the elution solvents subsection of the evaporation step study indicate that PAH losses are strongly influenced by both compound and solvent volatility. Furthermore, the results shown in Figure 7 underscore the relevant impact of the evaporation method, demonstrating that this step is critical in the analytical workflow. The largest losses in Figure 7 occurred for the low molecular weight PAHs, where the loss was nearly total. Specifically, evaporation at room temperature proved to be the least effective approach for these compounds, resulting in a 100% loss of Naph, Acen, and Ace. Although slightly lower losses were recorded when using a nitrogen stream or rotary evaporator, these compounds remained highly susceptible to volatilization regardless of the method employed.

Substantial losses were also observed for intermediate PAHs, including Flu, Phe, and Ant, across all techniques. Evaporation at room temperature consistently resulted in higher losses than nitrogen and rotary evaporation, confirming that prolonged exposure time enhances volatilization. Losses ranged from 12.9% (Phe) and49.1% (Flu) using nitrogen, between 29.1% (Phe) and 46.2% (Flu) using a rotary evaporator, and between 50.4 (Phe) and 98.5% (Flu) at room temperature.

In contrast, heavier PAHs generally exhibited lower losses, although the results varied depending on the evaporation method used. Rotary evaporation tended to obtain higher losses than nitrogen evaporation for several high molecular weight compounds, with losses exceeding 10% for Flu, Pyr, DB[a,l]P, and InP. This suggests that reduced pressure and increased solvent evaporation rates may promote analyte loss, even for less volatile compounds. Room-temperature evaporation yielded variable results. For the heavier PAHs, the observed losses were comparable to, or even lower than, those obtained using the other evaporation techniques.

In summary, although the nitrogen stream yielded the best overall results, none of the tested methods successfully achieved minimum losses across the entire range of studied PAH compounds. These findings align with the observations of Dąbrowski (2016) [59], who reported that more volatile compounds are prone to loss regardless of the process used, ranging from conventional techniques (rotary evaporation and gentle nitrogen streams) to more advanced methods (centrifugal force or gas-flow systems).

**Evaporation step: Keeper Addition Prior to Solvent Evaporation**

While volume reduction is essential to achieve the low final volumes necessary for high-sensitivity detection, the loss of target analytes during this stage remains an important challenge that can compromise recovery rates. To prevent losses during evaporation, many studies [36,59,60,61,62,63,64,65,66,67,68,69,70,71,72,73,74,75,76,77,78,79] have added a second, less volatile solvent, known as a “keeper”, to the primary solvent. One of the earliest studies on this topic was conducted by Thornburg [80] in 1963, who suggested that additives such as ethylene glycol or white oil could stabilize pesticides in sample extracts during evaporation.

Because keeper solvents have high boiling points, they do not evaporate as easily as the primary solvent. They remain in the vial to “trap” the target analytes, preventing them from escaping as the primary solvent evaporates [59]. For PAHs (see Table S3 in the Supplementary Materials), researchers typically use dichloromethane [69,70], *n*-hexane [69,73,74] or a mixture of dichloromethane and *n*-hexane [60,61,62,64,65,67,68,81,82,83] as primary solvents. Common keepers include toluene [61,62,67,69], *N,N*-dimethylformamide [72,75,81,82,83], and long-chain alkanes (such as dodecane [76,77,78,79] and nonane [60,73]). Most protocols involve either (i) adding the keeper before starting the evaporation or (ii) performing a partial evaporation, adding the keeper, and then continuing the evaporation [60,61,62,64,65,67,68,69,71,72,74,75,76,77,78,79,81,82,83]. A gentle stream of nitrogen gas is commonly used during evaporation [61,64,65,69,72,74,75,81,82,83]. However, other studies used more advanced evaporation processes [68,71,73,77]. Keeper volumes are typically small, often ranging from 5.0 to 2000 μL. While many studies complete the process in one step [60,61,62,64,65,68,69,71,72,73,74,75,77,78,81,82,83], a two-step sequence is often used for large initial volumes (50–300 mL) [67,70,76,79]. In this approach, a rotary evaporator is first used to rapidly reduce the volume to a few milliliters, and a gentle nitrogen stream is then used to reach the precise target volume. This dual strategy maximizes the sample concentration factor while ensuring strict control of the volume to be evaporated.

The present study evaluated different combinations of primary and secondary (keeper) solvents to assess their effectiveness in preventing PAH losses during the evaporation step. These experiments were performed following Procedure D (Figure 5). In total, 143 combinations were evaluated (Table S4, Supplementary Material), comprising eight primary solvents and seven keeper solvents. The primary solvents included dichloromethane, *n*-hexane, and several mixtures: dichloromethane:cyclohexane (1:1, *v*/*v*), dichloromethane:*n*-hexane (1:1, *v*/*v*), dichloromethane:acetone (1:1, *v*/*v*), dichloromethane:*n*-hexane:acetone: (1:1:1, *v*/*v*/*v*), dichloromethane:pentane (1:1, *v*/*v*), and dichloromethane:cyclohexane:acetone (1:1:1, *v*/*v*/*v*). The keeper solvents tested were *N,N*-dimethylformamide, hexanol, isopropanol, isooctane, methanol, octanol, and toluene. The selection of primary and keeper solvents, as well as the volumes of the keeper solvents used, was based on the study performed by Dąbrowski [59].

It must be highlighted that dichloromethane was selected as the primary solvent for nearly all experiments. Dichloromethane was identified in an application note as the optimal solvent for eluting PAHs from the Strata PAH sorbent [41,43]. By ensuring that the primary solvent already guarantees robust analyte recovery from the solid phase, the identification of a secondary solvent that reduces volatilization losses during the evaporation step effectively validates the entire analytical workflow. The obtained results of the tested experiments following procedure D described in Figure 5 are presented in Table S5A (Supplementary Materials). The results are discussed in the following paragraphs according to the tests performed.

- Screening of Primary Solvents (dichloromethane and *n*-hexane) and Keepers (methanol, isooctane, and isopropanol)

For the first tests, dichloromethane was used as the primary solvent, with methanol (Test 7), isooctane (Test 22), and isopropanol (Test 37) added as keepers at 10% (*v*/*v*). The experiments were then repeated using *n*-hexane as the primary solvent with the same keepers, methanol (Test 56), isooctane (Test 71), and isopropanol (Test 86), at the same concentration (Table S4, Supplementary Materials). The data in Table S5A (Supplementary Materials) reveal a critical challenge during the concentration step. This issue is primarily due to the marginal protective effect of the keepers (methanol, isooctane, and isopropanol), which failed to prevent substantial losses of volatile PAHs despite their lower vapor pressures compared with the tested primary solvents (dichloromethane and *n*-hexane).

Regarding the dichloromethane trials, for instance, the addition of 10% methanol (Test 7, Table S4 (Supplementary Materials)) failed to improve the recovery of volatile PAHs, yielding results nearly identical to those of the control (dichloromethane without a keeper). In contrast, using isooctane (Test 22) as a keeper showed minimal reduction in PAH loss: 3.3% for Naph, 10.2% for Acen, 13.1% for Ace, and 11.5% for Flu. Isopropanol (Test 37, Table S4 (Supplementary Materials)) provided no benefit for Naph and only minimal reductions for the remaining analytes (5.54%, 11.4%, and 7.76% for Acen, Ace, and Flu). While isooctane emerged as the most effective keeper for the primary dichloromethane solvent, the improvements when compared without using a keeper for the volatile PAH compounds remained insufficient. The efficiency of these keepers was further compromised in the *n*-hexane experiments. None of the tested keepers substantially reduced the loss of Naph. While all three keepers performed similarly for Flu, reducing losses by approximately 8.1% to 10.5%, isopropanol was the only keeper to demonstrate a clear advantage for Acen and Ace, reducing losses by 16.8% and 13.2%, respectively, compared to the *n*-hexane control.

In these initial trials, the choice of primary solvent was more influential than the selection of keepers. Although dichloromethane has a higher vapor pressure than *n*-hexane, the losses of lighter PAHs were higher when *n*-hexane was used. This indicates that the longer evaporation time required for *n*-hexane increases the duration of analyte exposure to the process, thereby increasing the loss of volatile compounds through co-evaporation. Ultimately, despite minor improvements with selected keepers, none of the tested conditions sufficiently prevented substantial analyte loss.

b. Effect of Keeper Concentration (1 to 50% *v*/*v*: Methanol, Isooctane, and Isopropanol)

After these initial trials, the next phase of the study comprised 15 tests for the three tested keepers, methanol, isooctane, and isopropanol, with concentrations ranging from 1% to 50% (*v*/*v*) (Tests 1 to 94; Table S4, Supplementary Materials). The objective was to analyze the behavior of the target PAHs across a wide concentration range of the keeper concentration in both primary solvents (dichloromethane and *n*-hexane).

The results demonstrated that varying the keeper concentration between 1% and 50% did not result in a substantial improvement in PAH recovery. This implies that the keeper’s primary function, intended to retain analytes within a high-boiling-point liquid, was insufficient to protect the target PAHs during the evaporation process. Consequently, Naph experienced near-total loss (reaching 100% in most trials) regardless of the primary solvent, keeper solvent, or keeper concentration. While losses for Acen and Ace ranged from 60% to 90%, and Flu from 40% to 60% (Table S5A, Supplementary Materials). Nevertheless, the losses for these more volatile PAH compounds remain unacceptably high for standard analytical protocols.

c. Expanded Evaluation of Primary Solvents and Keepers

The study was expanded, and an additional 49 experiments (Tests 7, 22, 37, 46–49, 56, 71, 86, 95–119, 123–143 (Table S4, Supplementary Materials)) were performed using eight primary solvents combined with seven different keepers.

Naph was identified as the most critical compound owing to its high volatility, which led to substantial losses across nearly all tested conditions. In most experiments, losses exceeded 90%, demonstrating that the evaluated keepers were generally ineffective for such highly volatile PAHs. Losses below 90% for Naph were limited to specific combinations: isooctane in dichloromethane–pentane (1:1, *v*/*v*); isopropanol in dichloromethane–acetone (1:1, *v*/*v*); octanol in dichloromethane–*n*–hexane (1:1, *v*/*v*); hexanol in dichloromethane; and *N,N*-dimethylformamide in dichloromethane–*n*-hexane (1:1, *v*/*v*) (Table S5A, Supplementary Materials). The superior retention achieved with isooctane and isopropanol (80% loss), octanol (64.9% loss), hexanol (55.5% loss), and *N,N*-dimethylformamide (34.1% loss) is attributed to their high boiling points and lower volatility (Table S2 and Figure S5C, Supplementary Materials). These keepers persist in the liquid phase longer than the primary solvents, effectively trapping lighter PAHs during the evaporation step.

Toluene consistently resulted in near-complete loss of volatile PAHs and substantial losses for all remaining compounds, regardless of the primary solvent utilized. This outcome confirms its poor performance as a keeper, as it fails to effectively stabilize the analytes during evaporation.

Octanol demonstrated moderate effectiveness specifically within the dichloromethane–*n*–hexane (1:1, *v*/*v*) system, yielding losses of 64.9% for Naph, 16.1% for Acen, and 9.35% for Ace. However, under most other conditions, losses remained comparatively high.

Hexanol provided improved retention when dichloromethane was used as the primary solvent, reducing losses across all analytes (55.5% for Naph, 36.9% for Acen, 29.0% for Ace and 8.75% for Flu). By contrast, the addition of hexanol to the primary solvent *n*–hexane increased analyte losses, highlighting the negative impact of less polar solvent mixtures on analyte stability.

By contrast, isopropanol, isooctane, and methanol consistently exhibited high loss rates across all primary solvents (>85–90% for Naph, 63.2–94.5% for Acen, 60.4–95.5% for Ace, and 37.6–90% for Flu), suggesting their limited effectiveness as keeper solvents.

Among the tested keepers, *N,N*-dimethylformamide showed highly variable behavior towards the studied compounds, with results ranging from losses to apparent gains, depending on the solvent system. For Naph, losses fluctuated between 34.1% and 98.9%, while gains between 17.2% and 88.1% were recorded (Table S5A, Supplementary Materials). This wide variability suggests that *N,N*-dimethylformamide does not act solely as a keeper, but substantially modifies the analytical response. The most stable outcomes were observed using dichloromethane–*n*–hexane (1:1, *v*/*v*) with a 34.1% loss and dichloromethane–*n*–hexane–acetone (1:1:1, *v*/*v*/*v*) with a 17.2% gain.

For high molecular weight PAHs, lower losses were generally observed compared to their lighter counterparts, with the performance being strongly influenced by the choice of primary solvent. Dichloromethane, whether used alone or in mixtures, consistently provided the best results across a wide range of keepers, including methanol, isooctane, isopropanol, hexanol, and *N,N*-dimethylformamide. Similarly, *n*–hexane exhibited acceptable performance, particularly when combined with methanol, isooctane, isopropanol, or *N,N*-dimethylformamide. However, the losses observed with *n*–hexane were slightly higher than those recorded for the dichloromethane-based systems.

Among the solvent mixtures, dichloromethane-based systems yielded the most favorable outcomes. Better retention was noted in binary mixtures, such as dichloromethane–acetone and dichloromethane–pentane (1:1, *v*/*v*), as well as ternary mixtures, such as dichloromethane–*n*–hexane–acetone and dichloromethane–cyclohexane–acetone (1:1:1, *v*/*v*/*v*), particularly when *N,N*-dimethylformamide was used as the keeper. Other effective combinations included dichloromethane–cyclohexane with hexanol and dichloromethane –*n*–hexane (1:1, *v*/*v*) with toluene, although the latter demonstrated higher losses.

The role of acetone in the evaluated solvent mixtures was of particular significance. Although acetone is highly volatile, its inclusion in binary and ternary mixtures reduced analyte losses under certain conditions. To further investigate this effect, additional experiments (Tests 116, 120, 121, and 122; Table S4, Supplementary Materials) were performed by decreasing the acetone fraction in the ternary combination. Specifically, the dichloromethane–*n*–hexane–acetone combination was evaluated in proportions of 1:1:1, 1.2:1.2:0.6, 1.35:1.35:0.3, and 1.43:1.43:0.15 (*v*/*v*/*v*) using *N,N*-dimethylformamide as a keeper, which proved to be the most effective among all the studied keepers. The results (Figure S6, Supplementary Materials) indicated that lowering the acetone fraction directly influenced the recovery of the lighter PAHs. Higher proportions of acetone led to signal enhancement (apparent gains), whereas decreasing the acetone fraction resulted in higher volatilization losses (up to 19.3%). For Phe, gains were observed under all evaluated conditions, but decreased as the acetone fraction was reduced.

Among all tested conditions, the results with losses below 90% for the most volatile PAHs are summarized in Table S5B (Supplementary Materials). Key observations are recorded in Table S5B, and the main conclusions are presented in the following paragraphs.

Across most of the evaluated conditions (e.g., Tests 122, 102, 47, 105, 46, 131, and 108), PAH losses generally decreased with increasing molecular weight, following the trend Naph > Acen > Ace > Flu > Phe. The addition of high-boiling-point keepers, such as *N,N*-dimethylformamide, hexanol, and octanol, resulted in relatively low Naph losses under some conditions, ranging from 0.8% to 34.1% in Tests 102, 120, 121, and 122 (Table S5B, Supplementary Materials). However, substantial Naph losses were still observed under other conditions, reaching 55.5–78.1% in Tests 47, 105, and 46, while gains for Naph were observed in Tests 109, 116, and 133. The use of isooctane and isopropanol as keepers was less effective in preventing the volatilization of the most volatile PAHs, with losses ranging from 41.0% to 86.7% in Tests 131 and 108. Furthermore, the use of high-boiling-point alcohols, such as hexanol and octanol, resulted in viscous/oily, non-volatile residues after solvent evaporation, which may present practical limitations for subsequent HPLC analysis, including potential system contamination and adverse effects on analytical column performance. These results indicate that, although some keeper conditions reduced the loss of volatile PAHs, no single keeper/solvent combination provided satisfactory performance for all target PAHs while simultaneously avoiding potential limitations for subsequent chromatographic analysis.

The findings highlight a complex relationship between the polarity and volatility of the primary solvent, keeper, and analyte. Dichloromethane emerged as the most effective primary solvent, whether used independently or within a mixture. The high performance of *N,N*-dimethylformamide, particularly within the ternary system, underscores the need for high-boiling and low-volatility keepers when targeting volatile PAHs. However, while this specific combination minimizes analyte loss, the significant signal enhancement associated with *N,N*-dimethylformamide requires careful evaluation to ensure that the accuracy of the final quantification is not compromised. The effectiveness of *N,N*-dimethylformamide as a keeper was also confirmed in the studies of Kucuksezgin et al. [81], Telli-Karakoç et al. [82], and Tolun et al. [83], in which dichloromethane–*n*–hexane (1:1, *v*/*v*) was used as the primary solvent mixture.

The results obtained showed that no single combination provided satisfactory retention of all PAHs without significant losses. In addition, the risk of signal enhancement could compromise the accuracy of quantification, while the use of certain solvents may negatively affect the HPLC system and the performance and lifetime of the analytical column. Consequently, further optimization was required to balance the prevention of volatile analyte losses with the need to minimize potential adverse effects on the analytical system and column. These considerations motivated the evaluation of an additional step in the optimization workflow, as presented in Figure 5.

**Evaporation step: Volume evaporated**

Regarding the evaporation process, Ferreira et al. [84] previously reported that, depending on analyte properties, the solvent system, and the specific pre-concentration method employed, volatile compounds can be lost from 0% to 100% during the concentration step. Notably, these authors observed that analyte loss increases as the final volume decreases. To address this issue, two evaporation strategies were evaluated using procedures E and F (Figure 5): concentration of an initial 6 mL of dichloromethane to a final volume of 25 µL (Procedure E, Figure 5), and a two-stage approach involving partial evaporation of 6 mL of dichloromethane to 1 mL, followed by dilution with 3 mL of acetonitrile and subsequent re-evaporation to a final volume of 250 µL (Procedure F, Figure 5). To ensure chromatographic compatibility and prevent interference from high-boiling residues, the use of a keeper was avoided.

To evaluate the effect of the final volume on analyte losses, the results obtained from Procedure E (concentrated to a final volume of 25 µL) were compared with those obtained from Procedure A (evaporated to dryness) and presented in Figure 8A. Improvements were observed when the standards prepared in dichloromethane were evaporated to a final volume of 25 µL rather than being evaporated to dryness. Under these conditions, PAH losses were kept below 11% for all compounds, ranging from 3.81% for B[ghi]P to 10.8% for Acen (*n* = 3).

To further assess the applicability of this procedure, the complete SPE (sample pre-treatment using 25% acetonitrile (25 mL) and 75% drinking water (75 mL), SPE using STRATA-PAH cartridge, elution with dichloromethane, controlled evaporation using nitrogen to a volume of 25 µL, adjustment to a final volume of 250 µL with acetonitrile, filtration through a PTFE syringe filter and HPLC analysis) was evaluated in terms of recovery. Based on 15 replicates (*n* = 15), recoveries (average) of the 18 studied PAHs ranged from 51.7% (Naph) to 84.0% (Fln and Pyr), as illustrated in Figure 8B. However, despite the improved recoveries, the application of procedure E (Figure 5) resulted in considerable variability. Relative standard deviations (% RSD) ranged from 16.6% (Flu) to 40.3% (Naph), indicating limited reproducibility. This high variability was observed across all studied PAHs. For the more volatile analytes, recoveries ranged from 15.7 to 78.2% for Naph, 22.4–80.9% for Acen, 25.9–82.6% for Ace, and 26.7–86.0% for Flu. Although heavier PAHs generally showed higher recoveries, they also had high variation between the recoveries, from approximately 29.2% to 48.4% to over 89.3% to 111%. These results highlight that, although avoiding complete dryness reduces analyte loss, the evaporation step remains a critical source of variability, substantially affecting method precision.

Following the results obtained in Procedure E, a two-stage evaporation approach (Procedure F, Figure 5) was evaluated and a two-stage approach involving partial evaporation with nitrogen, followed by dilution using acetonitrile, and subsequent re-evaporation with nitrogen to a final volume of 250 µL was tested. Acetonitrile was added as a second solvent during the evaporation process because of its lower vapor pressure and higher boiling point compared with dichloromethane. In addition, acetonitrile was selected because it is the organic eluent of the chromatographic mobile phase, eliminating the need for a subsequent solvent exchange before analysis. By introducing a solvent that moderates the evaporation rate, acetonitrile acts as a ‘keeper’, ensuring that target analytes remain in the liquid phase longer and effectively reduces volatile losses. Furthermore, the final volume in this procedure was set to 250 µL—a tenfold increase compared to the 25 µL used in Procedure E—specifically to further minimize the loss of the most volatile target analytes.

The standard solution prepared in dichloromethane was subjected to Procedure F, and the results were comparable to those obtained with Procedure E (Figure 8A), with PAH losses remaining below 10%. Based on these satisfactory results, the full SPE methodology was subsequently performed to determine analyte recovery and method reproducibility. Ten spiking levels were evaluated, and the concentrations used are listed in Table S6 (Supplementary Materials). The resulting recoveries for all fortification levels are listed in Table S7 (Supplementary Materials).

High recoveries were achieved across all fortification levels. The results were reproducible, and improved recovery rates were observed for all studied PAHs. The minimum, maximum, and average recoveries for each level were as follows: (i) Spiking I: 78.9%, 107%, 94.9%, (ii) Spiking II: 78.9%, 111%, 96.5%; (iii) Spiking III: 80.0%, 112%, 97.8%; (iv) Spiking IV: 80.5%, 110%, 97.7%; (v) Spiking V: 80.2%, 108%, 97.0%; (vi) Spiking VI: 80.9%, 108%, 99.7%; (vii) Spiking VII: 80.6%, 108%, 96.6%, (viii) Spiking VIII: 78.5%, 107%, 96.7%; (ix) Spiking IX: 79.2%, 105%, 96.2%, and (x) Spiking X: 84.9%, 111%, 96.8%. The average recoveries across all spiking levels ranged from 80.3% to 106%, with RSDs ranging from 1.7% to 6.0% (Table S7, Supplementary Materials). For Procedure F, final recoveries were within this range, with an overall mean recovery of 97.0% (95% confidence interval: 94.6–99.4%). Individual 95% confidence intervals ranged from ±0.90% for Flu to ±3.70% for DB[ah]A. To evaluate the improvement in precision between Procedure E (evaporation to 25 µL, *n* = 15) and Procedure F (two-stage partial evaporation with 3 mL acetonitrile to 250 µL, *n* = 10), two-sample F-tests comparing analyte variances (S_E_^2^/S_F_^2^) were performed. Procedure E exhibited high variability, with RSD ranging from 16.6% to 40.3% (mean RSD = 22.3%). In contrast, Procedure F yielded exceptional precision, with RSD values ranging from 1.66% to 6.04% (mean RSD = 3.48%). Calculated F-ratios ranged from 6.67 to 151.88, exceeding the critical value (14, 9 = 3.01) for all PAHs (*p* < 0.005). These results demonstrate that Procedure F provides significantly lower variability than Procedure E.

As detailed in Table S3, the traditional literature keeper strategies relying on long-chain alcohols (e.g., hexanol, octanol), high-boiling alkanes (dodecane, nonane), or polar additives (*N,N*-Dimethylformamide) improve volatile 2–3-ring PAH retention compared to keeperless drying, but introduce significant analytical drawbacks. These include non-volatile viscous residues that accumulate on HPLC C18 stationary phases (alcohols/lipids) or severe optical response modification artifacts. In contrast, Procedure F replaces permanent keepers with a controlled two-stage partial evaporation using 3 mL of acetonitrile as a transient modifier. Compared with the results reported in Table S3, Procedure F provides comparable or superior retention of the volatile PAHs, with recoveries of 80.3–88.3% for Naph, Acen, Ace, and Flu and an overall mean recovery of 97.0%. In addition, Procedure F demonstrates excellent repeatability (RSD < 6.0%) while avoiding the formation of non-volatile residues, detector signal distortion, and additional solvent-exchange steps.

The high recovery rates and low RSD values demonstrate that procedure F (Figure 5) is a robust and highly reproducible method for the analysis of 18 target PAHs in water samples. This procedure optimizes evaporation process by introducing 3 mL of acetonitrile as a high-boiling point keeper and concentrating the extract to a final volume of 250 µL. In contrast, Tolun et al. [83] and Kucuksezgin et al. [81] target final volume of 1 mL, and Kraleva et al. [85] report 200 µL. While preventing analyte loss, our method allows also a 300x concentration factor.

### 4.4. Influence of Temperature on the Evaporation Step

Considering the wide range of physicochemical properties of the 18 target PAHs, specifically their varying vapor pressures and volatilities (Table S2 and Figure S5, Supplementary Materials), all evaporation steps were strictly conducted at ambient temperature. No thermal assistance was employed, as maintaining environmental temperatures is critical for preserving the integrity of the most volatile compounds. This methodological choice is strongly supported by the findings of Kraleva et al. [85], who demonstrated that heating the eluates during concentration leads to a substantial loss of low molecular weight, smaller-ring PAHs.

### 4.5. Evaluation of Vacuum Pressure and Post-Loading Steps (Washing/Drying)

The final optimization stage focused on evaluating the vacuum pressure during SPE, alongside the washing and drying steps. As these steps were screening tests, a single replicate for each tested condition was performed to minimize the consumption of SPE cartridges, solvents, materials, and time, in accordance with the principles of Green Analytical Chemistry. The vacuum pressure must be controlled to allow sufficient interaction between the analytes and sorbent, thereby preventing breakthrough. The washing step was assessed because of its critical role in removing matrix interferences present in water samples. Finally, the drying step must be carefully evaluated; while the complete removal of residual water is essential for efficient elution with dichloromethane, special care must be taken because excessive drying times can lead to the loss of analytes.

Vacuum pressures of 5 and 10 mmHg were evaluated. For the washing step, the ‘no-wash’ condition was compared with both a water wash [56] and a water:methanol (1:1, *v*/*v*) mixture wash [41]. Finally, the ‘no-drying’ condition was compared against drying intervals of 15 s [41], 1 min, and 5 min. Figure S7 (Supplementary Materials) presents the recovery of the studied PAHs in the different conditions. The reference condition was performed by using a vacuum pressure of 5 mmHg, with no washing step and no drying step in the SPE procedure. Experimental test conditions (single parameter modifications evaluated while maintaining all other parameters at reference baseline values) were: (i) vacuum pressure at 10 mmHg; (ii) cartridge washing with 100% ultrapure water; (iii) cartridge washing with ultrapure water:methanol (1:1, *v*/*v*); (iv) cartridge drying under vacuum for 15 s; (v) cartridge drying under vacuum for 1 min; and (vi) cartridge drying under vacuum for 5 min.

Differences in recoveries and analyte losses were observed across the different experimental conditions. When comparing pump pressures of 5 and 10 mmHg (conditions: without washing and without drying steps), using higher pressure resulted in substantially higher losses, particularly for PAH 9 (B[a]A) to PAH 18 (InP). Losses ranged from 30% to nearly 50%, with DB[ah]A showing the highest loss with 46.8%. For the lighter PAHs, losses were lower, ranging from 16.2% (Acen) to 26.3% (Naph). The lowest loss was observed for Phe (5.18%) when using a higher vacuum pressure.

Losses were also observed when water or a water–methanol (1:1, *v*/*v*) solution was compared to the ‘no-wash’ condition (conditions: vacuum pressure of 5 mmHg and without drying step). For most PAHs, higher recovery differences were observed when the water–methanol (1:1, *v*/*v*) mixture was used. Exceptions were B[a]A, Chry, B[k]Ft, B[ghi]P, and InP, for which higher differences were observed when water alone was used for the washing step. Notably, Chry yielded a higher recovery with the water–methanol (1:1, *v*/*v*) wash than under the reference (no-wash) condition. The largest recovery differences were observed for Phe, reaching 41.4% and 49.5% when using water and the water–methanol (1:1, *v*/*v*) solution, respectively.

The drying step (conditions: vacuum pressure of 5 mmHg and without washing step) also had a considerable impact on analyte recovery. The recovery results obtained under the ‘no-drying’ condition were compared with the recovery when 15 s, 1 min, and 5 min were used in the drying step. Increasing drying time to 15 s, 1 min, or 5 min led to higher losses for lighter PAHs, indicating their susceptibility to volatilization. In contrast, heavier PAHs showed a different trend, with higher losses observed at shorter drying times (15 s). Maximum losses reached 29.0% at 15 s, 18.5% at 1 min, and 20.2% at 5 min.

Based on these results, the optimal conditions were selected as a vacuum pressure of 5 mm Hg, with no washing or drying steps during SPE, as this minimized losses for both volatile and heavier PAHs. However, omitting the drying step may result in residual water being co-eluted with dichloromethane. Therefore, careful phase separation is required prior to evaporation to remove any remaining aqueous phase. Our findings align with those of Kraleva et al. [85], who noted that higher vacuum pressures during extraction could lead to substantial PAH losses. This supports our decision to use a lower, more controlled pressure to enhance analyte retention on the sorbent bed.

### 4.6. Addition of Fat to Retain Compounds During Evaporation

Last test involved the addition of a lipid keeper to the dichloromethane eluate prior to evaporation to dryness. The objective was to retain volatile compounds by providing a non-volatile matrix, thereby preventing analyte loss during the final concentration phase. This approach was evaluated as a simplified alternative to the two-stage evaporation process described previously in Procedure F (Figure 5).

Previous studies by the authors on PAH extraction from complex matrices, such as fish tissue [86,87], have demonstrated that the lipids present in the sample retain volatile compounds during nitrogen evaporation to dryness. The hydrophobic interactions between PAHs and lipids create a protective phase that minimizes volatilization. Similarly, adding a lipid keeper to water sample eluates could protect volatile PAHs. However, some issues must be considered: (i) added lipids may introduce chromatographic interferences and accelerate column contamination; (ii) an additional cleanup step would be required for the post-evaporation extract to remove the fat; and (iii) the lipid source itself may introduce PAH contamination. Therefore, the area of the blank and the area of the fortified samples must be subtracted to ensure analytical accuracy.

An experiment (*n* = 1) was conducted by adding a drop of olive oil (25 µL) as a keeper lipid to 6 mL of dichloromethane containing the target PAHs (black bar in Figure S8, Supplementary Materials). The solution was evaporated to dryness under a gentle stream of nitrogen, and the resulting residue was redissolved in acetonitrile and the extract was injected. To evaluate the efficacy of this method, results were compared against three alternative evaporation protocols already tested in the present study: (i) direct evaporation: 6 mL of dichloromethane evaporated with nitrogen to complete dryness without a keeper (red circle, Figure S8, Supplementary Materials, Procedure A), (ii) Procedure E (Figure 5): 6 mL of dichloromethane evaporated with nitrogen to a final volume of 25 µL (blue bar Figure S8, Supplementary Materials), and (iii) Procedure F (Figure 5): 6 mL of dichloromethane evaporated with nitrogen in a two-stage evaporation and reach a final volume of 250 µL (green bar Figure S8, Supplementary Materials).

Comparing the losses obtained in procedure A with those observed when lipid keeper was added, the obtained data clearly demonstrate the efficacy of using a lipid keeper to minimize analyte loss during evaporation, with recorded losses ranging from 1.28% for Phe to 22.3% for Acen. However, the lipid keeper generally resulted in higher losses for most PAHs than those obtained using partial evaporation procedures (Procedures E and F; Figure 5). While the lipid keeper showed potential for specific compounds, Procedure F provided the most consistent results across all 18 target PAHs, while eliminating the analytical risks associated with chromatographic interference and the need for additional cleanup steps.

### 4.7. Final Procedure for Extraction of PAHs in Water Samples Using Solid-Phase Extraction

The extraction of PAHs from water samples was performed using an SPE system with a twelve-position vacuum manifold. A constant vacuum pressure of 5 mmHg was maintained throughout the procedure. Prior to extraction, a 100 mL sample was prepared by mixing 75 mL of the water sample with 25 mL of acetonitrile (75:25, *v*/*v*). The strata-PAH SPE cartridges (1.5 g, 6 mL) were conditioned with 20 mL of dichloromethane, followed by 20 mL of methanol, then equilibrated with 20 mL of ultrapure water. Care was taken to ensure that the sorbent did not dry out between solvents. The sample was then loaded onto the cartridge. No washing or drying steps were performed post-loading to avoid the loss of PAH analytes. Next, the target analytes were eluted from the sorbent using 6 mL of dichloromethane. Eliminating the drying step resulted in a two-phase system (aqueous and organic) with a much smaller volume of the aqueous phase compared to the organic phase. The upper aqueous phase was carefully separated and transferred to a second tube. Care must be taken to ensure that the second tube contains only the aqueous phase; otherwise, any dichloromethane that has been accidentally transferred must be returned to the first tube. The organic eluate was subsequently concentrated to a volume of 1 mL under a gentle stream of nitrogen. Then, 3 mL of acetonitrile was added, and evaporation continued until a final extract volume of 250 µL was obtained. Finally, the concentrated sample extract was filtered through a PTFE syringe filter (0.22 µm, 13 mm) prior to injection into the HPLC-PAD/FLD for PAH analysis. This procedure is illustrated in Figure 9.

### 4.8. Validation

Method validation was performed by evaluating linearity, sensitivity, accuracy, and precision. Linearity was assessed using solvent-based and matrix-matched calibration curves for surface water (ocean and river) and wastewater (WWTP influent and effluent) matrices. Method sensitivity was evaluated based on the slopes of the solvent-based and matrix-matched calibration curves. The LOD and LOQ obtained from solvent-based calibration curves, as well as the MDL and MQL obtained from matrix-matched calibration curves, were used to characterize the detection and quantification capabilities of the analytical method. Using the calibration curve prepared in the solvent and the calibration curves prepared in the matrix, ME were determined. Accuracy was evaluated by calculating the percentage recovery across DW, surface water, and wastewater matrices, while method precision was expressed as RSD. Additionally, instrumental precision was determined through intra-day and inter-day repetitions of analytical standards.

To evaluate the linearity of the 18 target PAHs, both solvent-based and matrix-matched calibration curves were prepared. For the matrix-matched curves, sample extracts were spiked to yield concentrations identical to those of the solvent-based standards. As summarized in the validation tables (Tables S8–S12), the method demonstrated good linearity across the respective concentration ranges for all analytes, both in the solvent and within each matrix. For the solvent-based calibration curves, correlation coefficients (R) were higher than 0.99987, showing a highly robust instrumental response. Similarly, the matrix-matched calibration curves also showed good linearity, with R values above 0.99956 for ocean water, 0.99974 for river water, 0.99987 for WWTP effluent, and 0.99992 for WWTP influent. These results confirm that the presence of different water matrices does not compromise the linear performance of the method, ensuring reliable quantification across a wide range of environmental samples.

Method sensitivity was evaluated by analyzing the calibration curves for all target analytes. The LOD and LOQ, as well as the MDL and MQL, were calculated using the standard deviation of the response and the slope of the corresponding calibration curve, applying factors of 3.0 and 10, respectively. For the solvent-based calibration, Flu demonstrated the highest sensitivity, with the lowest LOD and LOQ at 0.147 µg/L and 0.489 µg/L, respectively. Conversely, Acen had the highest limits (LOD: 3.00 µg/L and LOQ: 9.99 µg/L). Acen was analyzed using a PAD detector, which offers lower sensitivity compared to fluorescence detection used for other target PAHs. For the matrix-matched calibration curves, as expected, the MDL and MQL values increased with the complexity of the matrix samples, from surface water to wastewater. In the sample extracts, the MDL and MQL ranges were 0.145–1.65 µg/L and 0.483–5.50 µg/L for ocean water; 0.0751–2.79 µg/L and 0.250–9.30 µg/L for river water; 0.192–6.89 µg/L and 0.641–23.0 µg/L for WWTP effluent; and 0.188–6.84 µg/L and 0.627–22.8 µg/L for WWTP influent (Tables S9–S12, Supplementary Materials). Considering the sample concentration factor (300x), the MDL and MQL values for the original water samples were determined by converting the extract concentrations from µg/L to ng/L and adjusting for the concentration factor, according to the following equations: (i) MDL_sample_ = MDL_extract_ (µg/L) × 1000 (ng/µg))/300 and (ii) MQL_sample_ (ng/L) = MQL_extract_ (µg/L) × 1000 (ng/µg))/300. The resulting MDL and MQL ranges were: 0.483–5.50 ng/L and 1.61–18.3 ng/L for ocean water; 0.251–9.30 ng/L and 0.835–31.0 ng/L for river water; 0.641–23.0 ng/L and 2.14–76.6 ng/L for WWTP effluents; and 0.627–22.8 ng/L and 2.09–76.0 ng/L for WWTP influents.

To evaluate the ME, the slope of each PAH in the matrix-matched calibration curve was compared with its corresponding slope in the solvent-based curve. A value of zero indicates no ME, whereas positive and negative values indicate signal enhancement and suppression, respectively. ME values between ±20% are considered indicative of minor ME [88]. As illustrated in Figure 10, the ME values for all 18 target PAHs in the four investigated water matrices (ocean, river, WWTP effluents, and WWTP influents)remained within the ±20% acceptance range, indicating minor matrix effects according to the criteria of Gros et al. [88]. Specifically, the highest observed signal variation remained below +18% for signal enhancement (for B[a]P in WWTP influents) and did not drop below −6% for signal suppression (for DB[ah]A in river water and Fln in WWTP influent). Ocean water was the only matrix in which all 18 PAHs exhibited signal enhancement. In contrast, river water showed predominantly signal suppression, with 13 of the 18 PAHs exhibiting negative ME values. The WWTP effluent and influent matrices generally showed signal enhancement for most of the investigated PAHs, although isolated cases of signal suppression were observed. These results indicate that the signal suppression or enhancement induced by the matrix was minimal among all environmental sample types, as all calculated ME values remained within the ±20% range. Thus, the developed analytical method demonstrates robust performance for complex environmental samples and is therefore suitable for the reliable quantification of the target PAHs in the investigated environmental water samples. These results also suggest that extensive sample cleanup may not be necessary under the conditions employed in the present study.

While previous validation parameters focused strictly on surface water and wastewater samples, extraction recovery experiments have been expanded to include drinking water samples as well. This addition allows the extraction method to be evaluated across a full spectrum of aquatic matrices, ranging from highly pure samples (e.g., drinking water) to complex samples (e.g., wastewater). Recovery was evaluated by comparing the peak areas of pre- and post-spiked samples after SPE. As shown in Figure 11, the obtained recoveries ranged from 84.6% to 104% in drinking water (*n* = 2), 83.3% to 97.7% in ocean water (*n* = 2), 71.8% to 100.2% in river water (*n* = 3), 89.0% to 106% in WWTP effluent (*n* = 3), and 87.5% to 110% in WWTP influent. Overall, recoveries fell between 71.8% and 110% across all matrices, with RSD below 10%. These results demonstrate the high efficiency, robustness, and overall success of the SPE method for the target compounds, regardless of matrix complexity.

Finally, instrumental precision was confirmed through intra-day and inter-day repeatability studies using an analytical standard (Naph (99.9 µg/L), Acen (210 µg/L), Ace (99.9 µg/L), Flu (19.7 µg/L), Phe (10.0 µg/L), Ant (10.0 µg/L), Fln (19.7 µg/L), Pyr (9.70 µg/L), B[a]A (9.8 µg/L), Chry (9.8 µg/L), B[b]Ft+ B[j]Ft (40 µg/L), B[k]Ft (9.90 µg/L), B[a]P (10.0 µg/L), DB[a,l]P (20.0 µg/L), DB[ah]A (19.9 µg/L), B[ghi]P (19.8 µg/L), and InP (10.0 µg/L)). Intra-day precision was evaluated through consecutive injections within the same day (*n* = 12), which yielded RSD values ranging from 0.293% (B[b]Ft + B[j]Ft) to 1.96% (InP). Inter-day precision, assessed by analyzing the same standard across six different days over an 18-day period, resulted in RSD values between 1.20% (B[k]Ft) and 8.81% (B[a]P). These results demonstrate excellent instrumental stability and reproducibility over time.

### 4.9. Samples

To evaluate the applicability of the developed extraction method, 25 environmental water samples were analyzed. For each sample, three independent extractions were performed.

Across the entire dataset (18 PAHs × 25 samples = 450 total data points; Table 1), 60.4% of results were not detected, while 39.6% were detected. Within the detected fraction, the highest proportion corresponded to values below the MQL (18.4%), followed by values above MQL (10.9%), and the remaining values were detected below the MDL (10.2%). Six target compounds (Ant, Fln, B(a)A, B(b)Ft + B(j)Ft, B(k)Ft, and B[a]P) remained consistently below the limits (MDL/MQL) across all matrices. Among the quantified compounds, Naph, Ace, and Phe were the most frequently measured.

To further explore the distribution of individual PAHs, the detection frequencies were evaluated. Flu and Phe were the most frequently detected PAHs, with a detection frequency of 100%. Naph and Ace were also highly prevalent (80.0%), while Pyr, Ant, and Fln followed a descending trend (76.0%, 68.0%, and 56.0%), respectively. In contrast, lower detection frequencies were observed for B[k]Ft (40%), B[b]F + B[j]F (36.0%), and B[a]P (28.0%), while Acen (8.00%) and B[a]A (4.00%) were rarely detected. The remaining PAHs (Chry, DB[a,l]P, DB[a,h]A, B[ghi]P, and InP) were never detected. These results showed that low molecular weight PAHs are more frequently detected in water samples, whereas high molecular weight PAHs are less frequently detected and, when present, generally remain below the MDL and MQL. The higher water solubility of these lighter compounds enhances their persistence in aqueous samples, whereas heavier, more hydrophobic PAHs tend to partition out of the water phase (Table S2, Supplementary Materials).

The levels and number of PAHs detected varied from sample to sample, reflecting distinct diversity profiles. Focusing on the number of detected analytes, the highest values for PAHs (11–12 compounds) were observed in ocean samples (OW1, OW2, and OW3), followed closely by river samples RW9 and RW10 (10–11 compounds), whereas RW4, RW5, and RW6 samples showed the lowest values, with only two to three detected compounds. This spatial variability indicates that marine systems may act as large-scale environmental sinks, accumulating trace inputs from diverse sources, such as atmospheric deposition and maritime activities, including commercial and tourism shipping [89,90]. It should be highlighted that the large volume of water in the oceans may also contribute to the dilution of contaminant concentrations. Regardless of the detected concentrations, wastewater samples had the highest PAH levels, reaching maximum concentrations of 40.4 ng/L for Naph, 2009 ng/L for Ace, and 45.2 ng/L for Phe (WWTP I1). Moreover, Acen was detected in two samples, with concentrations below the MDL in WWTP I1 and 50.4 ng/L in R2. Flu showed a 100% detection frequency, although it was quantified only in one sample (WWTP I1, 10.0 ng/L). Additionally, six compounds were only detected below the MDL or MQL thresholds: Ant (detected in 17 samples), Fln (detected in 14 samples), B(a)A (detected in 1 sample), B(b)Ft + B(j)Ft (detected in 9 samples), B(k)Ft (detected in 10 samples), and B(a)P (detected in 7 samples).

Among the quantified PAHs, RSD values ranged from 0.712 to 6.2% in ocean water (OW), from 0.554 to 13% in river water (RW), from 0.0304 to 13% in WWTP effluent (WWTP-E), and from 0.120 to 10% in WWTP influent (WWTP-I). Overall, the relatively low RSD values observed for most PAHs indicate good precision and reproducibility of the analytical method across the different water matrices. Nevertheless, the results demonstrate that the method provides satisfactory precision for the quantification of PAHs in the analyzed water samples.

EU Directive 105/2008 [91] sets environmental quality standards for surface water, expressed as µg/L as an annual average (AA) and a maximum admissible concentration (MAC). These standards include limits for Flu (AA = 0.1 µg/L, MAC = 1 µg/L), Naph (AA = 2.4 µg/L), Ant (AA = 0.1 µg/L, MAC = 0.4 µg/L), B[a]P (AA = 0.05 µg/L, MAC = 0.1 µg/L), and for the sums of B[b]Ft and B[k]Ft (AA = 0.03 µg/L) and B[ghi]P and InP (AA = 0.002 µg/L). The present data were compared with the results of the environmental quality standards, and the obtained concentrations in the present study (Table 1) for the 25 analyzed samples were all below the regulatory limits.

Mezzanotte et al. [92] observed that low molecular weight PAHs were the predominant compounds found in wastewater, with Ace and Naph representing the major analytes in the WWTP influent samples. In contrast, high molecular weight PAHs occurred at lower concentrations, and compounds such as B[ghi]P and DB[ah]A were never detected. Similarly, Semenov et al. [93] reported that 2–3-ring PAHs predominated in water samples, whereas 4–6-ring PAHs were mainly associated with sediments, reflecting the fractionation behavior of PAHs within the soil–water system. Comparable findings were observed in the present study (Table 1), where low molecular weight PAHs were more abundant than high molecular weight PAHs. Therefore, the observations reported by Mezzanotte et al. [92], Semenov et al. [93], and the present study suggest that the physicochemical properties of the compounds, including vapor pressure, hydrophobicity, and water solubility, may play an important role in the occurrence and distribution of lowand high molecular weight PAHs (Table S2, Supplementary Materials). In seawater samples, Shi et al. [94] reported that the quantified concentrations of PAHs ranged from 0.2 ng/L (B[a]P) to 25.8 ng/L (Naph). The detected PAHs included Naph, Acen, Ace, Flu, Phe, Ant, Fln, Pyr, B[a]P, and B[ghi]P, while B[a]A, Chry, B[b]Ft, B[k]Ft, and DB[ah]A were not detected. Furthermore, Montuori et al. [30] reported that low molecular weight PAH levels were higher in water samples, while high molecular weight PAHs predominated in sediment samples. The authors also noted that PAH concentrations in the water and sediment near the Sele River were heavily influenced by industrial wastewater and vehicle emissions.

## 5. Conclusions

Environmental compartments receive continuous inputs from atmospheric deposition, industrial discharges, urban runoff, and petroleum-related activities. Monitoring contaminant levels in these compartments is crucial for assessing environmental contamination and ecological risks. It is also important for evaluating potential impacts on human health.

The optimization of SPE for the analysis of PAHs in aqueous samples proved challenging, primarily because of the high volatility of low molecular weight compounds. While testing different sorbents and elution solvents yielded no initial improvements, the addition of an organic solvent to the water sample prior to extraction substantially enhanced the recovery of heavier PAHs. Although using a “keeper” solvent post-elution provided moderate recovery gains, optimal performance was not achieved. The evaporation step was ultimately identified as the most critical stage of the workflow. By implementing a two-stage evaporation process, avoiding evaporation to complete dryness, analyte recovery and overall method performance were substantially improved. Furthermore, the investigation into vacuum pressure and washing and drying steps after loading the water sample through the cartridge revealed that a controlled pressure of 5 mmHg, combined with the omission of washing and drying steps, was the best procedure to minimize analyte loss.

The analytical method was successfully validated, demonstrating excellent linearity (R > 0.99952), high sensitivity, and robust SPE recoveries (72–110%, RSD < 10%) across a wide range of aquatic matrices, from DW to complex wastewaters samples. Low intra- and inter-day RSDs (≤8.81%) also confirmed the high instrumental stability of the method over time.

Quantified concentrations ranged from 5.39 to 30.8 ng/L in OW samples, 2.01 to 50.4 ng/L in RW waters, 6.00 to 716 ng/L in WWTP effluents, and 3.27 to 2009 ng/L in WWTP influents. The successful application of this method to this variety of water matrices demonstrates the robustness and real-world applicability of the optimized procedure. This approach enhances the trace-level detection of PAHs in aquatic ecosystems, providing critical data to support environmental risk assessments.

## Figures and Tables

**Figure 1:**
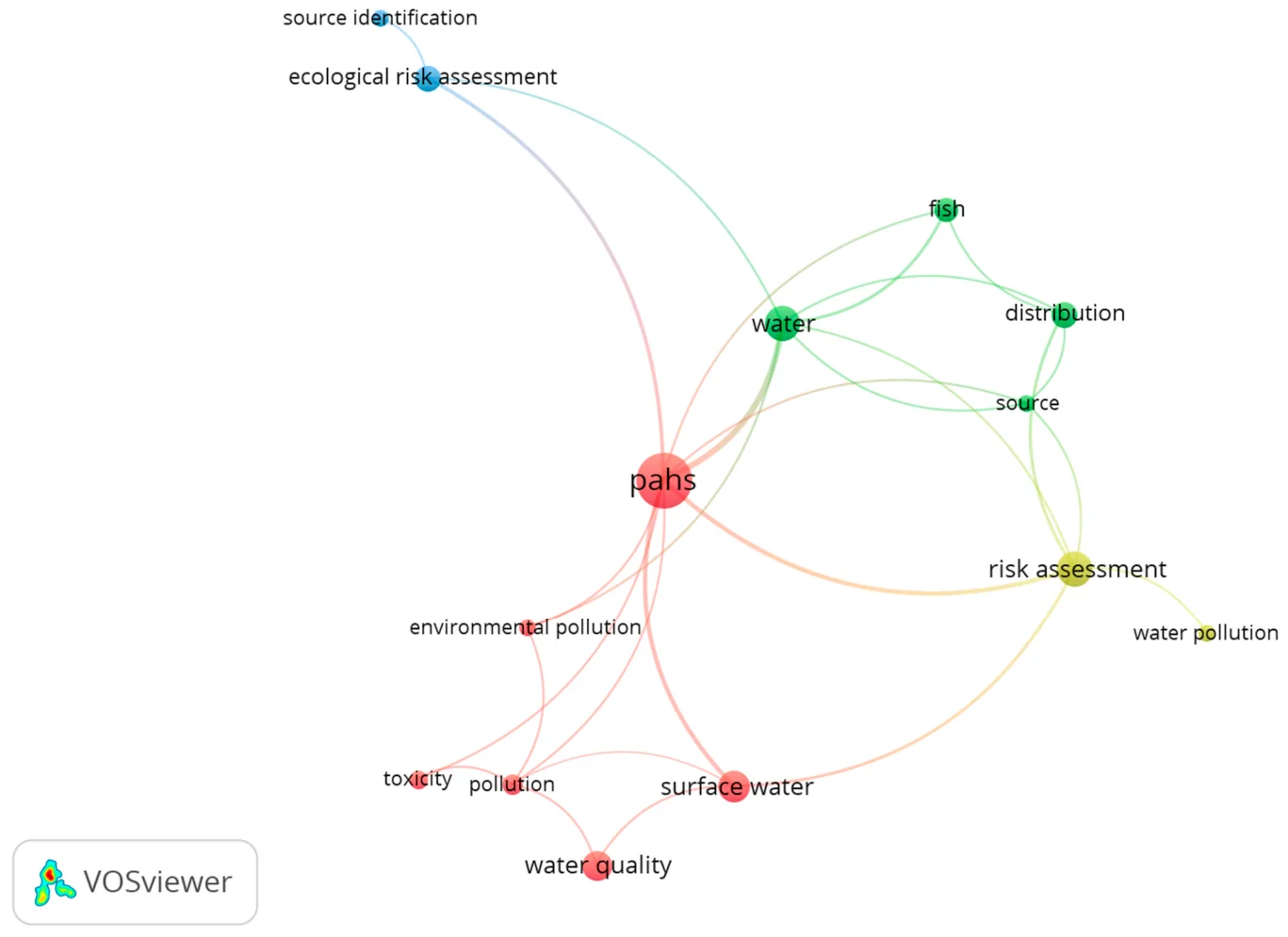
Keyword co-occurrence network obtained using the VOSviewer programme based on the 14 most frequently used terms related to PAHs in environmental research. (Nodes represent keywords, node size reflects occurrence frequency, colors denote distinct research clusters, and connecting lines indicate co-occurrence links between terms.)

**Figure 2:**
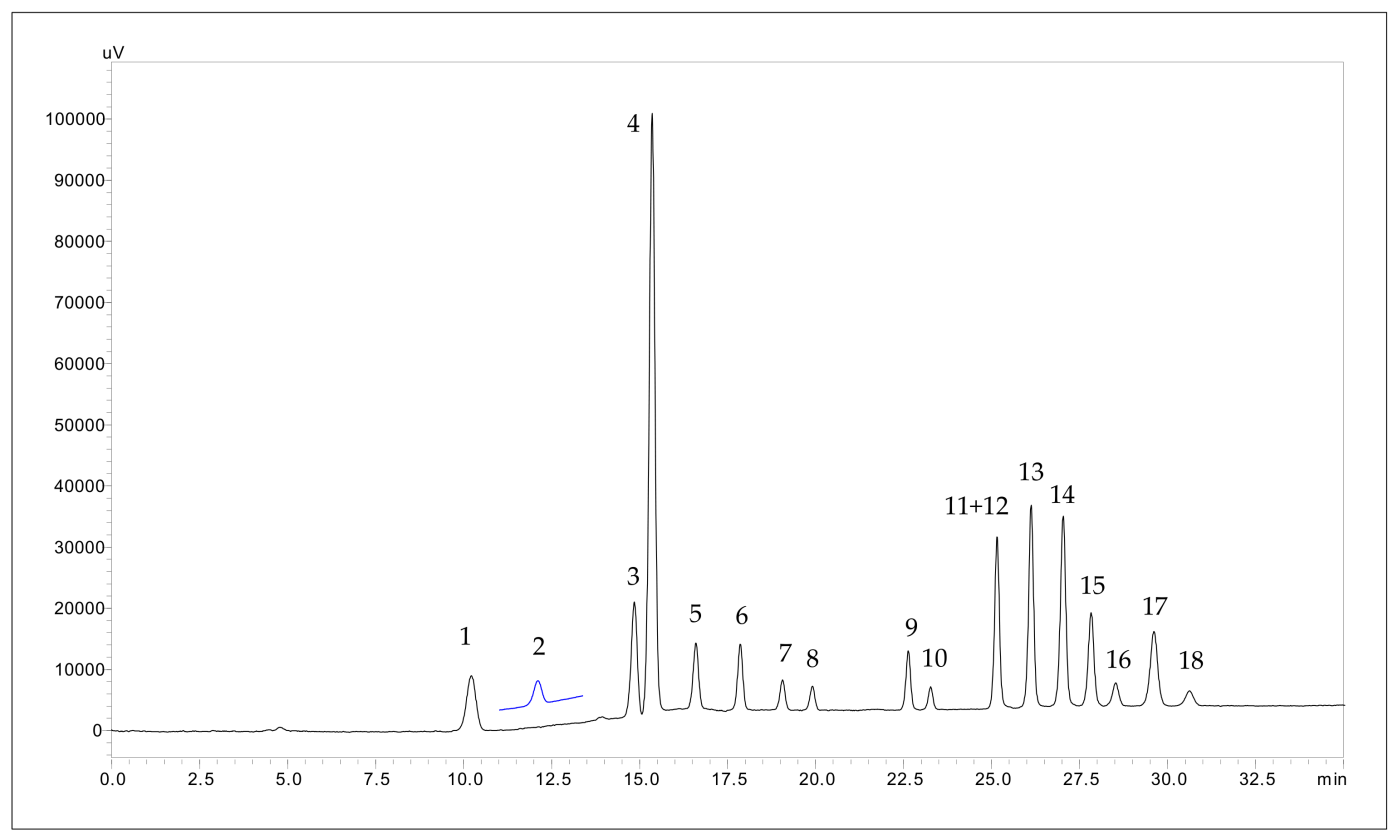
Chromatographic separation of the 18 target PAHs. The black trace represents compounds detected by the FLD detector, and the blue line corresponds to the PAH detected by the PAD detector (peak identification: (1) Naph, (2) Acen, (3) Ace, (4) Flu, (5) Phe, (6) Ant, (7) Fln, (8) Pyr, (9) B[*a*]A, (10) Chry, (11 + 12) B[*b*]Ft + B[*j*]Ft, (13) B[*k*]Ft, (14) B[*a*]P, (15) DB[*ah*]A, (16) B[*ghi*]P, (17) DB[*a,l*]P, and (18) InP).

**Figure 3:**
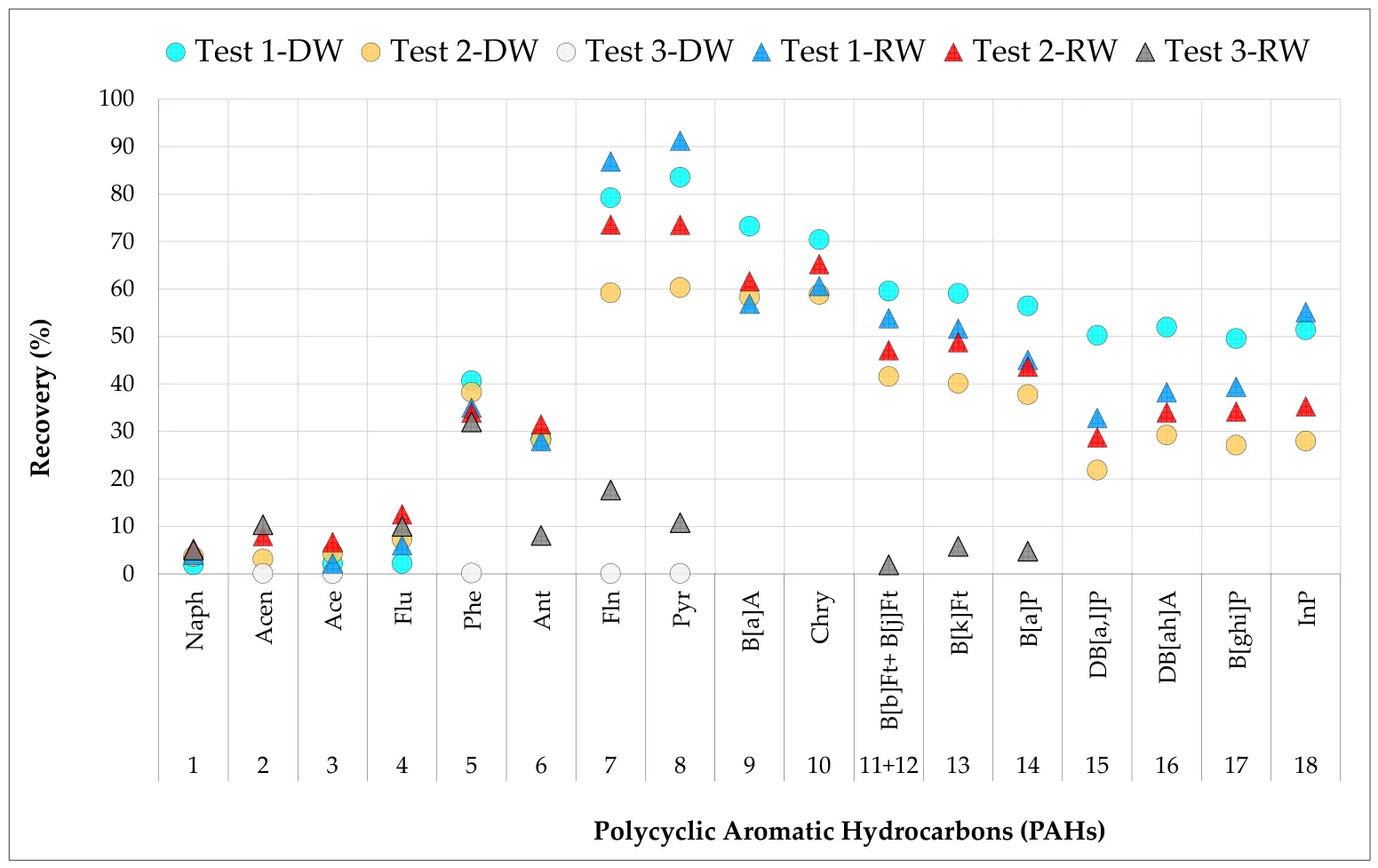
Recoveries (%) of 18 polycyclic aromatic hydrocarbons from drinking water (DW) and river water (RW) samples using two sorbents (Strata-PAH and Strata-X) under three different extraction procedures (Tests 1, 2, and 3).

**Figure 4:**
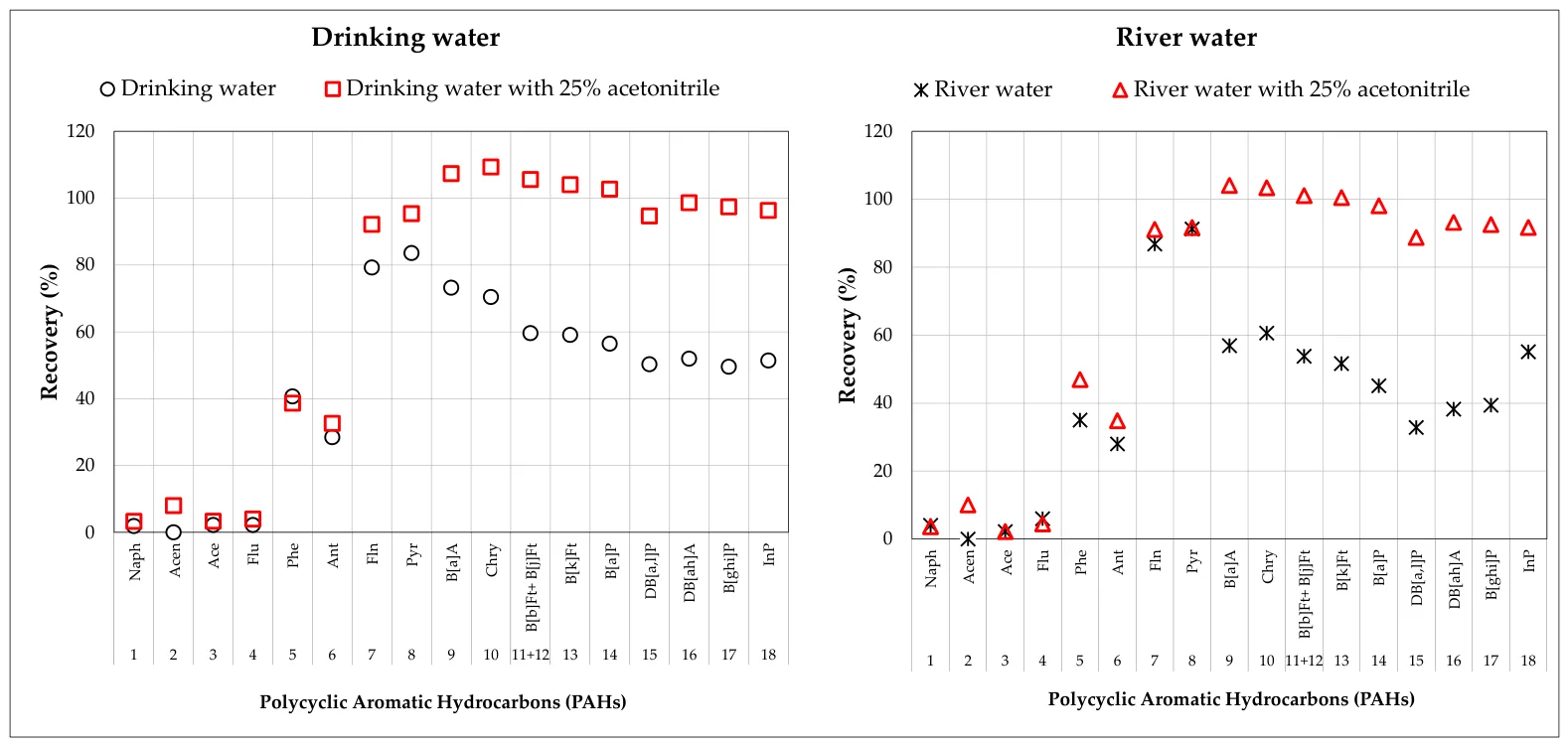
Recoveries of the target compounds with and without the addition of an organic solvent to drinking water (DW) and river water (RW) samples prior to SPE.

**Figure 5:**
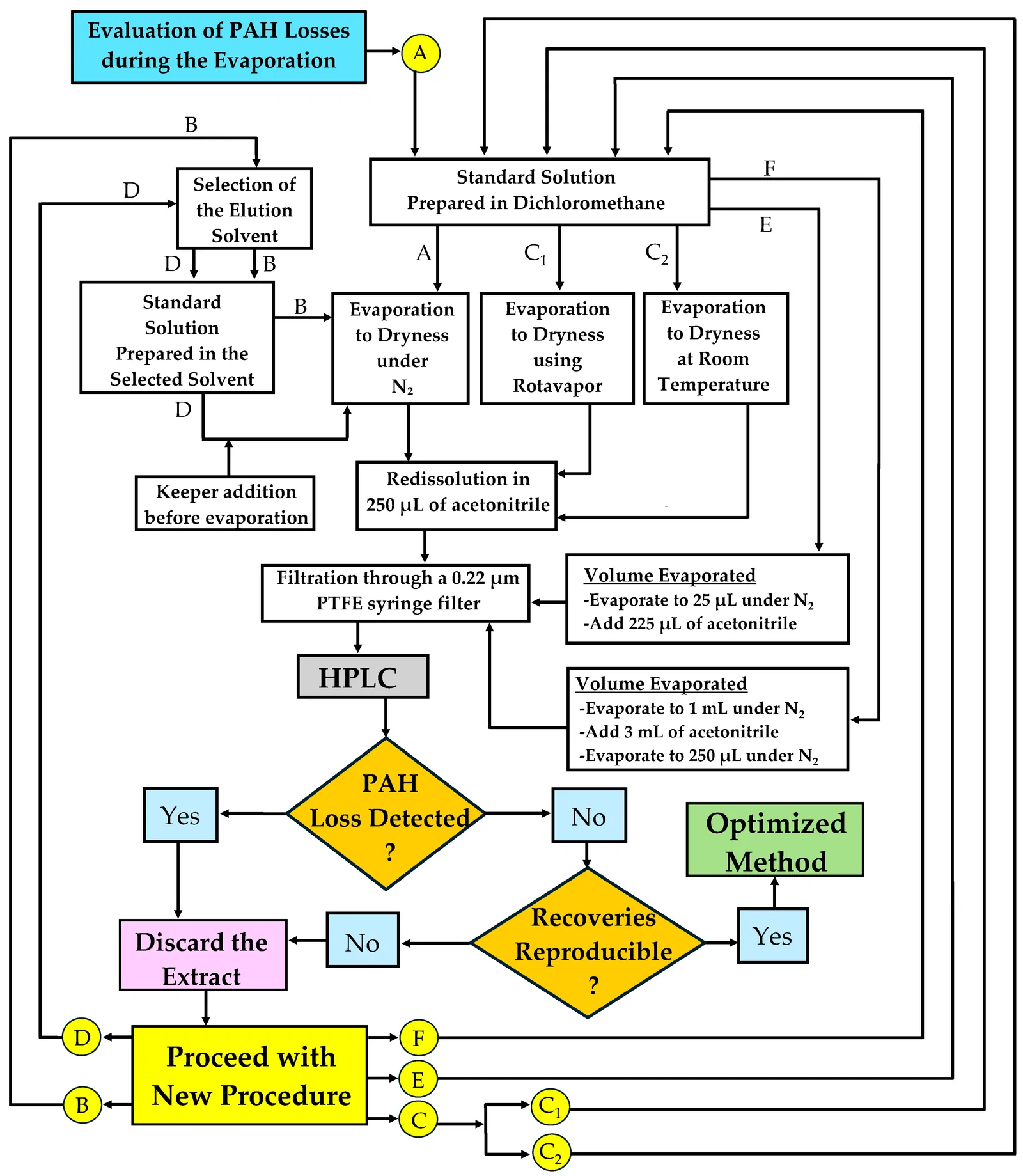
Workflow of the experiments conducted to identify the potential losses of lighter polycyclic aromatic hydrocarbons (PAHs) during the evaporation step.

**Figure 6:**
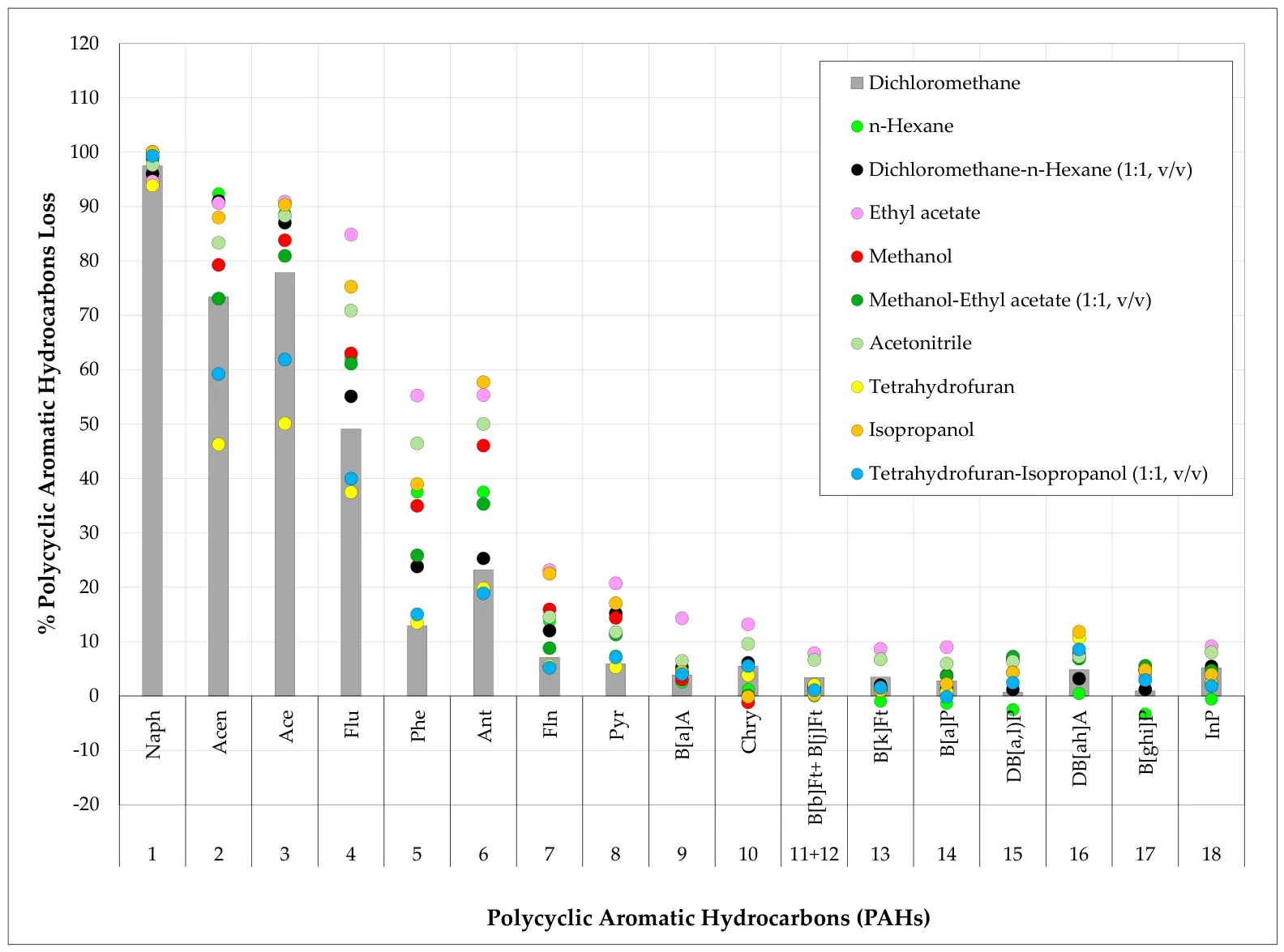
Percentage of polycyclic aromatic hydrocarbon (PAH) compounds lost during evaporation for each tested solvent and solvent mixture.

**Figure 7:**
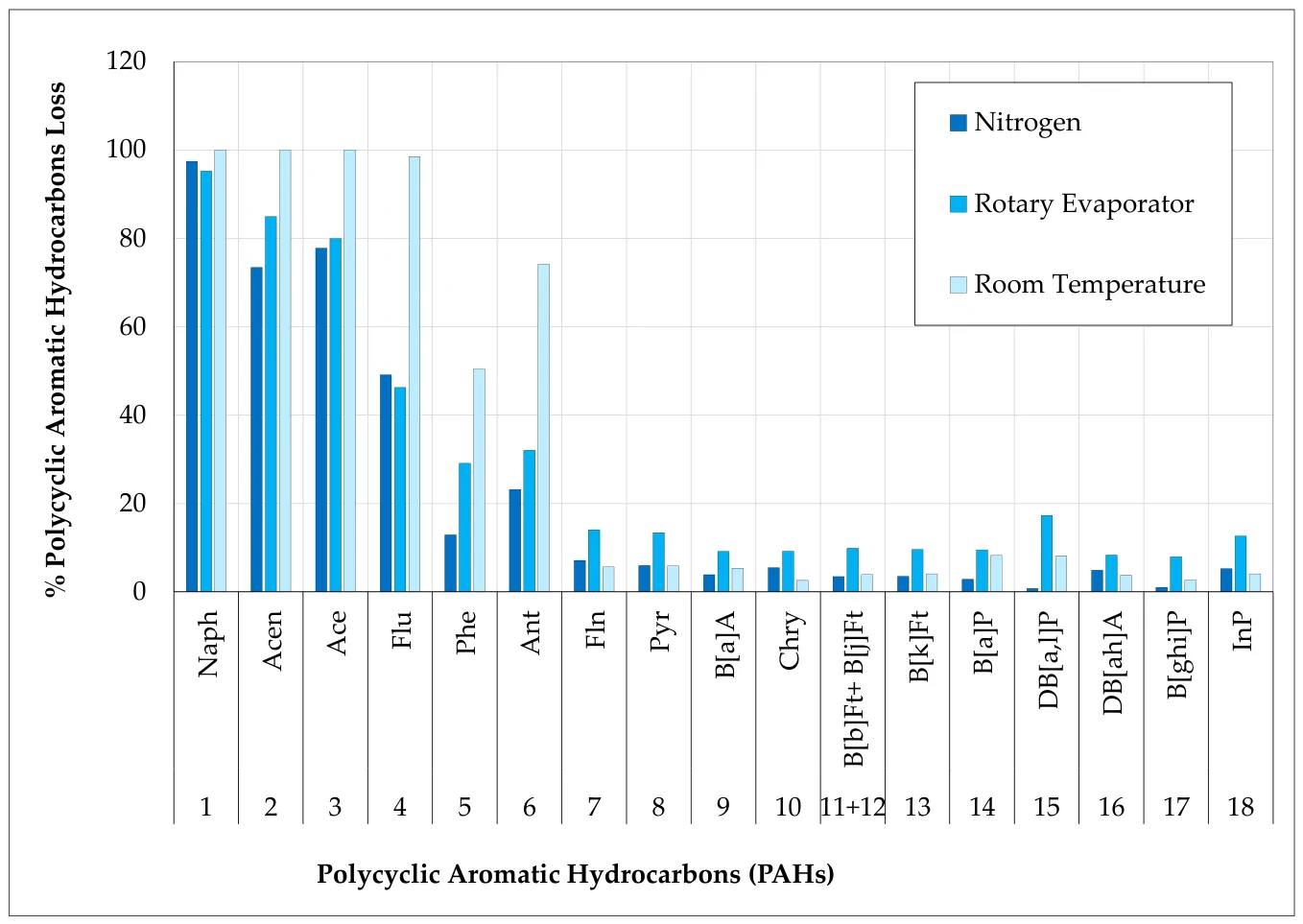
Percentage losses of the studied polycyclic aromatic hydrocarbon (PAH) across three evaporation techniques: nitrogen stream, rotary evaporation, and room temperature.

**Figure 8:**
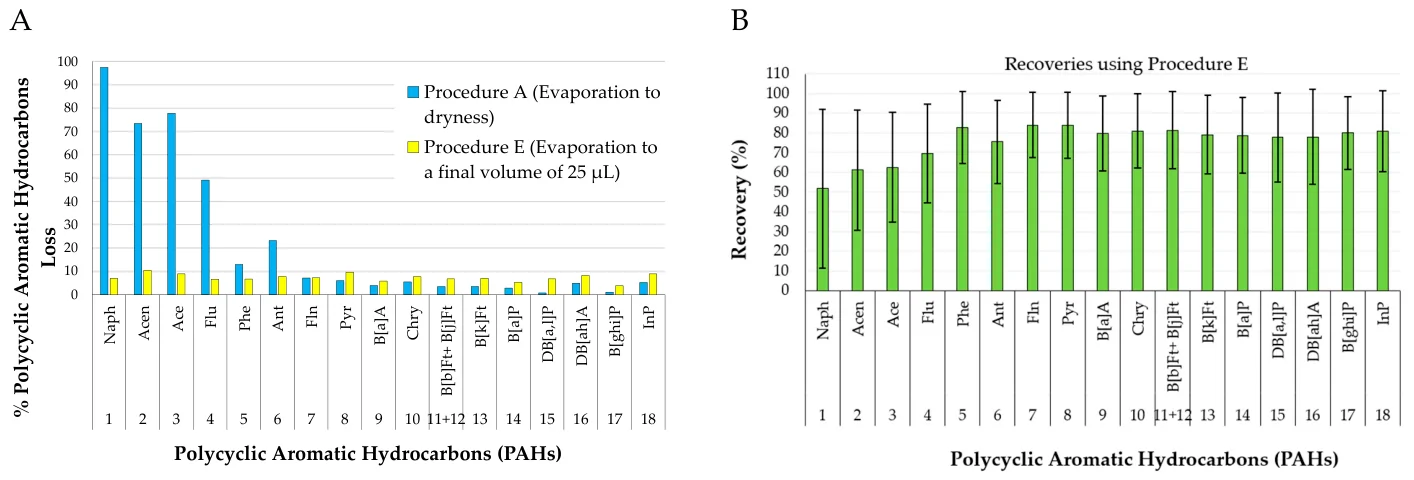
Evaluation of solvent evaporation efficiency: (**A**) comparison of analyte loss (%) following evaporation of the standard solution under a nitrogen stream to dryness (Procedure A) versus partial evaporation to a final volume of 25 µL (Procedure E), without performing the complete SPE analytical workflow (i.e., evaluating the evaporation step alone); (**B**) mean recovery (%) (*n* = 15) obtained using the complete SPE analytical workflow for drinking water, incorporating Procedure E (partial evaporation to a final volume of 25 µL) in the evaporation step.

**Figure 9:**
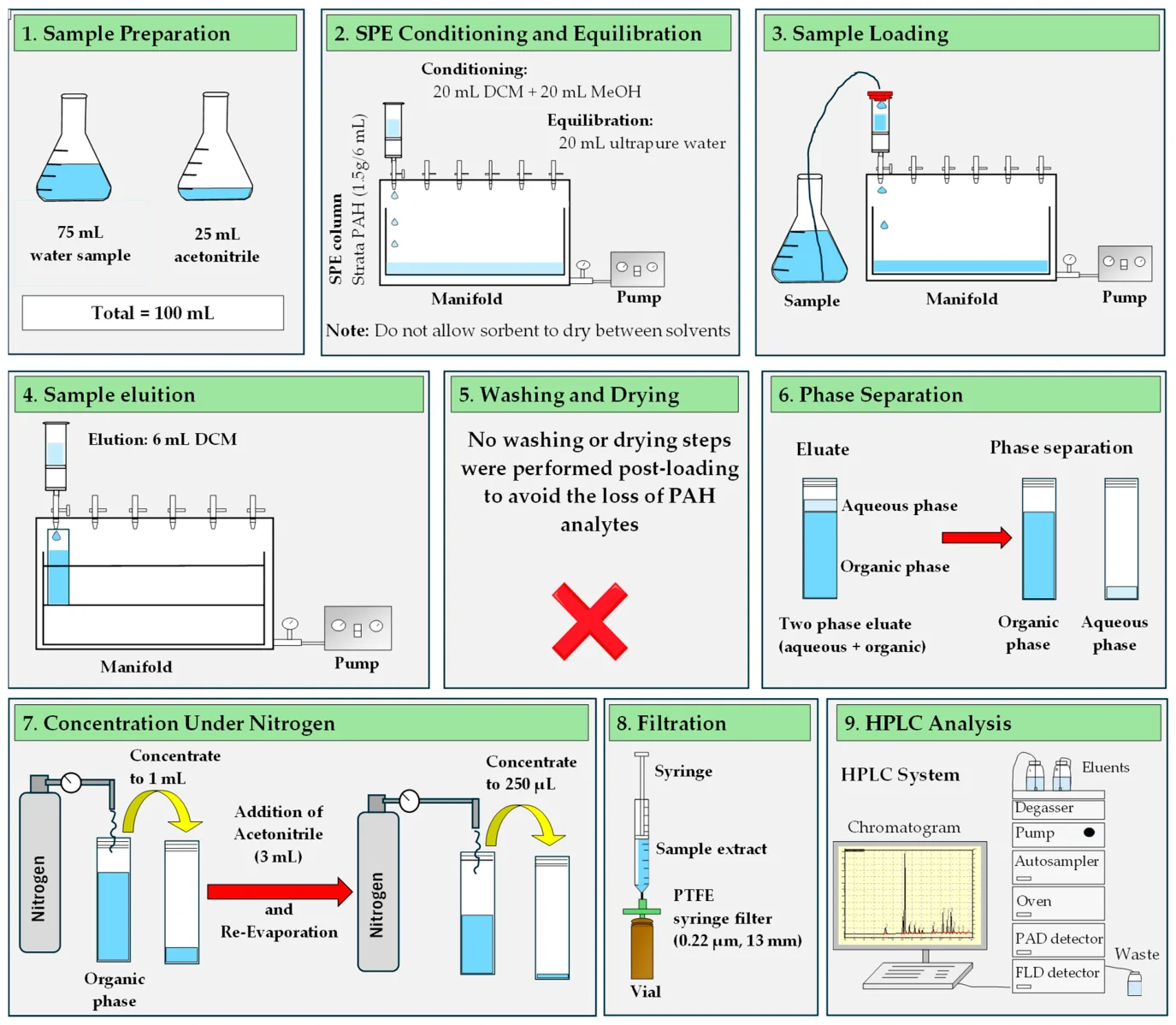
Schematic workflow of the solid-phase extraction procedure for the extraction and concentration of polycyclic aromatic hydrocarbons (PAHs) from aqueous matrices.

**Figure 10:**
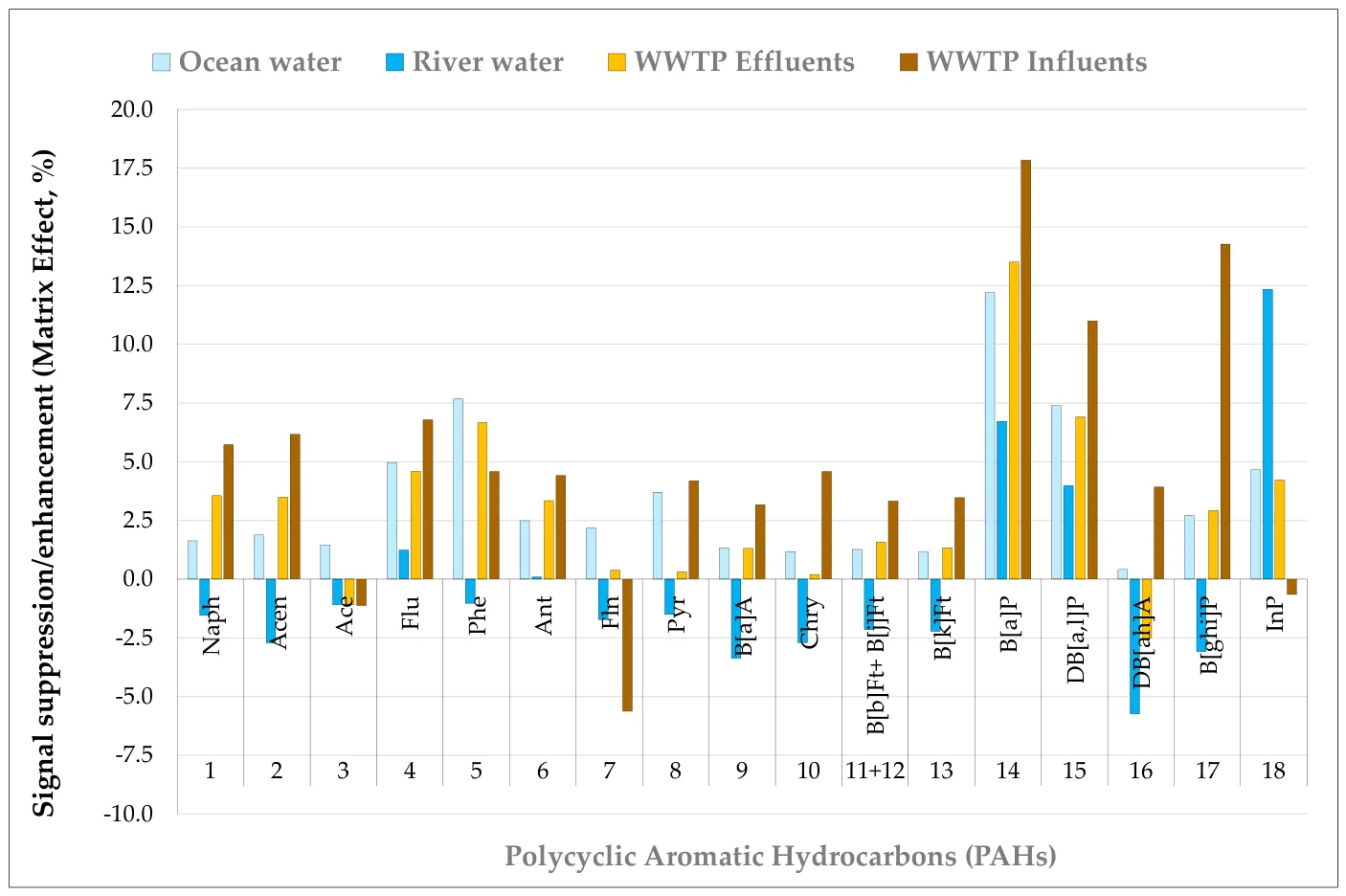
Matrix effects (ME, %) determined for 18 polycyclic aromatic hydrocarbons (PAHs) across different environmental water matrices: ocean water, river water, wastewater treatment plant (WWTP) effluents, and WWTP influents.

**Figure 11:**
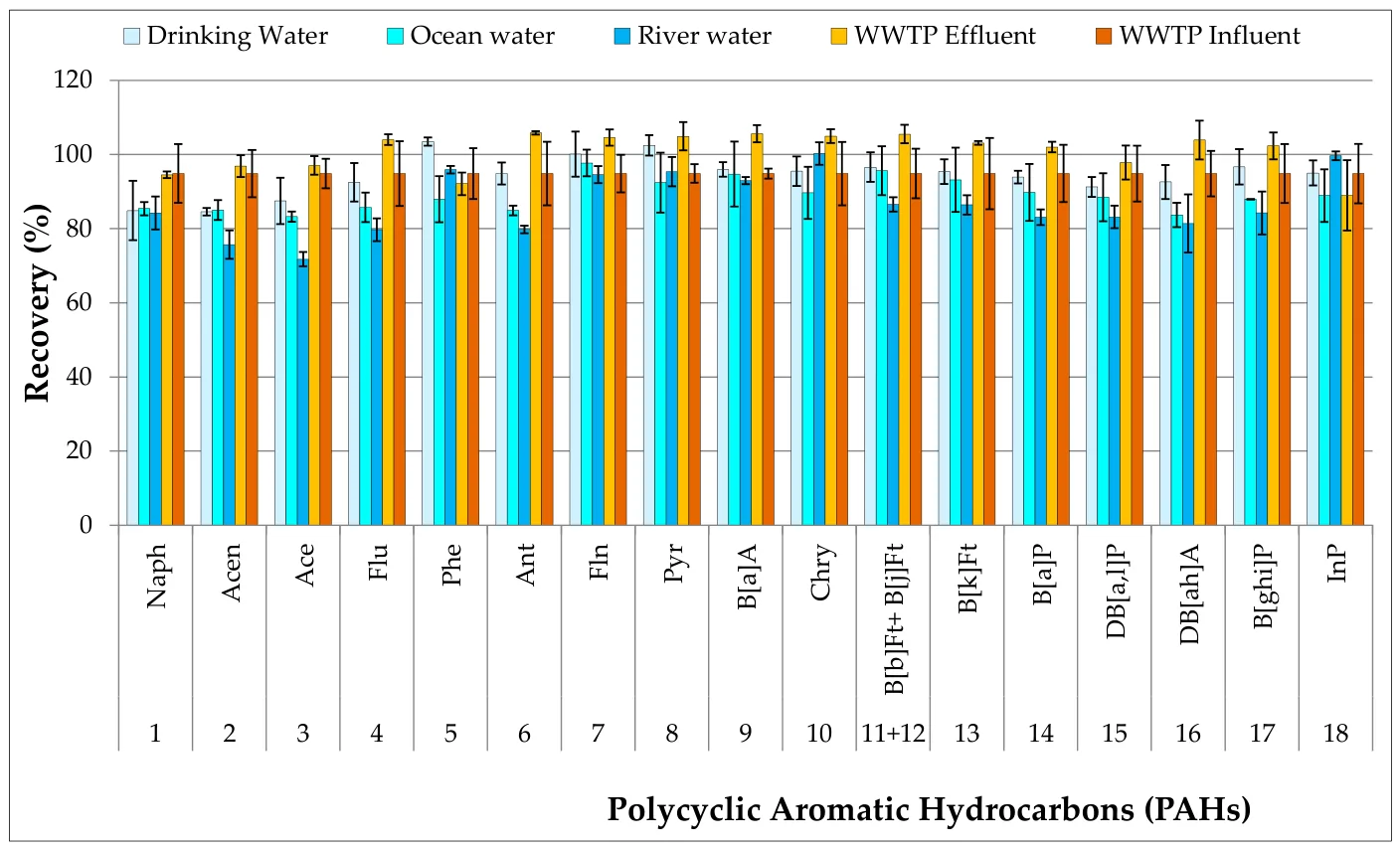
Recoveries of the 18 polycyclic aromatic hydrocarbons (PAHs) obtained for drinking water, ocean water, river water, WWTP effluent, and WWTP influent samples.

**Table 1:** Concentration (ng/L) of the studied polycyclic aromatic hydrocarbons (PAHs) in ocean (OW) and river (RW) waters and effluent (E) and influent (I) wastewater samples (legend: n.d.-Not detected, <MDL-below method detection limit, and <MQL-below method quantification limit). Note: Green indicates concentrations ≥ MQL, yellow indicates concentrations < MQL, and light blue indicates concentrations < MDL.

	Polycyclic Aromatic Hydrocarbons Concentration (ng/L)																
Sample	1	2	3	4	5	6	7	8	9	10	11 + 12	13	14	15	16	17	18
	Naph	Acen	Ace	Flu	Phe	Ant	Fln	Pyr	B[a]A	Chry	B[b]Ft + B[j]Ft	B[k]Ft	B[a]P	DB[a,l]P	DB[ah]A	B[ghi]P	InP
OW1	28.3	n.d.	<MQL	<MQL	5.51	<MDL	<MQL	<MQL	<MDL	n.d.	<MDL	<MDL	<MDL	n.d.	n.d.	n.d.	n.d.
OW2	28.6	n.d.	<MQL	<MDL	5.39	<MDL	<MDL	<MQL	n.d.	n.d.	<MDL	<MDL	<MDL	n.d.	n.d.	n.d.	n.d.
OW3	30.8	n.d.	<MQL	<MQL	5.73	<MDL	<MDL	<MQL	n.d.	n.d.	<MDL	<MDL	<MDL	n.d.	n.d.	n.d.	n.d.
RW1	37.2	n.d.	10.1	<MQL	<MDL	n.d.	n.d.	<MDL	n.d.	n.d.	<MDL	<MDL	n.d.	n.d.	n.d.	n.d.	n.d.
RW2	37.0	50.4	39.8	<MDL	<MDL	n.d.	n.d.	n.d.	n.d.	n.d.	<MDL	n.d.	n.d.	n.d.	n.d.	n.d.	n.d.
RW3	n.d.	n.d.	n.d.	<MDL	<MDL	n.d.	n.d.	n.d.	n.d.	n.d.	n.d.	n.d.	<MDL	n.d.	n.d.	n.d.	n.d.
RW4	n.d.	n.d.	n.d.	<MDL	<MDL	n.d.	n.d.	n.d.	n.d.	n.d.	n.d.	n.d.	n.d.	n.d.	n.d.	n.d.	n.d.
RW5	n.d.	n.d.	n.d.	<MDL	<MDL	n.d.	n.d.	n.d.	n.d.	n.d.	n.d.	n.d.	n.d.	n.d.	n.d.	n.d.	n.d.
RW6	n.d.	n.d.	n.d.	<MDL	<MDL	n.d.	n.d.	n.d.	n.d.	n.d.	<MDL	<MDL	n.d.	n.d.	n.d.	n.d.	n.d.
RW7	14.3	n.d.	n.d.	<MQL	<MQL	n.d.	n.d.	n.d.	n.d.	n.d.	n.d.	n.d.	n.d.	n.d.	n.d.	n.d.	n.d.
RW8	14.3	n.d.	7.69	<MDL	<MQL	n.d.	n.d.	<MDL	n.d.	n.d.	<MDL	<MDL	<MDL	n.d.	n.d.	n.d.	n.d.
RW9	25.2	n.d.	<MQL	<MQL	2.16	<MDL	<MDL	<MQL	n.d.	n.d.	<MDL	<MDL	<MDL	n.d.	n.d.	n.d.	n.d.
RW10	19.4	n.d.	<MQL	<MQL	<MQL	<MDL	n.d.	<MQL	n.d.	n.d.	<MDL	<MDL	<MDL	n.d.	n.d.	n.d.	n.d.
RW11	<MQL	n.d.	5.93	<MDL	2.01	<MDL	n.d.	<MDL	n.d.	n.d.	n.d.	<MDL	n.d.	n.d.	n.d.	n.d.	n.d.
E1	36.0	n.d.	608	<MQL	6.74	<MQL	<MDL	<MQL	n.d.	n.d.	n.d.	n.d.	n.d.	n.d.	n.d.	n.d.	n.d.
E2	38.1	n.d.	716	<MQL	9.23	<MQL	<MDL	<MQL	n.d.	n.d.	n.d.	n.d.	n.d.	n.d.	n.d.	n.d.	n.d.
E3	<MQL	n.d.	338	<MDL	6.22	<MDL	<MDL	<MQL	n.d.	n.d.	n.d.	n.d.	n.d.	n.d.	n.d.	n.d.	n.d.
E4	32.8	n.d.	577	<MQL	6.04	<MQL	<MDL	<MQL	n.d.	n.d.	n.d.	n.d.	n.d.	n.d.	n.d.	n.d.	n.d.
E5	<MQL	n.d.	615	<MQL	7.99	<MQL	<MDL	<MQL	n.d.	n.d.	n.d.	n.d.	n.d.	n.d.	n.d.	n.d.	n.d.
E6	37.5	n.d.	323	<MQL	6.00	<MQL	<MDL	<MQL	n.d.	n.d.	n.d.	n.d.	n.d.	n.d.	n.d.	n.d.	n.d.
I1	40.4	<MDL	2009	10.0	45.2	<MQL	<MDL	4.14	n.d.	n.d.	n.d.	n.d.	n.d.	n.d.	n.d.	n.d.	n.d.
I2	<MQL	n.d.	619	<MDL	7.15	<MDL	<MDL	<MQL	n.d.	n.d.	n.d.	n.d.	n.d.	n.d.	n.d.	n.d.	n.d.
I3	n.d.	n.d.	252	<MDL	7.97	<MDL	<MDL	3.27	n.d.	n.d.	n.d.	<MDL	n.d.	n.d.	n.d.	n.d.	n.d.
I4	<MQL	n.d.	575	<MDL	7.95	<MDL	<MDL	<MQL	n.d.	n.d.	n.d.	n.d.	n.d.	n.d.	n.d.	n.d.	n.d.
I5	<MQL	n.d.	485	<MDL	5.85	<MDL	n.d.	<MQL	n.d.	n.d.	n.d.	n.d.	n.d.	n.d.	n.d.	n.d.	n.d.

## Data Availability

The original contributions presented in this study are included in the article/Supplementary Material. Further inquiries can be directed to the corresponding authors.

## Supplementary Materials

The following supporting information can be downloaded at: https://www.mdpi.com/article/10.3390/jox16060186/s1. Table S1. Experimental conditions for Tests 1, 2, and 3 using Strata PAH and Strata X sorbents in preliminary evaluations for the extraction of polycyclic aromatic hydrocarbons (PAHs) in water samples; Table S2. Physicochemical properties of the study polycyclic aromatic hydrocarbons (PAHs), target solvents, and keepers; Table S3. The literature research for the extraction and evaporation methodologies for polycyclic aromatic hydrocarbons (PAHs) analysis; Table S4. List of the tested primary solvents, keepers and their percentage or volume added; Table S5A. Percentage of loss for the 18 polycyclic aromatic hydrocarbons (PAHs) obtained when different keepers and solvents were used; Table S5B. Summary of the best results obtained for the most volatile PAHs among the 143 experimental tests; Table S6. Spiking concentrations of polycyclic aromatic hydrocarbons (PAHs) for drinking water analysis using Procedure F (Figure 5); Table S7. Recoveries obtained in ten fortification levels and the average recovery and relative standard deviation (RSD, %) for each polycyclic aromatic hydrocarbon (PAHs); Table S8. Concentration ranges and number of calibration points used, and correlation coefficients (R), slope, Y-intercept, limits of detection (LOD), and limits of quantification (LOQ) obtained for the polycyclic aromatic hydrocarbons (PAHs) using calibration curves prepared in solvent; Table S9. Concentration ranges (CR) and number of calibration points (NCP) used, correlation coefficients (R), slope, y-intercept, and method detection (MDL) and quantification (MQL) limits determined in the ocean sample extract for the polycyclic aromatic hydrocarbons (PAHs). The corresponding calibration parameters for the ocean water sample were calculated by applying the concentration factor and the sample volume; Table S10. Concentration ranges (CR) and number of calibration points (NCP) used, correlation coefficients (R), slope, y-intercept, and method detection (MDL) and quantification (MQL) limits determined in the river sample extract for the polycyclic aromatic hydrocarbons (PAHs). The corresponding calibration parameters for the river water sample were calculated by applying the concentration factor and the sample volume; Table S11. Concentration ranges (CR) and number of calibration points (NCP) used, correlation coefficients (R), slope, y-intercept, and method detection (MDL) and quantification (MQL) limits determined in the WWTP effluent sample extract for the polycyclic aromatic hydrocarbons (PAHs). The corresponding calibration parameters for the WWTP effluent water sample were calculated by applying the concentration factor and the sample volume; Table S12. Concentration ranges (CR) and number of calibration points (NCP) used, correlation coefficients (R), slope, y-intercept, and method detection (MDL) and quantification (MQL) limits determined in the WWTP influent sample extract for the polycyclic aromatic hydrocarbons (PAHs). The corresponding calibration parameters for the WWTP influent water sample were calculated by applying the concentration factor and the sample volume; Figure S1. Recoveries of 18 polycyclic aromatic hydrocarbons (PAHs) from river water using Strata-X cartridges across Tests 2, 3, and 4 with different elution solvents: dichloromethane, methanol, and a dichloromethane:methanol (1:1, *v*/*v*) mixture; Figure S2. Recovery percentages of the studied polycyclic aromatic hydrocarbons (PAHs) from river water samples (100 mL and 250 mL) using Tests 1, 2, and 3; Figure S3. General procedure used for the extraction of polycyclic aromatic hydrocarbons (PAHs) in water samples; Figure S4. Overlay chromatogram of a standard mixture of polycyclic aromatic hydrocarbons (PAHs) without PAHs standard filtration (black) and with PAHs standard filtration using a PTFE syringe filter (pink); Figure S5. Physicochemical properties of the studied polycyclic aromatic hydrocarbon (PAH) compounds (A), primary solvents (B), and keepers (C) (Molecular Weight, log Kow, boiling point, and vapor pressure were obtained from https://pubchem.ncbi.nlm.nih.gov/); Figure S6. Percentage loss for each polycyclic aromatic hydrocarbon (PAH) in the different proportions of dichloromethane-*n*-hexane-acetone; Figure S7. Evaluation of the impact of SPE operational steps (vacuum pressure, cartridge washing, and drying duration) on polycyclic aromatic hydrocarbons (PAHs) recoveries during method optimization; Figure S8. Percentage of polycyclic aromatic hydrocarbons (PAHs) loss during various evaporation protocols: evaporation to dryness (with and without a lipid ‘keeper’), evaporation to a final volume of 25 µL, and a two-stage evaporation process to a final volume of 250 µL.

## List of Abbreviations

Alphabetical list of all abbreviations used in the manuscript and Supplementary Materials: AA: Annual Average (EU Environmental Quality Standard); Ace: Acenaphthene; Acen: Acenaphthylene; ANOVA: Analysis of Variance; Ant: Anthracene; B[a]A: Benz[a]anthracene; B[a]P: Benzo[a]pyrene; B[b]Ft: Benzo[b]fluoranthene; B[ghi]P: Benzo[ghi]perylene; B[j]Ft: Benzo[j]fluoranthene; B[k]Ft: Benzo[k]fluoranthene; Chry: Chrysene; CR extract- Concentration Range; DB[a,l]P: Dibenzo[a,l]pyrene; DB[ah]A: Dibenz[a,h]anthracene; DW: Drinking Water; EU: European Union; FLD: Fluorescence Detector; Fln: Fluoranthene; Flu: Fluorene; GC-MS: Gas Chromatography-Mass Spectrometry; HPLC: High-Performance Liquid Chromatography; IARC: International Agency for Research on Cancer; InP: Indeno [1,2,3-cd]pyrene; Kow: octanol–water partition coefficient, LOD: Limit of Detection (Instrumental); LOQ: Limit of Quantification (Instrumental); MAC: Maximum Admissible Concentration (EU); MDL: Method Detection Limit; ME: Matrix Effect; MQL: Method Quantification Limit; Naph: Naphthalene; NCP: Number of calibration points, OFAT: One-Factor-at-a-Time (Optimization Approach); OW: Ocean Water; PAHs/PAH: Polycyclic Aromatic Hydrocarbons; PAD: Photodiode Array Detector; Phe: Phenanthrene; PTFE: Polytetrafluoroethylene (0.22 µm syringe filter material); Pyr: Pyrene; RSD/%RSD: Relative Standard Deviation (%); RW: River Water; SD: Standard Deviation; SPE: Solid-Phase Extraction; THF: Tetrahydrofuran; Tukey’s HSD: Tukey’s Honestly Significant Difference (Post Hoc Test); EPA/EPA: United States Environmental Protection Agency; WWTP: Wastewater Treatment Plant; WWTP-E: Wastewater Treatment Plant Effluent; WWTP-I: Wastewater Treatment Plant Influent.
