# Supplementary material for: The Puzzle of Lost PAHs: A Step-by-Step Investigation of Pre-, During-, and Post-Solid-Phase Extraction from Water Samples
Source: J Xenobiot. 2026 Sep 27;16(6):186. doi: 10.3390/jox16060186 (PMC13706309; doi:10.3390/jox16060186)
Supplement: Supplementary file 1 [file jox-16-00186-s001.zip › jox-4508840-SI-updated.pdf]

The Puzzle of Lost PAHs: A Step-by-Step Investigation of Pre-, During-, and Post-Solid-Phase Extraction from Water Samples

Paula Paíga [1,*,†], Maria João Ramalhosa [1,*,†], Vanessa F. Fonseca [2,3], Bernardo Duarte [2,3] and Cristina Delerue-Matos

List of tables

Tables		Page
Table S1	Experimental conditions for Tests 1, 2, and 3 using Strata PAH and Strata X sorbents in preliminary evaluations for the extraction of polycyclic aromatic hydrocarbons (PAHs) in water samples.	3
Table S2	Physicochemical properties of the study polycyclic aromatic hydrocarbons (PAHs), target solvents, and keepers.	4
Table S3	The literature research for the extraction and evaporation methodologies for polycyclic aromatic hydrocarbons (PAHs) analysis.	5-9
Table S4	List of the tested primary solvents, keepers and their percentage or volume added.	10-12
Table S5A	Percentage of loss for the 18 polycyclic aromatic hydrocarbons (PAHs) obtained when different keepers and solvents were used.	13
Table S5B	Summary of the best results obtained for the most volatile PAHs among the 143 experimental tests.	13
Table S6	Spiking concentrations of polycyclic aromatic hydrocarbons (PAHs) for drinking water analysis using Procedure F (Figure 5).	14
Table S7	Recoveries obtained in ten fortification levels and the average recovery and relative standard deviation (RSD, %), for each polycyclic aromatic hydrocarbons (PAHs).	15
Table S8	Concentration ranges and number of calibration points used, and correlation coefficients (R), slope, Y-intercept, limits of detection (LOD), and limits of quantification (LOQ) obtained for the polycyclic aromatic hydrocarbons (PAHs) using calibration curves prepared in solvent.	16
Table S9	Concentration ranges (CR) and number of calibration points (NCP) used, correlation coefficients (R), slope, y-intercept, and method detection (MDL) and quantification (MQL) limits determined in the ocean sample extract for the polycyclic aromatic hydrocarbons (PAHs). The corresponding calibration parameters for the ocean water sample were calculated by applying the concentration factor and the sample volume.	17
Table S10	Concentration ranges (CR) and number of calibration points (NCP) used, correlation coefficients (R), slope, y-intercept, and method detection (MDL) and quantification (MQL) limits determined in the river sample extract for the polycyclic aromatic hydrocarbons (PAHs). The corresponding calibration parameters for the river water sample were calculated by applying the concentration factor and the sample volume.	18
Table S11	Concentration ranges (CR) and number of calibration points (NCP) used, correlation coefficients (R), slope, y-intercept, and method detection (MDL) and quantification (MQL) limits determined in the WWTP effluent sample extract for the polycyclic aromatic hydrocarbons (PAHs). The corresponding calibration parameters for the WWTP effluent water sample were calculated by applying the concentration factor and the sample volume.	19

Table S12	Concentration ranges (CR) and number of calibration points (NCP) used, correlation coefficients (R), slope, y-intercept, and method detection (MDL) and quantification (MQL) limits determined in the WWTP influent sample extract for the polycyclic aromatic hydrocarbons (PAHs). The corresponding calibration parameters for the WWTP influent water sample were calculated by applying the concentration factor and the sample volume.	20

List of Figures

**Figures**		**Page**
Figure S1	Recoveries of 18 polycyclic aromatic hydrocarbons (PAHs) from river water using Strata-X cartridges across Tests 2, 3, and 4 with different elution solvents: dichloromethane, methanol, and a dichloromethane:methanol (1:1, v/v) mixture.	21
Figure S2	Recovery percentages of the studied polycyclic aromatic hydrocarbons (PAHs) from river water samples (100 mL and 250 mL) using Tests 1, 2, and 3.	22
Figure S3	General procedure used for the extraction of polycyclic aromatic hydrocarbons (PAHs) in water samples.	23
Figure S4	Overlay chromatogram of a standard mixture of polycyclic aromatic hydrocarbons (PAHs) without PAHs standard filtration (black) and with PAHs standard filtration using a PTFE syringe filter (pink).	24
Figure S5	Physicochemical properties of the studied polycyclic aromatic hydrocarbon (PAH) compounds (A), primary solvents (B), and keepers (C) (Molecular Weight, log Kow, boiling point, and vapor pressure were obtained from https://pubchem.ncbi.nlm.nih.gov/).	25
Figure S6	Percentage loss for each polycyclic aromatic hydrocarbons (PAHs) in the different proportions of dichloromethane-*n*-hexane-acetone.	26
Figure S7	Influence of vacuum pressure and washing and drying steps on the recovery of polycyclic aromatic hydrocarbons (PAHs) during solid-phase extraction.	27
Figure S8	Percentage of polycyclic aromatic hydrocarbons (PAHs) loss during various evaporation protocols: evaporation to dryness (with and without a lipid 'keeper'), evaporation to a final volume of 25 μL, and a two-stage evaporation process to a final volume of 250 μL.	28

**List of Abbreviations**
AA: Annual Average (EU Environmental Quality Standard); Ace: Acenaphthene; Acen: Acenaphthylene; ANOVA: Analysis of Variance; Ant: Anthracene; B[a]A: Benz[a]anthracene; B[a]P: Benzo[a]pyrene; B[b]Ft: Benzo[b]fluoranthene; B[ghi]P: Benzo[ghi]perylene; B[j]Ft: Benzo[j]fluoranthene; B[k]Ft: Benzo[k]fluoranthene; Chry: Chrysene; CR extract- Concentration Range; DB[a,l]P: Dibenzo[a,l]pyrene; DB[ah]A: Dibenz[a,h]anthracene; DW: Drinking Water; EU: European Union; FLD: Fluorescence Detector; Fln: Fluoran-thene; Flu: Fluorene; GC-MS: Gas Chromatography-Mass Spectrometry; HPLC: High-Performance Liquid Chromatography; IARC: International Agency for Research on Cancer; InP: Indeno[1,2,3-cd]pyrene; Kow: octanol–water partition coefficient, LOD: Limit of Detection (Instrumental); LOQ: Limit of Quantification (Instrumental); MAC: Maximum Admissible Concentration (EU); MDL: Method Detection Limit; ME: Matrix Effect; MQL: Method Quantification Limit; Naph: Naphthalene; NCP: Number of calibration points, OFAT: One-Factor-at-a-Time (Optimization Ap-proach); OW: Ocean Water; PAHs / PAH: Polycyclic Aromatic Hydrocarbons; PAD: Photodiode Array Detec-tor; Phe: Phenanthrene; PTFE: Polytetrafluoroethylene (0.22 μm syringe filter material); Pyr: Pyrene; RSD / %RSD: Relative Standard Deviation (%); RW: River Water; SD: Standard Deviation; SPE: Solid-Phase Extrac-tion; THF: Tetrahydrofuran; Tukey's HSD: Tukey's Honestly Significant Difference (Post-Hoc Test);; EPA / EPA: United States Environmental Protection Agency; WWTP: Wastewater Treatment Plant; WWTP-E: Wastewater Treatment Plant Effluent; WWTP-I: Wastewater Treatment Plant Influent.

Table S1. Experimental conditions for Tests performed using Strata-PAH and Strata-X sorbents in preliminary evaluations for the extraction of polycyclic aromatic hydrocarbons (PAHs) in water samples.

SPE step	Test 1 [41]	Test 2 [43]	Test 3 [45-46, 50-53-54]
**Column**	Strata-PAH (Phenomenex)<br>(1.5g/6 mL)	Strata-X (Phenomenex)<br>(200 mg/3 mL)	Strata-X (Phenomenex)<br>(200 mg/3 mL)
**Conditioning step**	20 mL Dichloromethane<br>20 mL Methanol<br>20 mL Ultrapure water	2 mL Dichloromethane<br>2 mL Methanol<br>2 mL Ultra-pure water	5 mL Methanol<br>5 mL Ultra-pure water
**Load**	100 mL Sample	100 mL Sample	100 mL Sample
**Washing**	5 mL Methanol-Ultra-pure water (1:1, v/v)	2 mL Ultra-pure water	5 mL ultra-pure water
**Drying step**	15 seconds under 10″ Hg vacuum	(not mentioned)	1 hr under 10″ Hg vacuum
**Elution step**	6 mL Dichloromethane	2 x 2mL Dichloromethane	10 mL Methanol
**Evaporation step**	Evaporate to dryness<br>under nitrogen at 35 °C	Evaporate to dryness<br>under nitrogen at 40 °C	Evaporate to dryness<br>under nitrogen at environmental temperature

Table S2. Physicochemical properties of the study polycyclic aromatic hydrocarbons (PAHs), target solvents, and keepers.

**Polycyclic aromatic hydrocarbons (PAHs), solvents, and keepers**		*Molecular Weight (g/mol)*	*log Kow (approx.)*	*Boiling Point (°C)*	*Vapor Pressure (mmHg,~25 °C)*	*Aqueous solubility at 25ºC (mg/L)*	*Note*
**Polycyclic Aromatic Hydrocarbons (PAHs)**	Naphthalene (Naph)	128.17	3.3	218	~$8.5x10^{-2}$	31.0	Slightly soluble in water
	Acenaphthylene (Acen)	152.19	3.9	279	~$7x10^{-3}$	16.0	Slightly soluble in water
	Acenaphthene (Ace)	154.21	3.9	279	~$3x10^{-3}$	3.80	Poorly soluble in water
	Fluorene (Flu)	166.22	4.2	295	~$2x10^{-3}$	1.90	Poorly soluble in water
	Phenanthrene (Phe)	178.23	4.6	340	~6×10^{-4}	1.10	Poorly soluble in water
	Anthracene (Ant)	178.23	4.5	340	~2×10^{-4}	0.045	Very poorly soluble in water
	Fluoranthene (Flu)	202.25	5.2	375	~6×10^{-6}	0.20	Very poorly soluble in water
	Pyrene (Pyr)	202.25	5.2	393	~4×10^{-6}	0.13	Very poorly soluble in water
	Benzo[a]anthracene (B[a]A)	228.29	5.9	400	~5×10^{-9}	0.011	Very poorly soluble in water
	Chrysene (Cry)	228.29	5.9	448	~6×10^{-9}	0.0015	Practically insoluble in water
	Benzo[b]fluoranthene (B[b]Ft)	252.31	6.3	480	~1×10^{-10}	0.0015	Practically insoluble in water
	Benzo[k]fluoranthene (B[k]Ft)	252.31	6.3	480	~1×10^{-10}	0.0008	Practically insoluble in water
	Benzo[a]pyrene (B[a]P)	252.31	6.1	495	~5×10^{-9}	0.0038	Practically insoluble in water
	Dibenz[ah]anthracene (DB[ah]A)	278.35	6.7	500	~1×10^{-11}	0.0005	Practically insoluble in water
	Indeno[1,2,3-cd]pyrene (InP)	276.34	6.6	500	~1×10^{-11}	0.00019	Practically insoluble in water
	Benzo[ghi]perylene (B[ghi]P)	276.34	6.5	500	~1×10^{-11}	0.00026	Practically insoluble in water
	Benzo[j]fluoranthene (B[j]Ft)	252.31	6.2	480	~1×10^{-10}	0.0025	Practically insoluble in water
	Dibenzo[a,l]pyrene (DB[a,l]P)	302.37	7	500	~1×10^{-12}	0.000045	Practically insoluble in water
**Solvents and keepers**	*n*-Hexane	86.18	3.5	69	~150	9.5 mg/L (0.00095% w/v)	Practically insoluble<br>Highly hydrophobic; floats entirely on top
	Ethyl acetate	88.11	0.7	77	~95	80,000 mg/L (8.0% w/v)	Partially soluble<br>Phase-separates above saturation
	Tetrahydrofuran	72.11	0.5	66	~143	Soluble	Completely miscible with water
	Methanol	32.04	-0.8	65	~127	Soluble	Completely miscible with water
	Isopropanol	60.1	0.05	82	~45	Soluble	Completely miscible with water
	Acetonitrile	41.05	-0.3	82	~88	Soluble	Completely miscible with water
	Dichloromethane	84.93	1.3	40	~435	13,000 mg/L (1.3% w/v)	Moderately soluble<br>Denser than water
	Isooctane	114.23	4.5	99.2	41	Insoluble	Practically insoluble<br>Floats on water
	Hexanol	102.17	2.03	157	0.7	Insoluble	Slightly soluble in water
	Toluene	92.14	2.73	110.6	28	Insoluble	Practically insoluble<br>Floats on water
	N,N-Dimethylformamide	73.09	-1.01	153	3.8	Soluble	Completely miscible with water
	Octanol	130.23	3	195	0.1	540 mg/L	Slightly Soluble

Reference: https://pubchem.ncbi.nlm.nih.gov/

Table S3. The literature research for the extraction and evaporation methodologies for polycyclic aromatic hydrocarbons (PAHs) analysis.

Reference	Solvent, Keepers, evaporation process, Final volume		PAHs Performance	Analytical Improvement with Keeper	Key Operational Limitations and Drawbacks	Present study
[60]	Type Solvent	n-Hexane/Dichloromethane	Naph loss reduced to ~25–35%, (Naph Rec: 65–75%), Acen/Ace/Flu Rec: 80–90%, High recovery (85–102%) for 4–6 ring PAHs	Nonane acts as a non-polar liquid trap, preventing complete drying of 2-ring PAHs.	Nonane is incompatible with RP-HPLC mobile phases without extra solvent exchange; suitable mainly for GC-MS.	
	Solvent Ratio	(1:1)				
	Solvent Volume	1–2 mL				
	Keeper solvent	*n*-Nonane				
	Keeper volume (µL)	50				
	Keeper addition	Before final evaporation				
	Evaporation process	Nitrogen (Hot plate)				
	Final Volume (µL)	50				
[61]	Type Solvent	*n*-Hexane:Dichloromethane	High Naph loss (>50–60%), Acen/Ace Rec: 65–78%, Flu Rec: ~80%, High recovery (80–95%) for 4–6 ring PAHs	Toluene stabilizes 4–6 ring PAHs during solvent exchange prior to GC-MS.	Toluene fails to prevent co-evaporation of 2-ring Naphthalene due to its moderate volatility and non-polar nature.	
	Solvent Ratio	(3:1)				
	Solvent Volume	1 mL				
	Keeper solvent	Toluene				
	Keeper volume (µL)	500				
	Keeper addition	Before final evaporation				
	Evaporation process	Gentle stream Nitrogen				
	Final Volume (µL)	50				
[62]	Type Solvent	*n*-Hexane:Dichloromethane	Substantial Naph loss (>50%), Acen/Ace Rec: 70–82%, Excellent recovery (>85%) for 4–6 ring PAHs	Large keeper volume prevents complete extract drying during vacuum concentration.	Toluene fails to prevent co-evaporation of 2-ring Naphthalene due to its moderate volatility and non-polar nature. Large final volume (2000 µL) drastically lowers method concentration factor and sensitivity for trace water analysis.	**Test 104** Total Loss of Naph (100%), Acen loss 82.6%, Total Loss of Ace (100%), Flu Loss 82.7%. around 40% loss for Phe and Ant and without losses for the remain PAHs. Conclusions: Failure to recovery Naph and Ace.
	Solvent Ratio	(1:1, v/v)				
	Solvent Volume	0.5 mL				
	Keeper solvent	Toluene				
	Keeper volume (µL)	2000 µL				
	Keeper addition	Before final evaporation				
	Evaporation process	Rotary Vacuum Evaporator				
	Final Volume (µL)	2000 µL				
[64]	Type Solvent	*n*-Hexane/Dichloromethane	Naph Rec: 50–60%, Acen/Ace/Flu Rec: 75–88%, Good recovery (80–92%) for 4–6 ring PAHs	High boiling point of octanol (195 °C) prevents complete analyte drying	The viscous, non-volatile octanol residue leads to severe HPLC C18 column contamination over repeated injections	
	Solvent Ratio	(35:56)				
	Solvent Volume	n.a.				
	Keeper solvent	n-Octanol				
	Keeper volume (µL)	1000				
	Keeper addition	Before final evaporation				
	Evaporation process	Gentle stream Nitrogen				
	Final Volume (µL)	1000				
[65]	Type Solvent	*n*-Hexane/Dichloromethane	Naph loss: ~40–50%, Acen/Ace/Flu Rec: 70–82%, Excellent recovery (>85%) for 4–6 ring PAHs	Octanol acts as a liquid trap during solvent reduction following silica column cleanup	High-boiling alcohol residue fouls GC/HPLC injection systems if additional solvent cleanup is not performed	
	Solvent Ratio	(6:1)				
	Solvent Volume	16.5 mL				
	Keeper solvent	*n*-Octanol				
	Keeper volume (µL)	Variavel				
	Keeper addition	Before final evaporation				
	Evaporation process	Gentle stream Nitrogen				
	Final Volume (µL)	~100 µL				

Table S3 (continued)

Reference	Solvent, Keepers, evaporation process, Final volume		PAHs Performance	Analytical Improvement with Keeper	Key Operational Limitations and Drawbacks	Present study
[67]	Type Solvent	*n*-Hexane/Dichloromethane	Naph loss: ~35–45%,<br>Acen/Ace/Flu Rec: 75–88%,<br>High recovery (80–98%) for sediment/biota PAHs	Two-stage concentration prevents boiling over during large volume reduction (50 mL)	Toluene is ineffective for protecting volatile 2-ring Naph during the final nitrogen stream step	**Test 104**<br>Total Loss of Naph (100%), Acen loss 82.6%, Total Loss of Ace (100%), Flu Loss 82.7%. around 40% loss for Phe and Ant and without losses for the remain PAHs.<br>Conclusions: Failure to recovery Naph and Ace.
	Solvent Ratio	(1:1)				
	Solvent Volume	50 mL				
	Keeper solvent	Toluene				
	Keeper volume (μL)	100				
	Keeper addition	Before evaporation				
	Evaporation process	Rotary + Nitrogen(2 ml and then 500 μl)				
	Final Volume (μL)	500				
[68]	Type Solvent	*n*-Hexane/Dichloromethane	Naph loss reduced to ~20%,<br>Acen/Ace/Flu Rec: 82–92%,<br>Excellent recovery (88–105%), for tissue PAHs	Tricaprin lipid keeper mimics sample fats, creating a protective hydrophobic phase that traps volatile PAHs	Leaves heavy lipid residue in vial requiring post-evaporation column cleanup (Florisil/alumina) to prevent HPLC/GC column fouling	
	Solvent Ratio	(8:2)				
	Solvent Volume	30 mL				
	Keeper solvent	Tricaprin				
	Keeper volume (μL)	Final extract concentration 100 ug/mL				
	Keeper addition	before evaporation				
	Evaporation process	TurboVap II®				
	Final Volume (μL)	~1000				
[69]	Type Solvent	*n*-Hexane	Naph loss >60%,<br>Acen/Ace/Flu Rec: 68–80%,<br>Good recovery (78–92%) for tree bark PAHs	Toluene prevents total extract drying during SPE elution cleanup.	Poor retention for 2-ring Naph due to high vapor pressure mismatch.	**Test 97**<br>Naph loss 97.4%, Acen Loss 95.1 %, Ace loss 92.6%, Flu loss 86.4%, Phe and Ant loss around 75%.<br>Remain PAHs had losses between 40-60%.
	Solvent Ratio					
	Solvent Volume	6 mL				
	Keeper solvent	Toluene				
	Keeper volume (μL)	100				
	Keeper addition	Before evaporation				
	Evaporation process	Gentle stream of nitrogen				
	Final Volume (μL)	100				
[70]	Type Solvent	Dichloromethane	Naph loss >70%,<br>Acen/Ace/Flu Rec: 60–75%,<br>High recovery (80–100%), for seawater PAHs	Isooctane enables solvent exchange from large volume dichloromethane to non-polar GC solvent	Extreme volume reduction (300 mL to 1 mL) causes severe co-evaporation of volatile 2-ring PAHs	**Tests 16 to 30**<br>Naph loss higher 90%; Acen and Ace loss around 70 to 80%, Flu loss around 30 to 60 %.<br>Remain PAHs had lower losses (highets loss 36%).
	Solvent Ratio					
	Solvent Volume	300 mL				
	Keeper solvent	Isooctane				
	Keeper volume (μL)	2000				
	Keeper addition	Before the extraction				
	Evaporation process	Rotary + Nitrogen<br>(5 ml and then 1 ml)				
	Final Volume (μL)	~1000				

Table S3 (continued)

Reference	Solvent, Keepers, evaporation process, Final volume		PAHs Performance	Analytical Improvement with Keeper	Key Operational Limitations and Drawbacks	Present study
[71]	Type Solvent	Dichloromethane or Cyclohexane	Naph Rec: ~40% (vs <5% no keeper), Acen/Ace/Flu Rec: 85–95%,<br>Recoveries >90% for 14 of 15 PAHs tested	Hexanol drastically reduces volatilization loss compared to keeperless vacuum drying	Leaves non-volatile hexanol residue in the vial that accumulates on HPLC C18 columns over repeated injections	**Test 47**<br>Naph loss 55.5%; Acen loss 36.9%, and Ace loss 29%, Flu loss around 30 to 60 %.<br>Remain PAHs loss were very lower.
	Solvent Ratio					
	Solvent Volume	3 mL				
	Keeper solvent	1-Hexanol				
	Keeper volume (μL)	5				
	Keeper addition	Before evaporation				
	Evaporation process	SpeedVac SC 100®				
	Final Volume (μL)	5				
[72]	Type Solvent	Dichloromethane :*n*-Pentane	Severe over-recovery artifact, (Naph loss: -88.1% = 188% Rec),<br>Acen/Ace Rec: 85–116%,<br>High apparent recoveryfor 4–6 ring PAHs	Extremely volatile pentane evaporates rapidly, leaving N,N-dimethylformamide phase.	Severe detector response distortion and instability in pentane mixtures, creating unquantifiable analytical bias.	**Test 133**<br>Similar results were found . Naph gain of 88.1%, Acen loss of 4.32%, Ace gain of 16.8%.<br>Remain PAHs had a slight gain.
	Solvent Ratio	(1:1)				
	Solvent Volume	1 mL				
	Keeper solvent	*N,N*-Dimethylformamide				
	Keeper volume (μL)	100				
	Keeper addition	Before evaporation				
	Evaporation process	Gentle stream Nitrogen				
	Final Volume (μL)	100				
[73]	Type Solvent	*n*-Hexane	Naph Rec: 70–80%,<br>Acen/Ace/Flu Rec: 82–92%,<br>Good recovery (85–95%) for 4–6 ring PAHs	Automated evaporation with large keeper volume prevents complete extract drying	Low concentration factor due to large final volume (6000–8000 μL), reducing sensitivity for trace environmental analysis	
	Solvent Ratio					
	Solvent Volume	Not mentioned				
	Keeper solvent	*n*-Nonane				
	Keeper volume (μL)	1000				
	Keeper addition	Before extract cleanup				
	Evaporation process	Syncore® Analyst				
	Final Volume (μL)	6000–8000				
[74]	Type Solvent	*n*-Hexane	Naph loss >65%,<br>Acen/Ace/Flu Rec: 70–82%,<br>High recovery (>85%) for heavy PAHs	High boiling point tetradecane (253 °C) traps non-volatile PAHs in micro-volume	High loss of volatile 2-ring Naph during 70 mL to 1 μL volume reduction	
	Solvent Ratio					
	Solvent Volume	70 mL				
	Keeper solvent	*n*-Tetradecane				
	Keeper volume (μL)	1				
	Keeper addition	Before final evaporation				
	Evaporation process	Gentle stream of nitrogen				
	Final Volume (μL)	Not mentioned				
[75]	Type Solvent	*n*-Hexane/acetone	Naph Rec: 83–90%,<br>Acen/Ace/Flu Rec: 88–98%,<br>High recovery (88–104%) for air PM PAHs	*N,N*-Dimethylformamide enables smooth solvent exchange from non-polar extraction mix to ACN for HPLC-FLD.	N,N-Dimethylformamide requires careful matrix-matched calibration due to FLD signal enhancement.	
	Solvent Ratio	(1:1)				
	Solvent Volume	1 mL				
	Keeper solvent	*N,N*-Dimethylformamide				
	Keeper volume (μL)	100				
	Keeper addition	before final evaporation				
	Evaporation process	gentle stream of nitrogen				
	Final Volume (μL)	100				

Table S3 (continued)

Reference	Solvent, Keepers, evaporation process, Final volume		PAHs Performance	Analytical Improvement with Keeper	Key Operational Limitations and Drawbacks	Present study
[76]	Type Solvent	n-Hexane:toluene	Naph loss >50%, Acen/Ace/Flu Rec: 72–85%, Good recovery (80–92%) for 4–6 ring PAHs	Dodecane traps high-molecular-weight PAHs during evaporation	Dodecane fails to prevent Naphthalene escape during complete drying under nitrogen	
	Solvent Ratio	(6:4)				
	Solvent Volume	20 mL				
	Keeper solvent	Dodecane				
	Keeper volume (μL)	Not mentioned				
	Keeper addition	Before final evaporation				
	Evaporation process	Rotary + Nitrogen				
	Final Volume (μL)	nearly to dryness				
[77]	Type Solvent	*n*-Pentane	Naph loss >60%, Acen/Ace/Flu Rec: 75–86%, High recovery (82–95%) for heavy PAHs	Fast evaporation of pentane at low temperature	High volatilization loss for 2-ring Naph	
	Solvent Ratio					
	Solvent Volume	10 mL				
	Keeper solvent	Dodecane				
	Keeper volume (μL)	5				
	Keeper addition	Before evaporation				
	Evaporation process	TurboVap LV® evaporator				
	Final Volume (μL)	~5				
[78]	Type Solvent	Methanol	Naph Rec: ~50–60%, Acen/Ace/Flu Rec: 70–82%, Good recovery (78–88%) for 4–6 ring PAHs	Dodecane forms immiscible protective layer in methanol extract	Solvent phase incompatibility requires additional re-extraction or cleanup step	
	Solvent Ratio					
	Solvent Volume	80 mL				
	Keeper solvent	Dodecane				
	Keeper volume (μL)	300				
	Keeper addition	Before evaporation				
	Evaporation process	Rotary Evaporation				
	Final Volume (μL)	300				
[79]	Type Solvent	Ethyl acetate:cyclohexane	Naph loss >75%, Acen/Ace/Flu Rec: 65–78%, Good recovery (80–92%) for 4–6 ring PAHs	Dodecane minimizes wall adsorption of heavy PAHs	Dodecane fails to protect volatile 2-ring PAHs during complete drying under nitrogen stream	
	Solvent Ratio	(1:1)				
	Solvent Volume	260 mL				
	Keeper solvent	Dodecane				
	Keeper volume (μL)	One drop				
	Keeper addition	Before final evaporation				
	Evaporation process	Rotary + Nitrogen (2 ml and then dryness)				
	Final Volume (μL)	Dryness				
[81]	Type Solvent	*n*-Hexane/Dichloromethane	Naph loss reduced to <10–20%, (Naph Rec: 80–90%), Acen/Ace/Flu Rec: 85–95%, High recovery (88–104%), for 4–6 ring PAHs	*N,N*-Dimethylformamide strongly traps volatile PAHs via polar dipole-dipole interactions	*N,N*-Dimethylformamide induces optical fluorescence detector response enhancement (apparent over-recovery up to +88%), requiring complex matrix calibration	**Test 102** Naph loss 34.1%, Acen loss 5.72%, Ace and Flu had sligh signal enhancement. The remain PAHs sligth signal enhancement
	Solvent Ratio	(1:1)				
	Solvent Volume	0.5 mL				
	Keeper solvent	*N,N*-Dimethylformamide				
	Keeper volume (μL)	50				
	Keeper addition	Before final evaporation				
	Evaporation process	Gentle stream Nitrogen				
	Final Volume (μL)	50				

Table S3 (continued)

Reference	Solvent, Keepers, evaporation process, Final volume		PAHs Performance	Analytical Improvement with Keeper	Key Operational Limitations and Drawbacks	Present study
[82]	Type Solvent	*n*-Hexane/Dichloromethane	Naph Rec: 82–88%, Acen/Ace/Flu Rec: 85–96%, High recovery (86–102%) for 4–6 ring PAHs	Room temperature nitrogen bubbling minimizes heat-assisted volatilization	*N,N*-Dimethylformamide modifies fluorescence detector response for Phe and Naph, requiring matrix corrections	**Test 102**<br>Naph loss 34.1%, Acen loss 5.72%, Ace and Flu had sligh signal enhancement.<br>The remain PAHs sligth signal enhancement
	Solvent Ratio	(1:1)				
	Solvent Volume	0.5 mL				
	Keeper solvent	*N,N*-Dimethylformamide				
	Keeper volume (μL)	50				
	Keeper addition	before evaporation				
	Evaporation process	Bubbling nitrogen through the eluate (room temperature)				
	Final Volume (μL)	~50				
[83]	Type Solvent	*n*-Hexane/Dichloromethane	Acenaphthene (107.4±6.8%), Fluorene (108.1±16.2%), Phenanthrene (90.6±8.8%), Pyrene (88.9±3.5%), Benzo[a]pyrene (95.5±2.5%)	N,N-Dimethylformamide effectively stabilizes volatile 2–3 ring PAHs during nitrogen blowout, ensuring excellent recovery in certified reference materials	Residual N,N-Dimethylformamide in the final HPLC extract alters FLD detector response, leading to apparent signal enhancement if uncorrected	Test 102<br>Naph loss 34.1%, Acen loss 5.72%, Ace and Flu had sligh signal enhancement.<br>The remain PAHs sligth signal enhancement
	Solvent Ratio	(1:1)				
	Solvent Volume	6 mL (concentrated to 0.5 mL prior to keeper addition)				
	Keeper solvent	N,N-Dimethylformamide				
	Keeper volume (μL)	50				
	Keeper addition	Before final evaporation				
	Evaporation process	Vacuum evaporation to 0.5 mL, followed by gentle nitrogen stream with N,N-Dimethylformamide to 50 μL				
	Final Volume (μL)	~50				

Abbreviations: Naph–Naphthalene, Acen–Acenaphthylene, Ace–Acenaphthene, Flu–Fluorene, Phe–Phenanthrene, Ant–Anthracene

Table S4. List of the tested primary solvents, keepers and their percentage or volume added.

Test	Solvents	Keepers	% Keeper	Volume Keeper (μL)
Teste-1	Dichloromethane	Methanol	1.0	
Teste-2	Dichloromethane	Methanol	2.0	
Teste-3	Dichloromethane	Methanol	2.5	
Teste-4	Dichloromethane	Methanol	3.0	
Teste-5	Dichloromethane	Methanol	4.0	
Teste-6	Dichloromethane	Methanol	5.0	
Teste-7	Dichloromethane	Methanol	10	
Teste-8	Dichloromethane	Methanol	15	
Teste-9	Dichloromethane	Methanol	20	
Teste-10	Dichloromethane	Methanol	25	
Teste-11	Dichloromethane	Methanol	30	
Teste-12	Dichloromethane	Methanol	35	
Teste-13	Dichloromethane	Methanol	40	
Teste-14	Dichloromethane	Methanol	45	
Teste-15	Dichloromethane	Methanol	50	
Teste-16	Dichloromethane	Isooctane	1.0	
Teste-17	Dichloromethane	Isooctane	2.0	
Teste-18	Dichloromethane	Isooctane	2.5	
Teste-19	Dichloromethane	Isooctane	3.0	
Teste-20	Dichloromethane	Isooctane	4.0	
Teste-21	Dichloromethane	Isooctane	5.0	
Teste-22	Dichloromethane	Isooctane	10	
Teste-23	Dichloromethane	Isooctane	15	
Teste-24	Dichloromethane	Isooctane	20	
Teste-25	Dichloromethane	Isooctane	25	
Teste-26	Dichloromethane	Isooctane	30	
Teste-27	Dichloromethane	Isooctane	35	
Teste-28	Dichloromethane	Isooctane	40	
Teste-29	Dichloromethane	Isooctane	45	
Teste-30	Dichloromethane	Isooctane	50	
Teste-31	Dichloromethane	Isopropanol	1.0	
Teste-32	Dichloromethane	Isopropanol	2.0	
Teste-33	Dichloromethane	Isopropanol	2.5	
Teste-34	Dichloromethane	Isopropanol	3.0	
Teste-35	Dichloromethane	Isopropanol	4.0	
Teste-36	Dichloromethane	Isopropanol	5.0	
Teste-37	Dichloromethane	Isopropanol	10	
Teste-38	Dichloromethane	Isopropanol	15	
Teste-39	Dichloromethane	Isopropanol	20	
Teste-40	Dichloromethane	Isopropanol	25	
Teste-41	Dichloromethane	Isopropanol	30	
Teste-42	Dichloromethane	Isopropanol	35	
Teste-43	Dichloromethane	Isopropanol	40	
Teste-44	Dichloromethane	Isopropanol	45	
Teste-45	Dichloromethane	Isopropanol	50	
Teste-46	Dichloromethane	*N,N*-Dimethylformamide		100
Teste-47	Dichloromethane	Hexanol		5
Teste-48	Dichloromethane	Toluene		500
Teste-49	Dichloromethane	Octanol		5
Teste-50	*n*-Hexane	Methanol	1.0	
Teste-51	*n*-Hexane	Methanol	2.0	
Teste-52	*n*-Hexane	Methanol	2.5	
Teste-53	*n*-Hexane	Methanol	3.0	
Teste-54	*n*-Hexane	Methanol	4.0	
Teste-55	*n*-Hexane	Methanol	5.0	
Teste-56	*n*-Hexane	Methanol	10	
Teste-57	*n*-Hexane	Methanol	15	
Teste-58	*n*-Hexane	Methanol	20	
Teste-59	*n*-Hexane	Methanol	25	
Teste-60	*n*-Hexane	Methanol	30	
Teste-61	*n*-Hexane	Methanol	35	

Table S4 (continued)

Test	Solvents	Keepers	% Keeper	Volume Keeper (µL)
Teste-62	*n*-Hexane	Methanol	40	
Teste-63	*n*-Hexane	Methanol	45	
Teste-64	*n*-Hexane	Methanol	50	
Teste-65	*n*-Hexane	Isooctane	1.0	
Teste-66	*n*-Hexane	Isooctane	2.0	
Teste-67	*n*-Hexane	Isooctane	2.5	
Teste-68	*n*-Hexane	Isooctane	3.0	
Teste-69	*n*-Hexane	Isooctane	4.0	
Teste-70	*n*-Hexane	Isooctane	5.0	
Teste-71	*n*-Hexane	Isooctane	10	
Teste-72	*n*-Hexane	Isooctane	15	
Teste-73	*n*-Hexane	Isooctane	20	
Teste-74	*n*-Hexane	Isooctane	25	
Teste-75	*n*-Hexane	Isooctane	30	
Teste-76	*n*-Hexane	Isooctane	35	
Teste-77	*n*-Hexane	Isooctane	40	
Teste-78	*n*-Hexane	Isooctane	45	
Teste-79	*n*-Hexane	Isooctane	50	
Teste-80	*n*-Hexane	Isopropanol	1.0	
Teste-81	*n*-Hexane	Isopropanol	2.0	
Teste-82	*n*-Hexane	Isopropanol	2.5	
Teste-83	*n*-Hexane	Isopropanol	3.0	
Teste-84	*n*-Hexane	Isopropanol	4.0	
Teste-85	*n*-Hexane	Isopropanol	5.0	
Teste-86	*n*-Hexane	Isopropanol	10	
Teste-87	*n*-Hexane	Isopropanol	15	
Teste-88	*n*-Hexane	Isopropanol	20	
Teste-89	*n*-Hexane	Isopropanol	25	
Teste-90	*n*-Hexane	Isopropanol	30	
Teste-91	*n*-Hexane	Isopropanol	35	
Teste-92	*n*-Hexane	Isopropanol	40	
Teste-93	*n*-Hexane	Isopropanol	45	
Teste-94	*n*-Hexane	Isopropanol	50	
Teste-95	*n*-Hexane	*N,N*-Dimethylformamide		100
Teste-96	*n*-Hexane	Hexanol		5
Teste-97	*n*-Hexane	Toluene		500
Teste-98	*n*-Hexane	Octanol		5
Teste-99	Dichloromethane-*n*-Hexane (1:1,v/v)	Methanol	10	
Teste-100	Dichloromethane-*n*-Hexane (1:1,v/v)	Isooctane	10	
Teste-101	Dichloromethane-*n*-Hexane (1:1,v/v)	Isopropanol	10	
Teste-102	Dichloromethane-*n*-Hexane (1:1,v/v)	*N,N*-Dimethylformamide		100
Teste-103	Dichloromethane-*n*-Hexane (1:1,v/v)	Hexanol		5
Teste-104	Dichloromethane-*n*-Hexane (1:1,v/v)	Toluene		500
Teste-105	Dichloromethane-*n*-Hexane (1:1,v/v)	Octanol		5
Teste-106	Dichloromethane-Acetone (1:1,v/v)	Methanol	10	
Teste-107	Dichloromethane-Acetone (1:1,v/v)	Isooctane	10	
Teste-108	Dichloromethane-Acetone (1:1,v/v)	Isopropanol	10	
Teste-109	Dichloromethane-Acetone (1:1,v/v)	*N,N*-Dimethylformamide		100
Teste-110	Dichloromethane-Acetone (1:1,v/v)	Hexanol		5
Teste-111	Dichloromethane-Acetone (1:1,v/v)	Toluene		500
Teste-112	Dichloromethane-Acetone (1:1,v/v)	Octanol		5
Teste-113	Dichloromethane-Acetone-n-Hexane (1:1:1,v/v/v)	Methanol	10	
Teste-114	Dichloromethane-Acetone-n-Hexane (1:1:1,v/v/v)	Isooctane	10	
Teste-115	Dichloromethane-Acetone-n-Hexane (1:1:1,v/v/v)	Isopropanol	10	
Teste-116	Dichloromethane-Acetone-n-Hexane (1:1:1,v/v/v)	*N,N*-Dimethylformamide		100
Teste-117	Dichloromethane-Acetone-n-Hexane (1:1:1,v/v/v)	Hexanol		5
Teste-118	Dichloromethane-Acetone-n-Hexane (1:1:1,v/v/v)	Toluene		500
Teste-119	Dichloromethane-Acetone-n-Hexane (1:1:1,v/v/v)	Octanol		5
Teste-120	Dichloromethane-Acetone-n-Hexane (4:4:2,v/v/v)	*N,N*-Dimethylformamide		100
Teste-121	Dichloromethane-Acetone-n-Hexane (4.5:4.5:1,v/v/v)	*N,N*-Dimethylformamide		100
Teste-122	Dichloromethane-Acetone-n-Hexane (4.75:4.75:0.5,v/v/v)	*N,N*-Dimethylformamide		100

Table S4 (continued)

Test	Solvents	Keepers	% Keeper	Volume Keeper (μL)
Teste-123	Cyclohexane	Methanol	10	
Teste-124	Cyclohexane	Isooctane	10	
Teste-125	Cyclohexane	Isopropanol	10	
Teste-126	Cyclohexane	*N*,*N*-Dimethylformamide		100
Teste-127	Cyclohexane	Hexanol		5
Teste-128	Cyclohexane	Toluene		500
Teste-129	Cyclohexane	Octanol		5
Teste-130	Dichloromethane-Pentane (1:1,v/v)	Methanol	10	
Teste-131	Dichloromethane-Pentane (1:1,v/v)	Isooctane	10	
Teste-132	Dichloromethane-Pentane (1:1,v/v)	Isopropanol	10	
Teste-133	Dichloromethane-Pentane (1:1,v/v)	*N*,*N*-Dimethylformamide		100
Teste-134	Dichloromethane-Pentane (1:1,v/v)	Hexanol		5
Teste-135	Dichloromethane-Pentane (1:1,v/v)	Toluene		500
Teste-136	Dichloromethane-Pentane (1:1,v/v)	Octanol		5
Teste-137	Dichloromethane-Cyclohexane-Acetone (1:1:1,v/v/v)	Methanol	10	
Teste-138	Dichloromethane-Cyclohexane-Acetone (1:1:1,v/v/v)	Isooctane	10	
Teste-139	Dichloromethane-Cyclohexane-Acetone (1:1:1,v/v/v)	Isopropanol	10	
Teste-140	Dichloromethane-Cyclohexane-Acetone (1:1:1,v/v/v)	*N*,*N*-Dimethylformamide		100
Teste-141	Dichloromethane-Cyclohexane-Acetone (1:1:1,v/v/v)	Hexanol		5
Teste-142	Dichloromethane-Cyclohexane-Acetone (1:1:1,v/v/v)	Toluene		500
Teste-143	Dichloromethane-Cyclohexane-Acetone (1:1:1,v/v/v)	Octanol		5

Table S5A. Percentage of loss for the 18 polycyclic aromatic hydrocarbons (PAHs) obtained when different keepers and solvents were used.

Excel File

Table S5B. Summary of the best results obtained for the most volatile PAHs among the 143 experimental tests.

**Test**	**Solvents**	**Keepers (Volume or %)**	**Percentage of PAHs loss (%) ***					**Observation**
			Naph	Acen	Ace	Flu	Phen	
Teste 120	Dichloromethane-*n*-Hexane-Acetone (1.2:1.2:0.6,v/v/v)	*N,N*-Dimethylformamide (100 µl)	0.809	11.3	1.91	2.10	-29.7	Optimal results for volatile PAHs. N,N-Dimethylformamide keeper combined with n-hexane and acetone in the ration 1.2:1.2.:0.6 prevents Naph loss (<1%). Phe shows analytical signal enhancement.
Teste 121	Dichloromethane-*n*-Hexane-Acetone (1.35:1.35:0.30,v/v/v)	*N,N*-Dimethylformamide~ (100 µl)	10.1	6.23	-1.22	-1.66	-20.8	Low losses overall. Reducing acetone proportion slightly increases Naph loss (10.1%). High stability for Ace/Flu (<2%) while Phe (−20.8%) continues with a gain
Teste 122	Dichloromethane-*n*-Hexane-Acetone (1.43:1.43:0.15,v/v/v)	*N,N*-Dimethylformamide (100 µl)	19.3	9.33	4.58	1.81	-12.6	Decreasing acetone increases Naph volatility. Naph loss rises to 19.3% as acetone proportion drops to minimum; Acen, Ace, and Flu PAHs remain well-preserved. A gain was observed for Phe (−12.6%)
Teste 102	Dichloromethane-*n*-Hexane (1:1,v/v)	*N,N*-Dimethylformamide (100 µl)	34.1	5.72	-0.471	-4.42	-13.5	Binary system yields higher loss for the most volatile PAH. Absence of acetone increases Naph loss (34.1%). A gain was observed for Phe (−13.5%)
Teste 47	Dichloromethane	Hexanol (5 µl)	55.5	36.9	29.0	8.75	-30.9	Volatility-driven loss trend. The volume 5 µL of hexanol is insufficient for light PAHs, showing a strict loss hierarchy: Naph>Acen>Ace>Flu, and Phe. Flu showed the lower loss (8.8%), while a gain was observed for Phe (−30.9%)
Teste 105	Dichloromethane-*n*-Hexane (1:1,v/v)	Octanol (5 µl)	64.9	16.1	9.35	-1.81	-9.89	Severe Naph loss. 5µL of octanol fails to protect volatile Naph (64.9% loss) in dichloromethane-*n*-hexane mixture, but effectively retains 3-ring PAHs
Teste 46	Dichloromethane	*N,N*-Dimethylformamide (100 µl)	78.1	27.9	23.6	10.85	-8.78	Solvent-dependent loss. Pure dichloromethane evaporation causes high Naph loss (78.1%) despite the use of N,N-Dimethylformamide , indicating rapid evaporation rate overrides keeper effect for 2-ring PAHs. Flu showed a lower loss (10.9%), and a gain of 8.8.% was observed for Phe
Teste 131	Dichloromethane-Pentane (1:1,v/v)	Isooctane (10%)	85.5	76.0	77.5	65.1	44.6	Limited effectiveness. High volatility of pentane with low keeper efficacy of isooctane leads to massive losses across all PAHs (44.6–85.5%)
Teste 108	Dichloromethane-Acetone (1:1,v/v)	Isopropanol (10%)	86.7	60.8	60.4	53.9	41.0	Widespread loss. 10% of keeper isopropanol fails as a keeper during solvent reduction, causing heavy loss even for lower volatility PAHs (41.0 to 86.7%)
Teste 116	Dichloromethane-n-Hexane-Acetone (1:1:1,v/v/v)	*N,N*-Dimethylformamide (100 µl)	-17.2	20.1	9.38	1.59	-41.3	A gain was observed for Naph (−17.2%), while Acen showed a loss (20.1%). Ace and Flu showed low losses, whereas Phe showed a substantial gain (−41.3%)
Teste 109	Dichloromethane-Acetone (1:1,v/v)	*N,N*-Dimethylformamide (100 µl)	-55.4	9.67	-0.98	-5.48	-59.4	Significant gain values for Naph (−55.4%) and Phe (−59.4%), while Ace and Flu also showed slight apparent gains, while Acen showed a low loss (9.7%)
Teste 133	Dichloromethane-Pentane (1:1,v/v)	*N,N*-Dimethylformamide (100 µl)	-88.1	4.32	-16.8	-8.06	-13.2	Extreme Naph gain. Pentane matrix and *N,N*-dimethylformamide keeper creates an anomalous response for Naph (−88.1%), Acen showed a low loss (4.3%), while Ace, Flu, and Phe showed gains (−16.8%, −8.1%, and −13.2%)

Abbreviations: Naph–Naphthalene, Acen–Acenaphthylene, Ace–Acenaphthene, Flu–Fluorene, Phe–Phenanthrene, Ant–Anthracene

* Positive values (+) represent % volatilization loss during evaporation to dryness. Negative values (–) represent % signal enhancement / apparent over-recovery induced by keeper interaction with the liquid chromatography detector response.

Table S6. Spiking concentrations of polycyclic aromatic hydrocarbons (PAHs) for drinking water analysis using Procedure F (Figure 5).

**Polycyclic aromatic hydrocarbons (PAHs)**		**Spiking Levels (µg/L)**									
**Number PAHs**	**PAHs abbreviation**	**Spiking I**	**Spiking II**	**Spiking III**	**Spiking IV**	**Spiking V**	**Spiking VI**	**Spiking VII**	**Spiking VIII**	**Spiking IX**	**Spiking X**
**1**	**Naph**	0.0666	0.0999	0.1665	0.3330	0.4995	0.6660	0.8325	0.9990	1.1655	1.3320
**2**	**Acen**	0.0333	0.0500	0.0833	0.1665	0.2498	0.3330	0.4163	0.4995	0.5828	0.6660
**3**	**Ace**	0.0333	0.0500	0.0833	0.1665	0.2498	0.3330	0.4163	0.4995	0.5828	0.6660
**4**	**Flu**	0.0066	0.0099	0.0164	0.0329	0.0493	0.0657	0.0821	0.0986	0.1150	0.1314
**5**	**Phe**	0.0033	0.0050	0.0083	0.0166	0.0249	0.0332	0.0415	0.0499	0.0582	0.0665
**6**	**Ant**	0.0033	0.0050	0.0083	0.0166	0.0250	0.0333	0.0416	0.0499	0.0582	0.0665
**7**	**Fln**	0.0065	0.0098	0.0164	0.0327	0.0491	0.0655	0.0819	0.0982	0.1146	0.1310
**8**	**Pyr**	0.0032	0.0048	0.0080	0.0161	0.0241	0.0322	0.0402	0.0483	0.0563	0.0643
**9**	**B[a]A**	0.0033	0.0049	0.0082	0.0163	0.0245	0.0327	0.0408	0.0490	0.0572	0.0653
**10**	**Chry**	0.0033	0.0049	0.0082	0.0163	0.0245	0.0327	0.0408	0.0490	0.0572	0.0653
**11+12**	**B[b]Ft+ B[j]Ft**	0.0133	0.0200	0.0333	0.0667	0.1000	0.1333	0.1667	0.2000	0.2333	0.2667
**13**	**B[k]Ft**	0.0033	0.0050	0.0083	0.0166	0.0249	0.0331	0.0414	0.0497	0.0580	0.0663
**14**	**B[a]P**	0.0033	0.0050	0.0083	0.0167	0.0250	0.0333	0.0416	0.0500	0.0583	0.0666
**15**	**DB[a,l]P**	0.0067	0.0100	0.0167	0.0333	0.0500	0.0667	0.0833	0.1000	0.1167	0.1333
**16**	**DB[ah]A**	0.0066	0.0100	0.0166	0.0332	0.0498	0.0664	0.0830	0.0996	0.1162	0.1328
**17**	**B[ghi]P**	0.0066	0.0099	0.0165	0.0330	0.0496	0.0661	0.0826	0.0991	0.1156	0.1321
**18**	**InP**	0.0003	0.0005	0.0008	0.0017	0.0025	0.0033	0.0042	0.0050	0.0058	0.0067

Abbreviations: Ace–Acenaphthene, Acen–Acenaphthylene, Ant–Anthracene, B[a]A–Benzo[a]anthracene, B[a]P–Benzo[a]pyrene, B[b]Ft+B[j]Ft–Benzo[b]fluoranthene+Benzo[j]fluoranthene, B[ghi]P–Benzo[ghi]perylene, B[k]Ft–Benzo[k]fluoranthene, Chry–Chrysene, DB[a,l]P–Dibenzo[a,l]pyrene, DB[ah]A–Dibenz[a,h]anthracene, Fln–Fluoranthene, Flu–Fluorene, InP–Indeno[1,2,3-cd]pyrene, Naph–Naphthalene, PAH–Polycyclic aromatic hydrocarbons, Phe–Phenanthrene, Pyr–Pyrene

Table S7. Recoveries obtained in ten fortification levels and the average recovery and relative standard deviation (RSD, %), for each polycyclic aromatic hydrocarbon (PAHs).

Polycyclic aromatic hydrocarbons (PAHs)		Recovery (%)										Average Recovery (%)	RSD (%)
Number PAHs	PAHs abbreviation	Spiking I	Spiking II	Spiking III	Spiking IV	Spiking V	Spiking VI	Spiking VII	Spiking VIII	Spiking IX	Spiking X		
1	Naph	83.1	88.8	85.7	87.6	88.1	93.8	90.0	90.7	88.4	87.3	88.3	3.26
2	Acen	78.9	78.9	80.0	80.5	80.2	80.9	80.6	78.5	79.2	85.3	80.3	2.42
3	Ace	83.1	87.6	88.9	91.0	89.3	90.8	87.3	87.2	87.5	86.4	87.9	2.62
4	Flu	85.5	83.5	85.2	87.6	85.6	88.6	85.3	85.4	85.3	84.9	85.7	1.66
5	Phe	93.9	96.1	98.2	95.8	103	97.8	101	102	105	111	100	5.06
6	Ant	99.7	110	102	99.4	99.7	107	99.8	101	100	100	102	3.51
7	Fln	99.5	105	108	109	107	108	108	102	105	107	106	3.03
8	Pyr	107	97.1	108	110	108	93.6	108	107	104	102	105	5.18
9	B[a]A	95.1	101	107	102	102	105	99.7	101	102	100	102	3.13
10	Chry	107	110.5	112	101	102	108	99.5	102	102	100	104	4.40
11+12	B[b]Ft+ B[j]Ft	98.3	97.0	98.0	99.3	99.2	105	101	101	100	100	99.9	2.23
13	B[k]Ft	96.0	102	97.0	100	98.7	104	98.9	98.1	98.4	98.0	99.1	2.44
14	B[a]P	94.6	92.5	102	97.8	98.1	103	97.7	97.1	97.7	97.1	97.7	3.10
15	DB[a,l]P	91.6	101	102	99.3	99.4	102	96.4	98.8	97.5	96.8	98.4	3.12
16	DB[ah]A	104	93.2	97.7	93.4	90.1	108	95.6	101	90.5	94.9	96.8	6.04
17	B[ghi]P	92.7	102	99.2	99.9	98.6	100	96.3	95.3	95.5	95.4	97.5	2.99
18	InP	103	93.9	91.0	107	99.9	98.5	96.4	97.6	96.3	97.9	98.1	4.56

Abbreviations: Ace–Acenaphthene, Acen–Acenaphthylene, Ant–Anthracene, B[a]A–Benzo[a]anthracene, B[a]P–Benzo[a]pyrene, B[b]Ft+B[j]Ft–Benzo[b]fluoranthene+Benzo[j]fluoranthene, B[ghi]P–Benzo[ghi]perylene, B[k]Ft–Benzo[k]fluoranthene, Chry–Chrysene, DB[a,l]P–Dibenzo[a,l]pyrene, DB[ah]A–Dibenz[a,h]anthracene, Fln–Fluoranthene, Flu–Fluorene, InP–Indeno[1,2,3-cd]pyrene, Naph–Naphthalene, PAH–Polycyclic aromatic hydrocarbons, Phe–Phenanthrene, Pyr–Pyrene, %RSD relative–standard deviation.

Table S8. Concentration ranges and number of calibration points used, and correlation coefficients (R), slope, Y-intercept, limits of detection (LOD), and limits of quantification (LOQ) obtained for the polycyclic aromatic hydrocarbons (PAHs) using calibration curves prepared in solvent.

**Polycyclic aromatic hydrocarbons (PAHs)**		**Number of calibration points**	**Concentration Range (μg/L)**		**Calibration Curve**				
**Number PAHs**	**PAHs abbreviation**		Minimum	Maximum	**R**	**Slope**	**Y-Intercept**	**LOD (μg/L)**	**LOQ (μg/L)**
**1**	**Naph**	7	5.00	200	0.99999	5.85E+03	3.59E+01	1.15	3.82
**2**	**Acen**	8	10.0	400	0.99999	8.83E+02	2.28E+02	1.99	6.62
**3**	**Ace**	8	5.00	200	0.99998	7.37E+03	-2.16E+03	1.47	4.88
**4**	**Flu**	7	0.493	19.8	0.99998	1.84E+05	6.84E+03	0.147	0.489
**5**	**Phe**	7	1.25	20.0	0.99994	4.15E+04	-2.96E+03	0.237	0.790
**6**	**Ant**	7	1.25	20.0	0.99987	4.41E+04	-1.54E+03	0.357	1.19
**7**	**Fln**	7	2.46	39.3	0.99996	8.44E+03	-6.04E+02	0.381	1.27
**8**	**Pyr**	7	1.21	19.3	0.99995	1.19E+04	1.11E+00	0.216	0.720
**9**	**B[a]A**	7	1.23	19.6	0.99991	2.78E+04	-9.70E+02	0.300	1.00
**10**	**Chry**	7	1,23	19.6	0.99995	1.07E+04	-1.87E+02	0.209	0.696
**11+12**	**B[b]Ft+ B[j]Ft**	7	4.99	79.9	0.99994	2.19E+04	-3.50E+03	0.957	3.19
**13**	**B[k]Ft**	7	1.24	19.9	0.99995	1.15E+05	-8.97E+03	0.222	0.741
**14**	**B[a]P**	7	1.25	20.0	0.99993	9.52E+04	-1.29E+04	0.257	0.857
**15**	**DB[a,l]P**	7	2.49	39.9	0.99996	1.90E+04	-5.35E+03	0.421	1.40
**16**	**DB[ah]A**	7	2,49	39.9	0.99992	6.73E+03	-1.29E+03	0.552	1.84
**17**	**B[ghi]P**	7	2.48	39.7	0.99991	2.68E+04	3.74E+03	0.587	1.96
**18**	**InP**	7	1.25	20.0	0.99994	1.06E+04	2.00E+02	0.245	0.817

Abbreviations: Ace–Acenaphthene, Acen–Acenaphthylene, Ant–Anthracene, B[a]A–Benzo[a]anthracene, B[a]P–Benzo[a]pyrene, B[b]Ft+B[j]Ft–Benzo[b]fluoranthene+Benzo[j]fluoranthene, B[ghi]P–Benzo[ghi]perylene, B[k]Ft–Benzo[k]fluoranthene, Chry–Chrysene, DB[a,l]P–Dibenzo[a,l]pyrene, DB[ah]A–Dibenz[a,h]anthracene, Fln–Fluoranthene , Flu–Fluorene, InP–Indeno[1,2,3-cd]pyrene, LOD–Limit of detection, LOQ–Limit of quantification, Naph–Naphthalene, PAH–Polycyclic aromatic hydrocarbons, Phe–Phenanthrene, Pyr–Pyrene, R–Correlation coefficient

Table S9. Concentration ranges (CR) and number of calibration points (NCP) used, correlation coefficient (R), slope, y-intercept, and method detection (MDL) and quantification (MQL) limits determined in the ocean sample extract for the polycyclic aromatic hydrocarbons (PAHs). The corresponding calibration parameters for the ocean water sample were calculated by applying the concentration factor and the sample volume.

Polycyclic aromatic hydrocarbons (PAHs)		NCP	R	Y-Intercept	Calibration Curve								EU Directive 105/2008		
					Ocean extract				Ocean sample						
**Number PAHs**	**PAHs abbreviation**				**CR extract**	**Slope**	**MDL extract**	**MQL extract**	**CR sample**	**Slope**	**MDL sample**	**MQL sample**	**AA (µg/L)**	**MAC (µg/L)**	DIRECTIVE 2008/105/EC of the European Parliament and of the Council of 16 December 2008 on environmental quality standards in the field of water policy, amending and subsequently repealing Council Directives 82/176/EEC, 83/513/EEC, 84/156/EEC, 84/491/EEC, 86/280/EEC and amending Directive 2000/60/EC of the European Parliament and of the Council. Official Journal of the European Union. L 348/84. 24.12.2008. available on: https://eur-lex.europa.eu/legal-content/EN/TXT/PDF/?uri=CELEX:32008L0105, accessed on 26 May 2026
**1**	**Naph**	7	0.99996	-2.10E+03	12.5-200	5.95E+03	1.43	4.76	16.7-334	1.78E+03	4.76	15.9	2.4		
**2**	**Acen**	7	0.99999	4.97E+02	25.0-400	9.00E+02	1.65	5.50	33.3-669	2.70E+02	5.50	18.3			
**3**	**Ace**	7	0.99995	-6.56E+03	12.5-200	7.48E+03	1.63	5.44	16.7-334	2.24E+03	5.44	18.1			
**4**	**Flu**	7	0.99974	-4.29E+04	2.46-39.4	1.93E+05	0.721	2.40	3.29-65.9	5.78E+04	2.40	8.01	0.1	1	
**5**	**Phe**	7	0.99984	-1.50E+04	1.25-20.0	4.47E+04	0.284	0.945	1.66-33.4	1.34E+04	0.945	3.15			
**6**	**Ant**	7	0.99982	-3.13E+03	1.25-20.0	4.52E+04	0.301	1.00	1.66-33.4	1.35E+04	1.00	3.34	0.1	0.4	
**7**	**Fln**	7	0.99977	-2.70E+03	2.46-39.3	8.63E+03	0.682	2.27	3.28-65.8	2.59E+03	2.27	7.58			
**8**	**Pyr**	7	0.99975	-1.88E+03	1.21-19.3	1.23E+04	0.347	1.16	4.02-64.4	3.70E+03	1.16	3.86			
**9**	**B[a]A**	7	0.99979	-5.25E+03	1.23-19.6	2.82E+04	0.325	1.08	1.63-32.8	8.46E+03	1.08	3.61			
**10**	**Chry**	7	0.99982	-9.28E+02	1.23-19.6	1.08E+04	0.296	0.986	1.63-32.8	3.25E+03	0.99	3.29			
**11+12**	**B[b]Ft+ B[j]Ft**	7	0.99979	-6.63E+03	4.99-79.9	2.22E+04	1.32	4.39	6.66-134	6.66E+03	4.39	14.6	0.03		
**13**	**B[k]Ft**	7	0.99977	-8.35E+03	1.24-19.9	1.16E+05	0.340	1.13	1.66-33.3	3.48E+04	1.13	3.78			
**14**	**B[a]P**	7	0.99973	-7.11E+03	1.25-20.0	1.07E+05	0.375	1.25	1.67-33.4	3.21E+04	1.25	4.17	0.05	0.1	
**15**	**DB[a,l]P**	7	0.99983	-2.18E+03	2.49-39.9	2.04E+04	0.595	1.98	3.32-66.7	6.11E+03	1.98	6.61			
**16**	**DB[ah]A**	7	0.99991	-8.72E+02	2.49-39.9	6.76E+03	0.440	1.47	8.31-133	2.03E+03	1.47	4.88			
**17**	**B[ghi]P**	7	0.99912	4.65E+04	2.48-39.7	2.76E+04	1.34	4.46	3.31-66.3	8.27E+03	4.46	14.9	0.002		
**18**	**InP**	5	0.99999	2.68E+03	1.25-20.0	1.11E+04	0.145	0.483	4.17-50.2	3.32E+03	0.483	1.61			

Abbreviations: AA–Annual Average, Ace–Acenaphthene, Acen–Acenaphthylene, Ant–Anthracene, B[a]A–Benzo[a]anthracene, B[a]P–Benzo[a]pyrene, B[b]Ft+B[j]Ft–Benzo[b]fluoranthene + Benzo[j]fluoranthene, B[ghi]P–Benzo[ghi]perylene, B[k]Ft–Benzo[k]fluoranthene, Chry–Chrysene, CR–Concentration Range, DB[a,l]P–Dibenzo[a,l]pyrene, DB[ah]A–Dibenz[a,h]anthracene, Fln–Fluoranthene, Flu–Fluorene, InP–Indeno[1,2,3-cd]pyrene, MAC–Maximum Admissible Concentration, MDL–method detection limit obtained in sample extract, MQL–method quantification limit, Naph–Naphthalene, NCP–Number of calibration points; Phe–Phenanthrene, Pyr–Pyrene, R–Correlation coefficient

Table S10. Concentration ranges (CR) and number of calibration points (NCP) used, correlation coefficients (R), slope, y-intercept, and method detection (MDL) and quantification (MQL) limits determined in the river sample extract for the polycyclic aromatic hydrocarbons (PAHs). The corresponding calibration parameters for the river water sample were calculated by applying the concentration factor and the sample volume.

**Polycyclic aromatic hydrocarbons (PAHs)**		**NCP**	**R**	**Y-Intercept**	**Calibration Curve**								EU Directive 105/2008		
					**River extract**				**River sample**						
**Number PAHs**	**PAHs abbreviation**				**CR extract**	**Slope**	**MDL extract**	**MQL extract**	**CR sample**	**Slope**	**MDL** sample	**MQL** sample	**AA** (µg/L)	**MAC** (µg/L)	DIRECTIVE 2008/105/EC of the European Parliament and of the Council of 16 December 2008 on environmental quality standards in the field of water policy, amending and subsequently repealing Council Directives 82/176/EEC, 83/513/EEC, 84/156/EEC, 84/491/EEC, 86/280/EEC and amending Directive 2000/60/EC of the European Parliament and of the Council. Official Journal of the European Union. L 348/84. 24.12.2008. available on: https://eur-lex.europa.eu/legal-content/EN/TXT/PDF/?uri=CELEX:32008L0105, accessed on 26 May 2026
**1**	**Naph**	6	0.99997	1.12E+04	5.00-100	5.76E+03	1.01	3.36	16.7-334	1.73E+03	3.36	11.2	2.4		
**2**	**Acen**	6	0.99999	3.09E+03	10.0-201	8.59E+02	2.79	9.30	33.3-669	2.58E+02	9.30	31.0			
**3**	**Ace**	6	1.00000	5.59E+03	5.00-100	7.29E+03	0.340	1.13	16.7-334	2.19E+03	1.13	3.77			
**4**	**Flu**	6	0.99997	3.79E+04	0.986-19.8	1.86E+05	0.176	0.588	3.29-65.9	5.58E+04	0.588	1.96	0.1	1	
**5**	**Phe**	7	0.99998	2.78E+04	0.499-20.0	4.11E+04	0.148	0.494	1.66-33.4	1.23E+04	0.494	1.65			
**6**	**Ant**	6	0.99994	4.96E+03	0.499-10.0	4.41E+04	0.138	0.459	1.66-33.4	1.32E+04	0.459	1.53	0.1	0.4	
**7**	**Fln**	6	0.99994	1.85E+03	0.983-19.7	8.30E+03	0.264	0.880	3.28-65.8	2.49E+03	0.880	2.93			
**8**	**Pyr**	7	0.99996	2.41E+03	1.21-19.3	1.17E+04	0.199	0.664	4.02-64.4	3.52E+03	0.664	2.21			
**9**	**B[a]A**	6	0.99996	2.30E+03	0.490-9.84	2.69E+04	0.113	0.378	1.6332.8-	8.07E+03	0.378	1.26			
**10**	**Chry**	6	0.99998	1.75E+03	0.490-9.84	1.04E+04	0.075	0.250	1.63-32.8	3.12E+03	0.250	0.835			
**11+12**	**B[b]Ft+ B[j]Ft**	6	0.99995	9.95E+03	2.00-40.1	2.14E+04	0.491	1.64	6.66-134	6.43E+03	1.64	5.46	0.03		
**13**	**B[k]Ft**	6	0.99997	1.52E+04	1.66-33.3	1.12E+05	0.101	0.336	1.66-33.3	3.36E+04	0.336	1.12			
**14**	**B[a]P**	6	0.99995	9.87E+03	0.500-10.0	1.02E+05	0.125	0.418	1.67-33.4	3.05E+04	0.418	1.39	0.05	0.1	
**15**	**DB[a,l]P**	6	0.99996	4.27E+03	1.00-20.0	1.97E+04	0.214	0.713	3.32-66.7	5.92E+03	0.713	2.38			
**16**	**DB[ah]A**	6	0.99997	1.65E+04	2.49-39.9	6.34E+03	0.402	1.34	8.31-133	1.90E+03	1.34	4.47			
**17**	**B[ghi]P**	6	0.99996	1.49E+05	0.992-19.9	2.60E+04	0.229	0.763	3.31-66.3	7.80E+03	0.763	2.54	0.002		
**18**	**InP**	6	0.99995	2.24E+03	1.25-15.1	1.18E+04	0.180	0.60	4.17-50.2	3.53E+03	0.60	2.00			

Abbreviations: AA–Annual Average, Ace–Acenaphthene, Acen–Acenaphthylene, Ant–Anthracene, B[a]A–Benzo[a]anthracene, B[a]P–Benzo[a]pyrene, B[b]Ft+B[j]Ft–Benzo[b]fluoranthene + Benzo[j]fluoranthene, B[ghi]P–Benzo[ghi]perylene, B[k]Ft–Benzo[k]fluoranthene, Chry–Chrysene, CR–Concentration Range, DB[a,l]P–Dibenzo[a,l]pyrene, DB[ah]A–Dibenz[a,h]anthracene, Fln–Fluoranthene, Flu–Fluorene, InP–Indeno[1,2,3-cd]pyrene, MAC–Maximum Admissible Concentration, MDL–method detection limit obtained in sample extract, MQL–method quantification limit, Naph–Naphthalene, NCP–Number of calibration points; Phe–Phenanthrene, Pyr–Pyrene, R–Correlation coefficient

Table S11. Concentration ranges (CR) and number of calibration points (NCP) used, correlation coefficients (R), slope, y-intercept, and method detection (MDL) and quantification (MQL) limits determined in the WWTP effluent sample extract for the polycyclic aromatic hydrocarbons (PAHs). The corresponding calibration parameters for the WWTP effluent water sample were calculated by applying the concentration factor and the sample volume.

Polycyclic aromatic hydrocarbons (PAHs)		NCP	R	Y-Intercept	Calibration Curve								EU Directive 105/2008		
					WWTP effluent extract				WWTP effluent sample						
Number PAHs	PAHs abbreviation				CR extract	Slope	MDL extract	MQL extract	CR sample	Slope	MDL sample	MQL sample	AA (µg/L)	MAC (µg/L)	DIRECTIVE 2008/105/EC of the European Parliament and of the Council of 16 December 2008 on environmental quality standards in the field of water policy, amending and subsequently repealing Council Directives 82/176/EEC, 83/513/EEC, 84/156/EEC, 84/491/EEC, 86/280/EEC and amending Directive 2000/60/EC of the European Parliament and of the Council. Official Journal of the European Union. L 348/84. 24.12.2008. available on: https://eur-lex.europa.eu/legal-content/EN/TXT/PDF/?uri=CELEX:32008L0105, accessed on 26 May 2026
1	Naph	7	0.99992	-9.88E+03	12.5-200	6.06E+03	2.88	9.61	41.7-667	1.82E+03	9.61	32.0	2.4		
2	Acen	7	0.99988	-1.11E+03	25.0-400	9.14E+02	6.89	23.0	83.3-1333	2.74E+02	23.0	76.6			
3	Ace	7	0.99992	-6.78E+03	12.5-200	7.29E+03	2.85	9.51	41.7-667	2.19E+03	9.51	31.7			
4	Flu	7	0.99991	-2.95E+04	2.46-39.4	1.92E+05	0.604	2.01	8.22-131	5.76E+04	2.01	6.71	0.1	1	
5	Phe	7	0.99990	-1.29E+03	1.25-20.0	4.43E+04	0.310	1.03	4.16-66.7	1.33E+04	1.03	3.45			
6	Ant	7	0.99990	-5.12E+03	1.25-20.0	4.55E+04	0.315	1.05	4.16-66.6	1.37E+04	1.05	3.49	0.1	0.4	
7	Fln	7	0.99987	-1.51E+03	2.46-39.3	8.48E+03	0.711	2.37	8.20-131	2.54E+03	2.37	7.90			
8	Pyr	7	0.99995	6.00E+02	1.21-19.3	1.19E+04	0.218	0.727	4.02-64.4	3.58E+03	0.727	2.42			
9	B[a]A	7	0.99991	-3.57E+03	1.23-19.6	2.82E+04	0.296	0.987	4.09-65.4	8.46E+03	0.987	3.29			
10	Chry	7	0.99990	-7.82E+02	1.23-19.6	1.07E+04	0.312	1.04	4.09-65.4	3.21E+03	1.04	3.47			
11+12	B[b]Ft+ B[j]Ft	7	0.99990	-9.23E+03	4.99-79.9	2.23E+04	1.27	4.25	16.6-266	6.68E+03	4.25	14.2	0.03		
13	B[k]Ft	7	0.99988	-1.34E+04	1.24-19.9	1.16E+05	0.350	1.17	4.15-66.3	3.49E+04	1.17	3.89			
14	B[a]P	7	0.99989	-1.30E+04	1.25-20.0	1.08E+05	0.337	1.12	4.17-66.7	3.24E+04	1.12	3.74	0.05	0.1	
15	DB[a,l]P	7	0.99993	-2.88E+03	2.49-39.9	2.03E+04	0.525	1.75	8.31-133	6.08E+03	1.75	5.83			
16	DB[ah]A	7	0.99994	-1.82E+03	2.49-39.9	6.56E+03	0.473	1.58	8.31-133	1.97E+03	1.58	5.25			
17	B[ghi]P	6	0.99993	6.05E+04	2.48-39.7	2.76E+04	0.559	1.86	8.27-132	8.28E+03	1.86	6.21	0.002		
18	InP	7	0.99997	5.57E+03	2.51-20.0	1.10E+04	0.192	0.64	4.17-66.7	3.31E+03	0.64	2.14			

Abbreviations: AA–Annual Average, Ace–Acenaphthene, Acen–Acenaphthylene, Ant–Anthracene, B[a]A–Benzo[a]anthracene, B[a]P–Benzo[a]pyrene, B[b]Ft+B[j]Ft–Benzo[b]fluoranthene + Benzo[j]fluoranthene, B[ghi]P–Benzo[ghi]perylene, B[k]Ft–Benzo[k]fluoranthene, Chry–Chrysene, CR–Concentration Range, DB[a,l]P–Dibenzo[a,l]pyrene, DB[ah]A–Dibenz[a,h]anthracene, Fln–Fluoranthene, Flu–Fluorene, InP–Indeno[1,2,3-cd]pyrene, MAC–Maximum Admissible Concentration, MDL–method detection limit obtained in sample extract, MQL–method quantification limit, Naph–Naphthalene, NCP–Number of calibration points; Phe–Phenanthrene, Pyr–Pyrene, R–Correlation coefficient

Table S12. Concentration ranges (CR) and number of calibration points (NCP) used, correlation coefficients (R), slope, y-intercept, and method detection (MDL) and quantification (MQL) limits determined in the WWTP influent sample extract for the polycyclic aromatic hydrocarbons (PAHs). The corresponding calibration parameters for the WWTP influent water sample were calculated by applying the concentration factor and the sample volume.

Polycyclic aromatic hydrocarbons (PAHs)		NCP	R	Y-Intercept	Calibration Curve								EU Directive 105/2008		
					WWTP influent extract				WWTP influent sample						
Number PAHs	PAHs abbreviation				CR extract	Slope	MDL extract	MQL extract	CR sample	Slope	MDL sample	MQL sample	AA (μg/L)	MAC (μg/L)	DIRECTIVE 2008/105/EC of the European Parliament and of the Council of 16 December 2008 on environmental quality standards in the field of water policy, amending and subsequently repealing Council Directives 82/176/EEC, 83/513/EEC, 84/156/EEC, 84/491/EEC, 86/280/EEC and amending Directive 2000/60/EC of the European Parliament and of the Council. Official Journal of the European Union. L 348/84. 24.12.2008. available on: https://eur-lex.europa.eu/legal-content/EN/TXT/PDF/?uri=CELEX:32008L0105, accessed on 26 May 2026
1	**Naph**	6	0.99996	7.06E+03	12.5-200	6.19E+03	2.26	7.54	41.7-667	1.86E+03	7.54	25.1	2.4		
2	**Acen**	7	0.99988	5.25E+03	25.0-400	9.38E+02	6.84	22.8	83.3-1333	2.81E+02	22.8	76.0			
3	**Ace**	7	0.99988	2.21E+04	12.5-200	7.29E+03	3.53	11.8	41.7-667	2.19E+03	11.8	39.2			
4	**Flu**	7	0.99987	2.40E+04	2.46-39.4	1.96E+05	0.711	2.37	8.22-131	5.88E+04	2.37	7.90	0.1	1	
5	**Phe**	7	0.99992	7.25E+03	1.25-20.0	4.34E+04	0.280	0.934	4.16-66.5	1.30E+04	0.934	3.11			
6	**Ant**	7	0.99988	6.13E+03	1.25-20.0	4.60E+04	0.348	1.16	4.16-66.6	1.38E+04	1.16	3.86	0.1	0.4	
7	**Fln**	7	0.99634	7.56E+03	2.46-39.3	7.97E+03	3.78	12.6	8.20-131	2.39E+03	12.6	42.0			
8	**Pyr**	7	0.99995	-6.07E+02	1.21-19.3	1.24E+04	0.224	0.746	4.02-64.4	3.72E+03	0.746	2.49			
9	**B[a]A**	7	0.99994	-1.94E+02	1.23-19.6	2.87E+04	0.248	0.828	4.09-65.4	8.62E+03	0.828	2.76			
10	**Chry**	7	0.99994	5.77E+01	1.23-19.6	1.12E+04	0.240	0.800	4.09-65.4	3.35E+03	0.800	2.67			
11+12	**B[b]Ft+ B[j]Ft**	7	0.99993	5.64E+03	4.99-79.9	2.26E+04	1.08	3.60	16.6-266	6.79E+03	3.60	12.0	0.03		
13	**B[k]Ft**	7	0.99990	4.22E+03	1.24-19.9	1.19E+05	0.322	1.07	4.15-66.3	3.56E+04	1.07	3.58			
14	**B[a]P**	7	0.99985	-5.03E+02	1.25-20.0	1.12E+05	0.385	1.28	4.17-66.7	3.37E+04	1.28	4.28	0.05	0.1	
15	**DB[a,l]P**	7	0.99992	8.73E+02	2.49-39.9	2.11E+04	0.568	1.89	8.31-133	6.32E+03	1.89	6.31			
16	**DB[ah]A**	7	0.99994	-1.13E+03	2.49-39.9	7.00E+03	0.472	1.57	8.31-133	2.10E+03	1.57	5.24			
17	**B[ghi]P**	7	0.99989	-1.61E+03	2.48-39.7	3.07E+04	0.648	2.16	8.27-132	9.20E+03	2.16	7.20	0.002		
18	**InP**	6	0.99997	1.22E+04	2.51-20.0	1.05E+04	0.188	0.627	8.38-66.7	3.14E+03	0.63	2.09			

Abbreviations: AA–Annual Average, Ace–Acenaphthene, Acen–Acenaphthylene, Ant–Anthracene, B[a]A–Benzo[a]anthracene, B[a]P–Benzo[a]pyrene, B[b]Ft+B[j]Ft–Benzo[b]fluoranthene + Benzo[j]fluoranthene, B[ghi]P–Benzo[ghi]perylene, B[k]Ft–Benzo[k]fluoranthene, Chry–Chrysene, CR–Concentration Range, DB[a,l]P–Dibenzo[a,l]pyrene, DB[ah]A–Dibenz[a,h]anthracene, Fln–Fluoranthene, Flu–Fluorene, InP–Indeno[1,2,3-cd]pyrene, MAC–Maximum Admissible Concentration, MDL–method detection limit obtained in sample extract, MQL–method quantification limit, Naph–Naphthalene, NCP–Number of calibration points; Phe–Phenanthrene, Pyr–Pyrene, R–Correlation coefficient

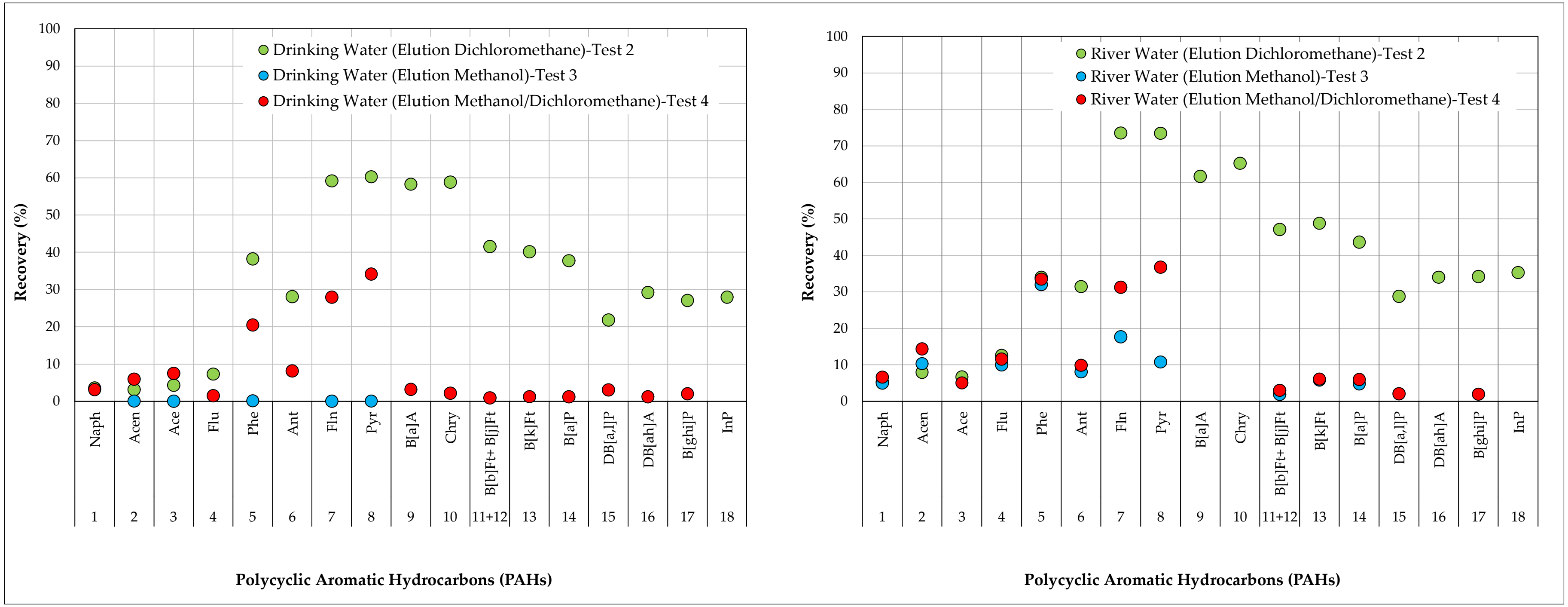

Figure S1. Recoveries of 18 polycyclic aromatic hydrocarbons (PAHs) from river water using Strata-X cartridges across Tests 2, 3, and 4 with different elution solvents: dichloromethane, methanol, and a dichloromethane:methanol (1:1, v/v) mixture.

Abbreviations: Ace–Acenaphthene, Acen–Acenaphthylene, Ant–Anthracene, B[a]A–Benzo[a]anthracene, B[a]P–Benzo[a]pyrene, B[b]Ft+B[j]Ft–Benzo[b]fluoranthene+Benzo[j]fluoranthene, B[ghi]P–Benzo[ghi]perylene, B[k]Ft–Benzo[k]fluoranthene, Chry–Chrysene, DB[a,l]P–Dibenzo[a,l]pyrene, DB[ah]A–Dibenz[a,h]anthracene, Fln–Fluoranthene, Flu–Fluorene, InP–Indeno[1,2,3-cd]pyrene, Naph–Naphthalene, PAH–Polycyclic aromatic hydrocarbons, Phe–Phenanthrene, Pyr–Pyrene.

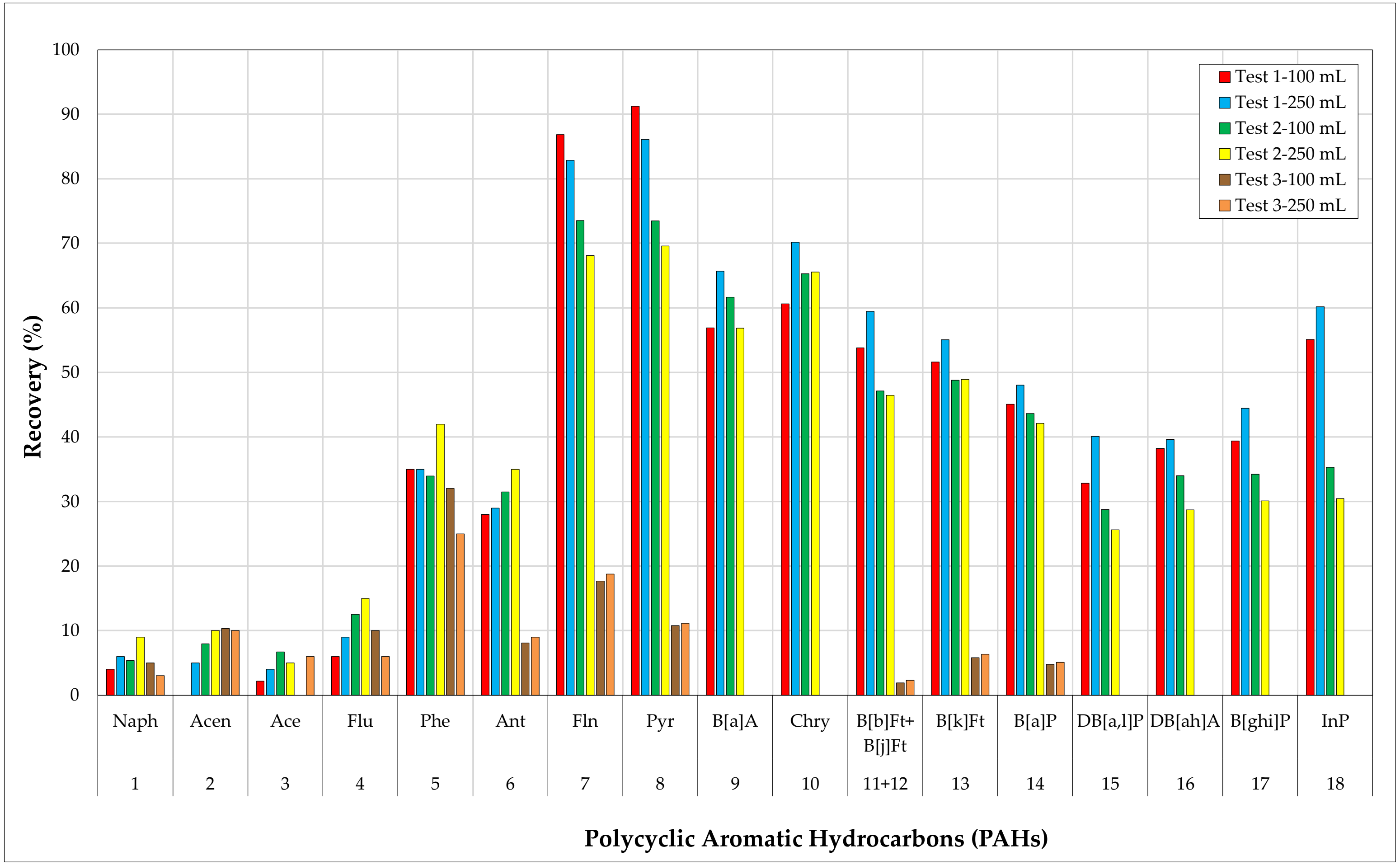

Figure S2. Recovery percentages of the studied polycyclic aromatic hydrocarbons (PAHs) from river water (RW) samples (100 mL and 250 mL) using Tests 1, 2, and 3.

Abbreviations: Ace–Acenaphthene, Acen–Acenaphthylene, Ant–Anthracene, B[a]A–Benzo[a]anthracene, B[a]P–Benzo[a]pyrene, B[b]Ft+B[j]Ft–Benzo[b]fluoranthene+Benzo[j]fluoranthene, B[ghi]P–Benzo[ghi]perylene, B[k]Ft–Benzo[k]fluoranthene, Chry–Chrysene, DB[a,l]P–Dibenzo[a,l]pyrene, DB[ah]A–Dibenz[a,h]anthracene, Fln–Fluoranthene, Flu–Fluorene, InP–Indeno[1,2,3-cd]pyrene, Naph–Naphthalene, PAH–Polycyclic aromatic hydrocarbons, Phe–Phenanthrene, Pyr–Pyrene.

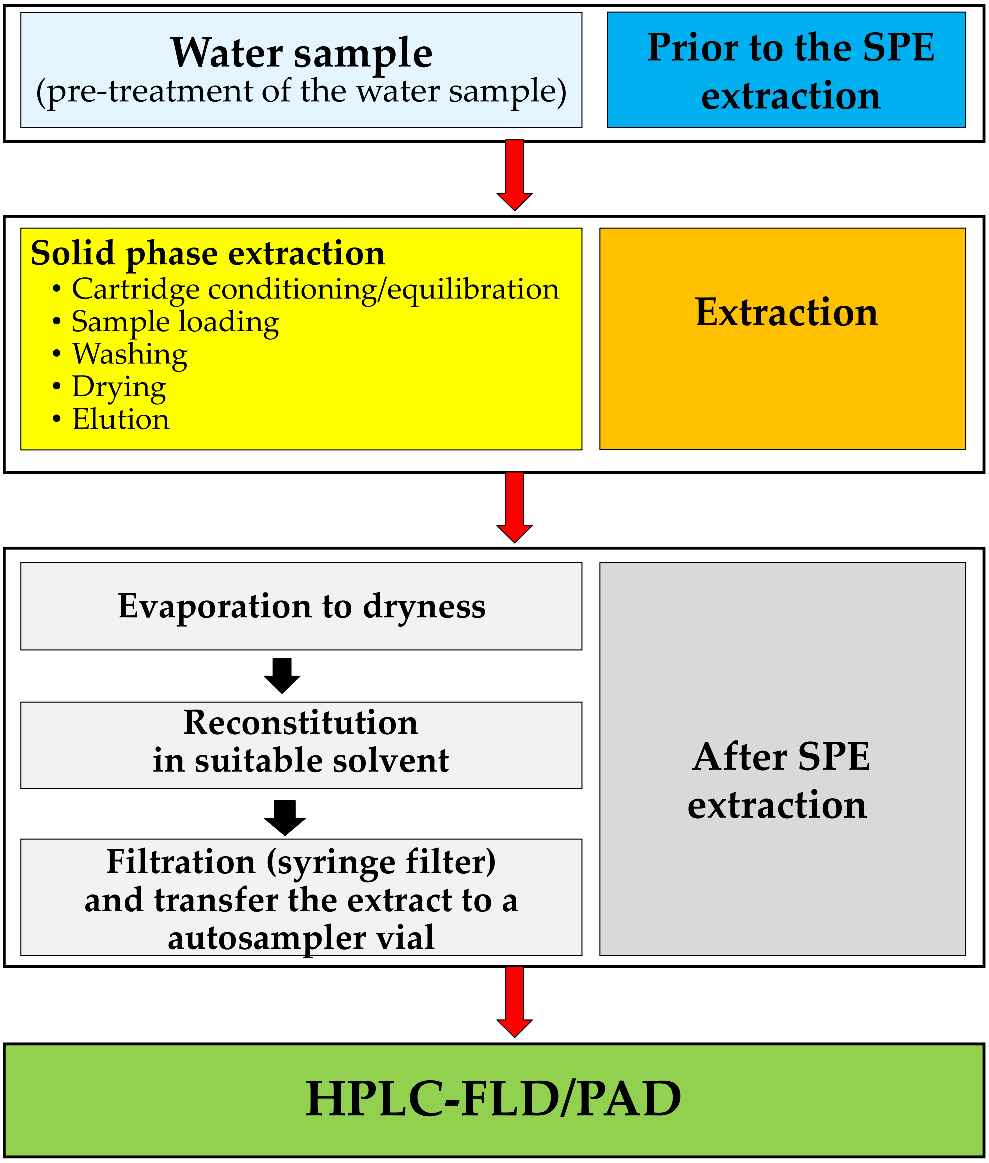

Figure S3. General procedure used for the extraction of polycyclic aromatic hydrocarbons (PAHs) in water samples.

HPLC-FLD

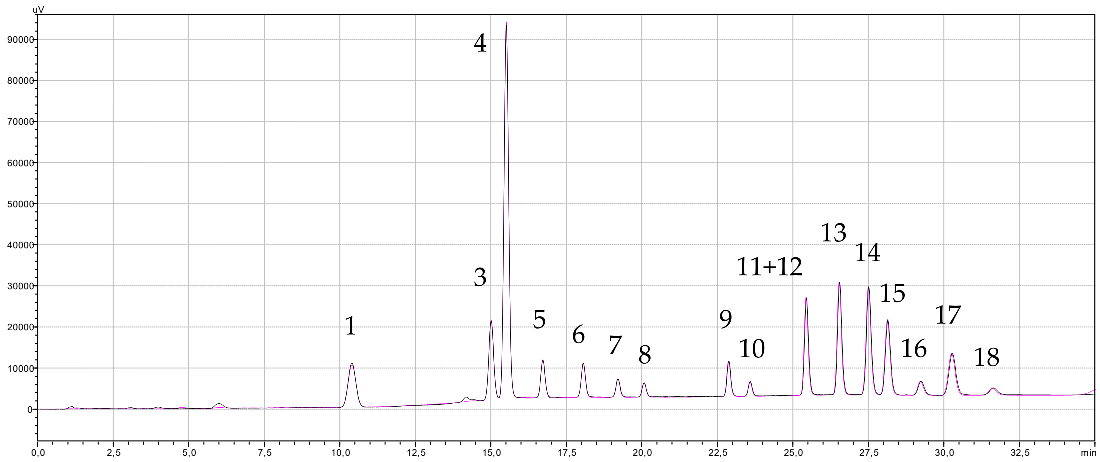

HPLC-PAD

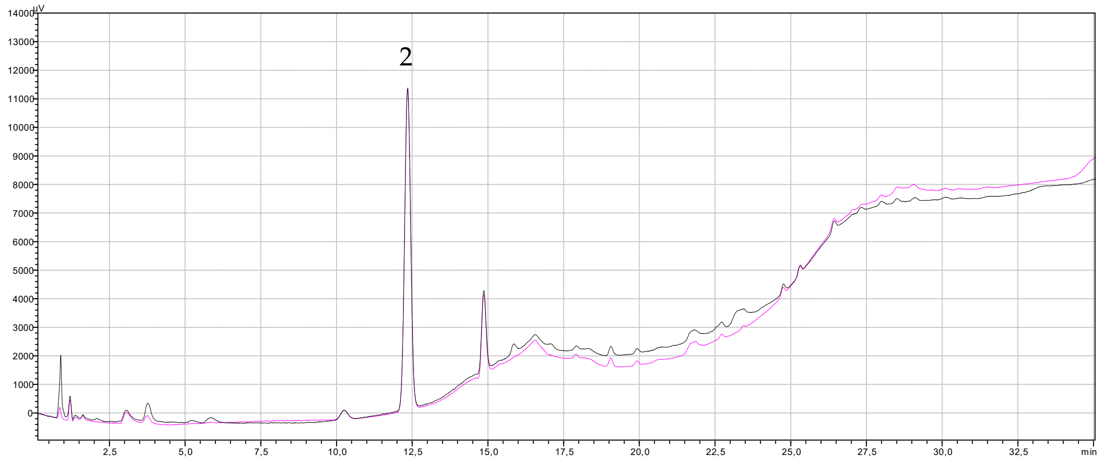

Figure S4. Overlay chromatogram of a standard mixture of polycyclic aromatic hydrocarbons (PAHs) without PAHs standard filtration (black) and with PAHs standard filtration using a PTFE syringe filter (pink).

Legend: 1–Naphthalene, 2–Acenaphthylene, 3–Acenaphthene, 4–Fluorene, 5–Phenanthrene, 6–Anthracene, 7–Fluoranthene, 8–Pyrene, 9–Benzo[a]anthracene, 10–Chrysene, 11+12–Benzo[b]fluoranthene+Benzo[j]fluoranthene, 13–Benzo[k]fluoranthene, 14–B[a]P–Benzo[a]pyrene, 15–Dibenzo[a,l]pyrene, 16–DB[ah]A–Dibenz[a,h]anthracene, 17–Benzo[ghi]perylene, 18–Indeno[1,2,3-cd]pyrene

Legend:
1-Naph, 2-Acen, 3-Ace, 4-Flu, 5-Phe, 6-Ant, 7-Fln, 8-Pyr, 9-B[a]A, 10- Chry, 11+12-B[b]Ft+ B[j]Ft, 13-B[k]Ft, 14-B[a]P, 15-DB[a,l]P, 16-DB[ah]A, 17-B[ghi]P, and 18-InP

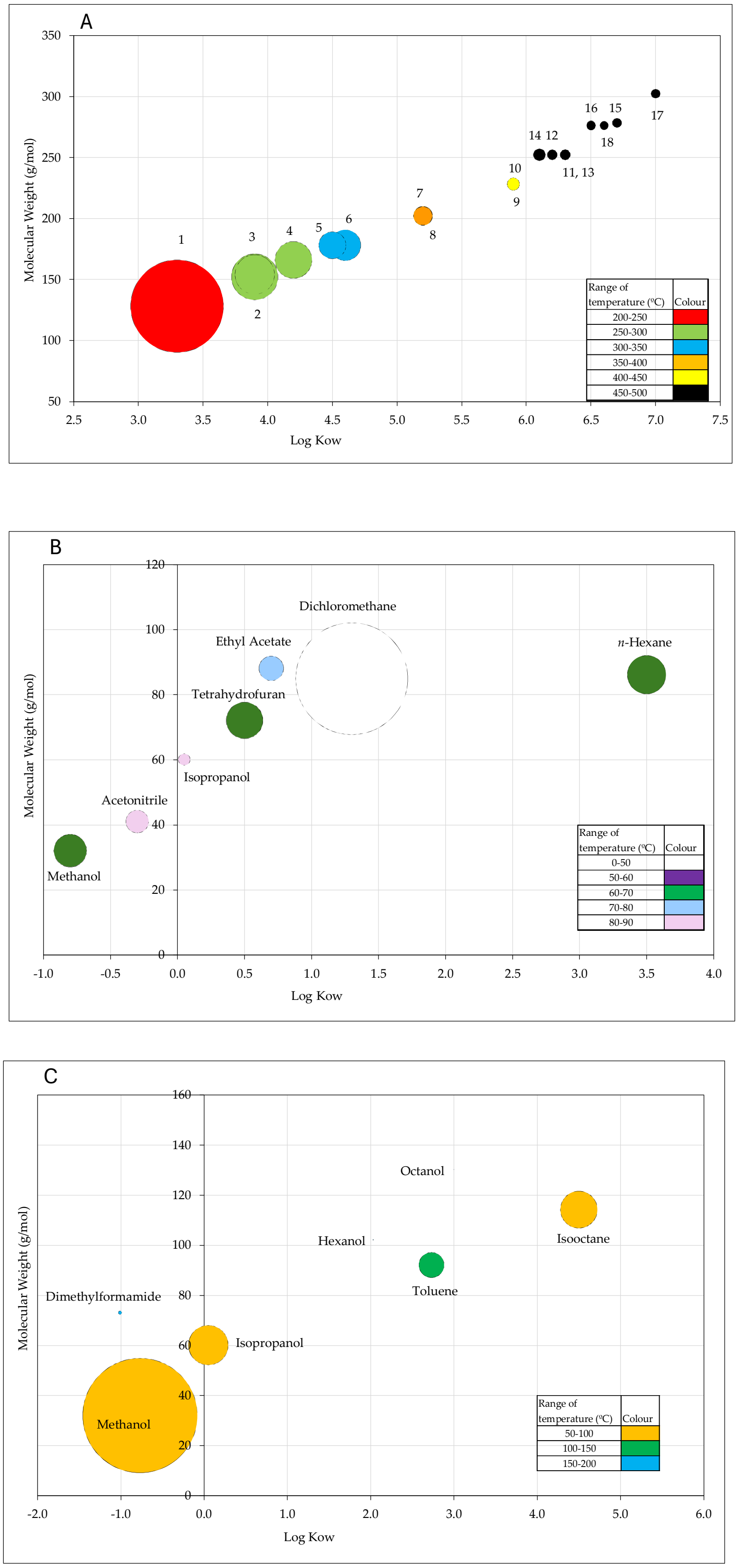

Figure S5. Physicochemical properties of the studied polycyclic aromatic hydrocarbon (PAH) compounds (A), primary solvents (B), and keepers (C) (Molecular Weight, log Kow, boiling point, and vapor pressure were obtained from https://pubchem.ncbi.nlm.nih.gov/).

Abbreviations: Ace–Acenaphthene, Acen–Acenaphthylene, Ant–Anthracene, B[a]A–Benzo[a]anthracene, B[a]P–Benzo[a]pyrene, B[b]Ft+B[j]Ft–Benzo[b]fluoranthene+Benzo[j]fluoranthene, B[ghi]P–Benzo[ghi]perylene, B[k]Ft–Benzo[k]fluoranthene, Chry–Chrysene, DB[a,l]P–Dibenzo[a,l]pyrene, DB[ah]A–Dibenz[a,h]anthracene, Fln–Fluoranthene, Flu–Fluorene, InP–Indeno[1,2,3-cd]pyrene, Kow–octanol–water partition coefficient, Naph–Naphthalene, Phe–Phenanthrene, Pyr–Pyrene.

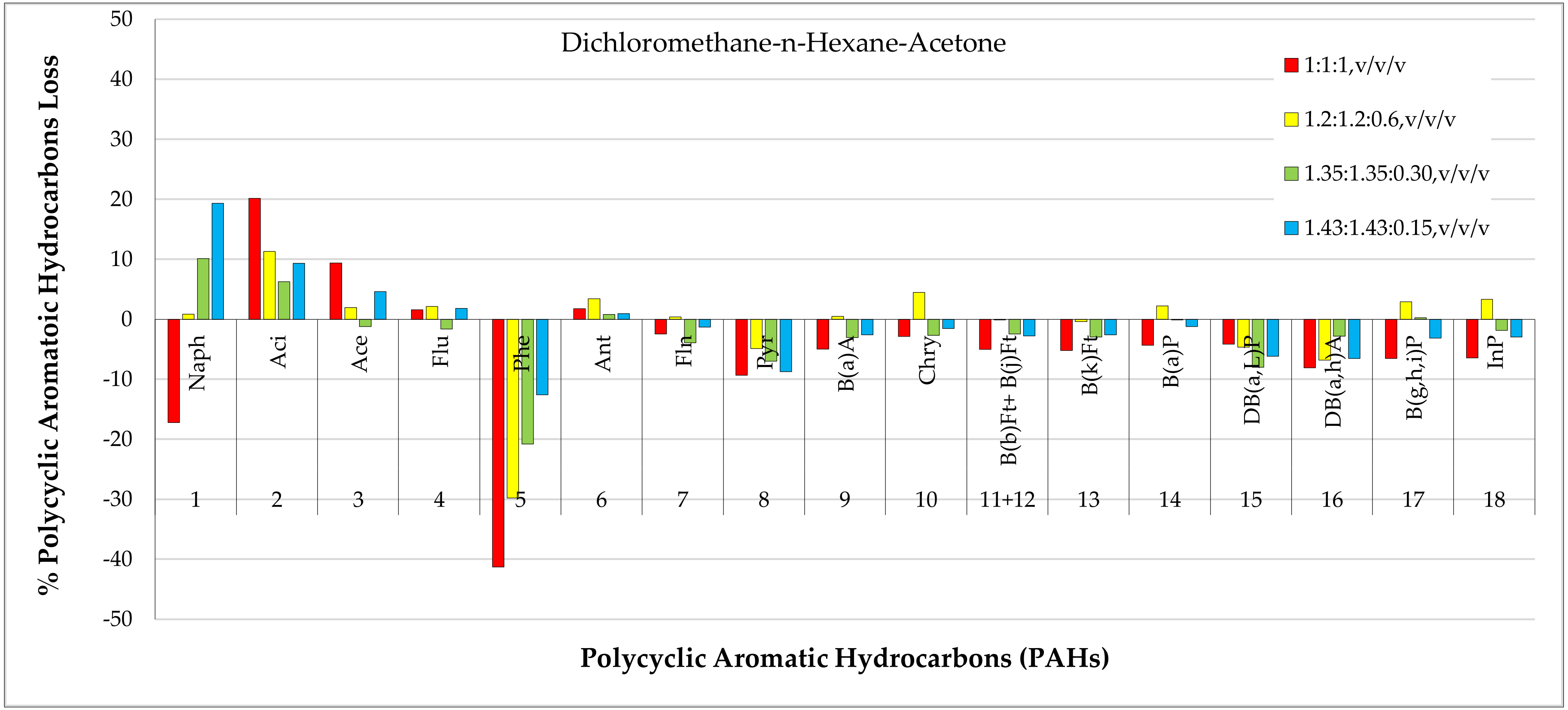

Figure S6. Percentage loss for each polycyclic aromatic hydrocarbons (PAHs) in the different proportions of dichloromethane-*n*-hexane-acetone.

Abbreviations: Ace–Acenaphthene, Acen–Acenaphthylene, Ant–Anthracene, B[a]A–Benzo[a]anthracene, B[a]P–Benzo[a]pyrene, B[b]Ft+B[j]Ft–Benzo[b]fluoranthene+Benzo[j]fluoranthene, B[ghi]P–Benzo[ghi]perylene, B[k]Ft–Benzo[k]fluoranthene, Chry–Chrysene, DB[a,l]P–Dibenzo[a,l]pyrene, DB[ah]A–Dibenz[a,h]anthracene, Fln–Fluoranthene, Flu–Fluorene, InP–Indeno[1,2,3-cd]pyrene, Naph–Naphthalene, PAH–Polycyclic aromatic hydrocarbons, Phe–Phenanthrene, Pyr–Pyrene.

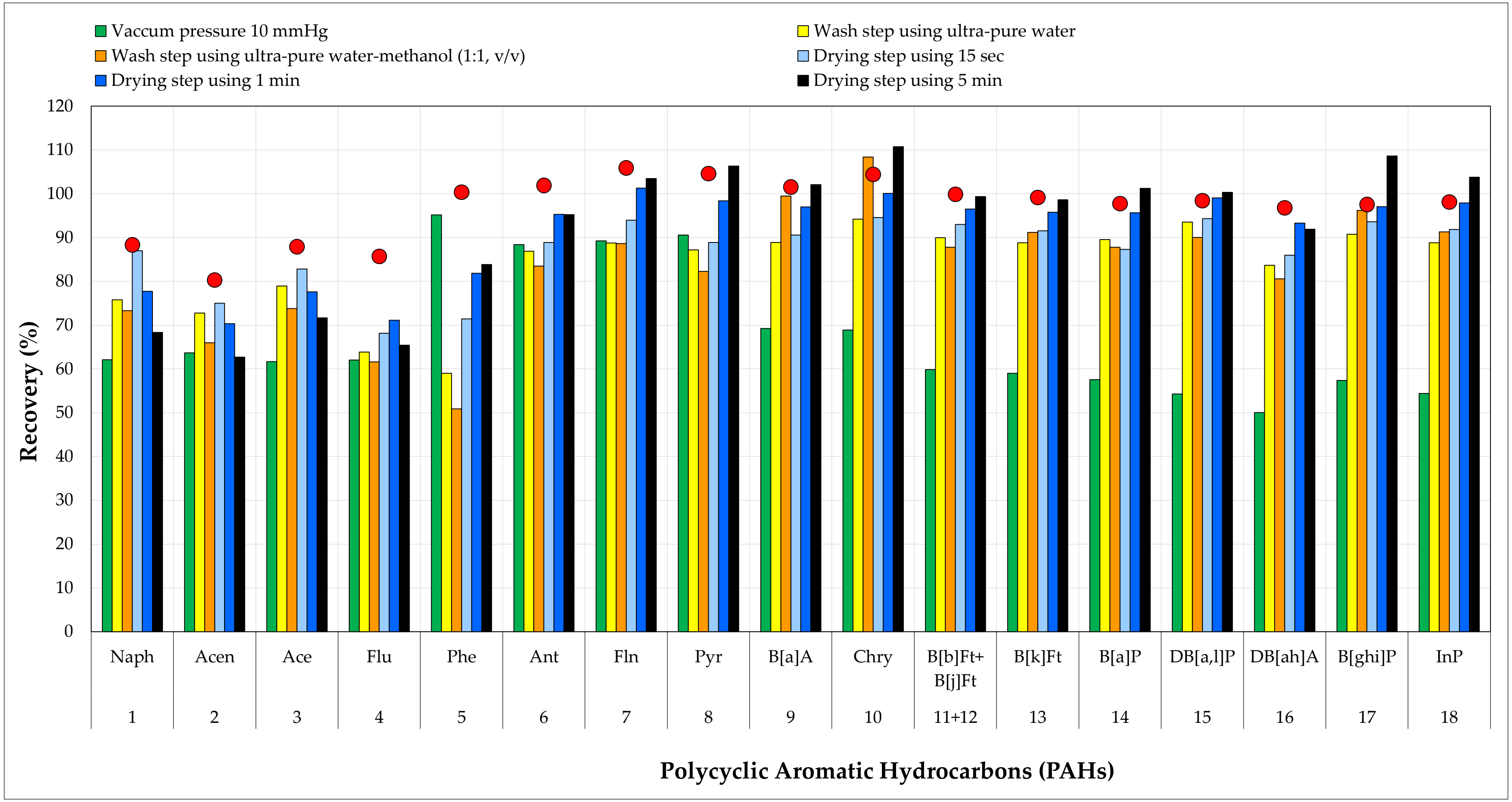

Figure S7. Evaluation of the impact of SPE operational steps (vacuum pressure, cartridge washing, and drying duration) on polycyclic aromatic hydrocarbons (PAHs) recoveries during method optimization.

Abbreviations: Ace–Acenaphthene, Acen–Acenaphthylene, Ant–Anthracene, B[a]A–Benzo[a]anthracene, B[a]P–Benzo[a]pyrene, B[b]Ft+B[j]Ft–Benzo[b]fluoranthene+Benzo[j]fluoranthene, B[ghi]P–Benzo[ghi]perylene, B[k]Ft–Benzo[k]fluoranthene, Chry–Chrysene, DB[a,l]P–Dibenzo[a,l]pyrene, DB[ah]A–Dibenz[a,h]anthracene, Fln–Fluoranthene, Flu–Fluorene, InP–Indeno[1,2,3-cd]pyrene, Naph–Naphthalene, PAH–Polycyclic aromatic hydrocarbons, Phe–Phenanthrene, Pyr–Pyrene.

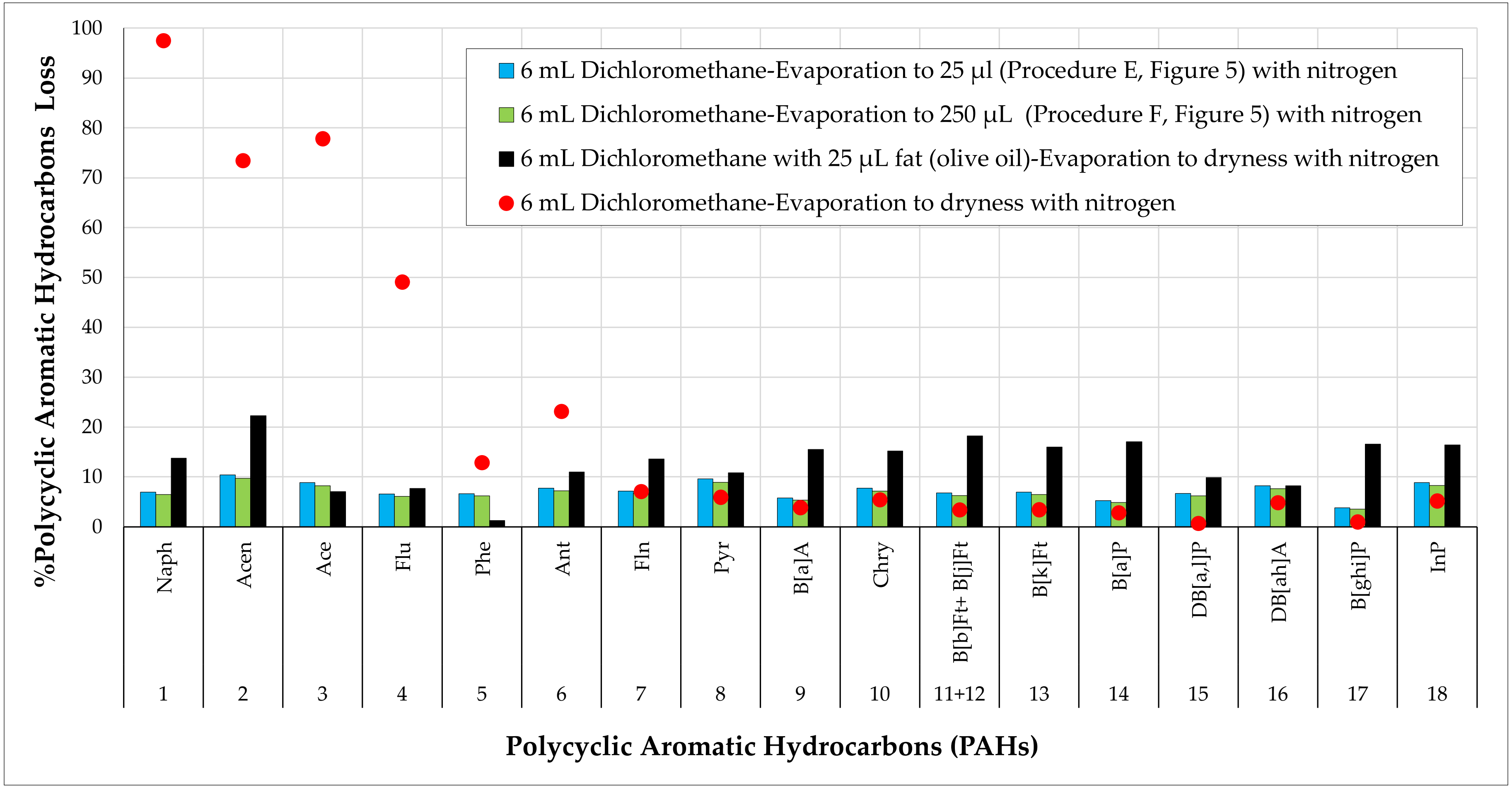

Figure S8. Percentage of polycyclic aromatic hydrocarbons (PAHs) loss during various evaporation protocols: evaporation to dryness (with and without a lipid 'keeper'), evaporation to a final volume of 25 µL, and a two-stage evaporation process to a final volume of 250 µL.

Abbreviations: Ace–Acenaphthene, Acen–Acenaphthylene, Ant–Anthracene, B[a]A–Benzo[a]anthracene, B[a]P–Benzo[a]pyrene, B[b]Ft+B[j]Ft–Benzo[b]fluoranthene+Benzo[j]fluoranthene, B[ghi]P–Benzo[ghi]perylene, B[k]Ft–Benzo[k]fluoranthene, Chry–Chrysene, DB[a,l]P–Dibenzo[a,l]pyrene, DB[ah]A–Dibenz[a,h]anthracene, Fln–Fluoranthene, Flu–Fluorene, InP–Indeno[1,2,3-cd]pyrene, Naph–Naphthalene, Phe–Phenanthrene, Pyr–Pyrene.
